# Gemcitabine and cisplatin plus nivolumab as organ-sparing treatment for muscle-invasive bladder cancer: a phase 2 trial

**DOI:** 10.1038/s41591-023-02568-1

**Published:** 2023-10-02

**Authors:** Matthew D. Galsky, Siamak Daneshmand, Sudeh Izadmehr, Edgar Gonzalez-Kozlova, Kevin G. Chan, Sara Lewis, Bassam El Achkar, Tanya B. Dorff, Jeremy Paul Cetnar, Brock O. Neil, Anishka D’Souza, Ronac Mamtani, Christos Kyriakopoulos, Tomi Jun, Mahalya Gogerly-Moragoda, Rachel Brody, Hui Xie, Kai Nie, Geoffrey Kelly, Amir Horowitz, Yayoi Kinoshita, Ethan Ellis, Yohei Nose, Giorgio Ioannou, Rafael Cabal, Diane M. Del Valle, G. Kenneth Haines, Li Wang, Kent W. Mouw, Robert M. Samstein, Reza Mehrazin, Nina Bhardwaj, Menggang Yu, Qianqian Zhao, Seunghee Kim-Schulze, Robert Sebra, Jun Zhu, Sacha Gnjatic, John Sfakianos, Sumanta K. Pal

**Affiliations:** 1 Division of Hematology and Medical Oncology, Icahn School of Medicine at Mount Sinai, New York, NY USA; 2 Tisch Cancer Institute, Icahn School of Medicine at Mount Sinai, New York, NY USA; 3 Department of Urology, Keck School of Medicine of USC, Norris Comprehensive Cancer Center, Los Angeles, CA USA; 4 Department of Oncological Sciences, Tisch Cancer Institute, Icahn School of Medicine at Mount Sinai, New York, NY USA; 5 Department of Urology, City of Hope Comprehensive Cancer Center, Duarte, CA USA; 6 Department of Radiology, Tisch Cancer Institute, Icahn School of Medicine at Mount Sinai, New York, NY USA; 7 Department of Medical Oncology & Therapeutics, City of Hope Comprehensive Cancer Center, Duarte, CA USA; 8 Division of Hematology and Medical Oncology, Oregon Health and Science University, Portland, OR USA; 9 Department of Urology, University of Utah, Salt Lake City, UT USA; 10 Division of Hematology and Medical Oncology, Keck School of Medicine of USC, Norris Comprehensive Cancer Center, Los Angeles, CA USA; 11 Division of Hematology and Medical Oncology, University of Pennsylvania Abramson Cancer Center, Philadelphia, PA USA; 12 Division of Hematology and Medical Oncology, University of Wisconsin Carbone Cancer Center, Madison, WI USA; 13 Genentech, South San Francisco, CA USA; 14 Department of Pathology, Tisch Cancer Institute, Icahn School of Medicine at Mount Sinai, New York, NY USA; 15 Human Immune Monitoring Center, Icahn School of Medicine at Mount Sinai, New York, NY USA; 16 Precision Immunology Institute, Icahn School of Medicine at Mount Sinai, New York, NY USA; 17 Department of Genetics and Genomic Sciences, Icahn School of Medicine at Mount Sinai, New York, NY USA; 18 Icahn Institute for Data Science and Genomic Technology, Icahn School of Medicine at Mount Sinai, New York, NY USA; 19 Department of Pathology, Molecular and Cell-based Medicine, Icahn School of Medicine at Mount Sinai, New York, NY USA; 20 Gene Dx, Stamford, CT USA; 21 Department of Radiation Oncology, Dana-Farber Cancer Institute/Brigham & Women’s Hospital, Harvard Medical School, Boston, MA USA; 22 Department of Radiation Oncology, Tisch Cancer Institute, Icahn School of Medicine at Mount Sinai, New York, NY USA; 23 Department of Urology, Tisch Cancer Institute, Icahn School of Medicine at Mount Sinai, New York, NY USA; 24 Department of Biostatistics and Medical Informatics, University of Wisconsin Carbone Cancer Center, Madison, WI USA; 25 Present Address: Formerly with the Icahn School of Medicine at Mount Sinai, New York, NY USA

**Keywords:** Phase II trials, Translational research, Bladder cancer, Cancer immunotherapy, Chemotherapy

## Abstract

Cystectomy is a standard treatment for muscle-invasive bladder cancer (MIBC), but it is life-altering. We initiated a phase 2 study in which patients with MIBC received four cycles of gemcitabine, cisplatin, plus nivolumab followed by clinical restaging. Patients achieving a clinical complete response (cCR) could proceed without cystectomy. The co-primary objectives were to assess the cCR rate and the positive predictive value of cCR for a composite outcome: 2-year metastasis-free survival in patients forgoing immediate cystectomy or <ypT1N0 in patients electing immediate cystectomy. Seventy-six patients were enrolled; of these, 33 achieved a cCR (43%, 95% confidence interval (CI): 32%, 55%), and 32 of 33 who achieved a cCR opted to forgo immediate cystectomy. The positive predictive value of cCR was 0.97 (95% CI: 0.91, 1), meeting the co-primary objective. The most common adverse events were fatigue, anemia, neutropenia and nausea. Somatic alterations in pre-specified genes (*ATM*, *RB1*, *FANCC* and *ERCC2*) or increased tumor mutational burden did not improve the positive predictive value of cCR. Exploratory analyses of peripheral blood mass cytometry and soluble protein analytes demonstrated an association between the baseline and on-treatment immune contexture with clinical outcomes. Stringently defined cCR after gemcitabine, cisplatin, plus nivolumab facilitated bladder sparing and warrants further study. ClinicalTrials.gov identifier: NCT03558087.

## Abstract (web summary)

In a phase 2 trial, the combination of gemcitabine, cisplatin and anti-PD-1 led to a clinical complete response in 43% of patients with muscle-invasive bladder cancer, which facilitated bladder sparing and was associated with long-term bladder-intact metastasis-free survival.

## Main

Radical cystectomy is a standard treatment for muscle-invasive bladder cancer (MIBC). However, radical cystectomy is a life-changing operation due to the need for urinary diversion and is associated with a 90-d mortality risk of up to 6–8% (ref. ^1^). Neoadjuvant cisplatin-based chemotherapy before radical cystectomy confers improved survival in patients with MIBC^2,3^. Although the intent of neoadjuvant chemotherapy is eradication of micrometastatic disease, neoadjuvant cisplatin-based chemotherapy after transurethral resection of bladder tumor (TURBT) yields a pathological complete response (pCR) at the time of cystectomy in approximately 30% of patients^2,4^. Paradoxically, a pCR can be determined only after the bladder has been surgically removed.

Given the potential to achieve a pCR with TURBT followed by neoadjuvant chemotherapy, the need for cystectomy to achieve cure in all patients has been questioned. Early single-center retrospective studies reported that long-term bladder-intact disease-free survival is achievable in a select subset of patients with MIBC treated with TURBT plus systemic therapy, and contemporary retrospective series have substantiated such results^5–7^. However, challenges to the broader application of this treatment paradigm have included (1) a paucity of prospective studies^8,9^; (2) a lack of rigorous and standardized approaches to both measure (that is, clinical restaging) and define clinical complete response (cCR); (3) poor understanding of the impact of later cystectomy on cancer control in patients with a cCR who develop local recurrence after a period of initial surveillance; and (4) suboptimal systemic therapeutic regimens.

Single-agent PD-1/PD-L1 immune checkpoint blockade followed by cystectomy for the treatment of MIBC has been shown to yield a pCR in 30–40% of patients^10,11^. Cisplatin may induce favorable immunomodulatory effects^12^, providing rationale for regimens combining neoadjuvant chemotherapy plus PD-1/PD-L1 blockade. In phase 2 studies, neoadjuvant gemcitabine, cisplatin, plus PD-1/PD-L1 blockade has demonstrated pCR rates of 40–50%, leading to the initiation of several phase 3 trials (NCT03661320, NCT03732677 and NCT03924856)^13,14^.

The integration of molecular biomarkers may further improve selection of patients with MIBC who could be treated definitively with TURBT plus systemic therapy. Somatic alterations in genes encoding proteins involved in DNA damage repair (DDR) in pre-treatment TURBT tissue have been correlated with a higher pCR rate with cisplatin-based neoadjuvant chemotherapy^15–20^. DDR gene alterations have also been associated with an increased likelihood of response to PD-1/PD-L1 blockade, potentially mediated by increased tumor mutational burden (TMB), raising the hypothesis that such tumors may be particularly sensitive to cisplatin plus PD-1/PD-L1 blockade combination regimens^21,22^.

**Extended Data Fig. 1:**
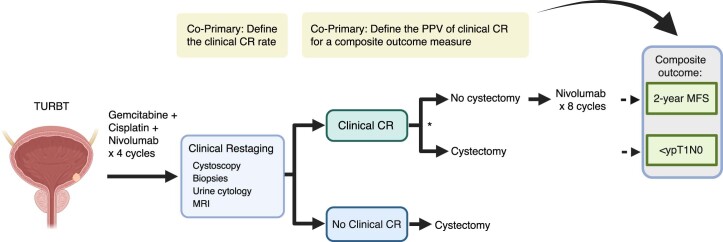
Schematic representation of the design of HCRN GU16-257. Patients with muscle-invasive urothelial cancer of the bladder diagnosed based on standard of care TURBT (transurethral resection of bladder tumor) received four cycles of gemcitabine, cisplatin, plus nivolumab followed by clinical restaging consisting of cystoscopy with biopsies, urine cytology, and imaging including MRI (magnetic resonance imaging) of the bladder (or computed tomography scan if MRI was contraindicated). Patients achieving a clinical complete response (cCR) were offered the option to proceed with immediate cystectomy versus proceed without cystectomy and receive an additional 4 months of single-agent nivolumab followed by surveillance. Patients without a cCR were recommended to undergo immediate cystectomy. The primary objectives were to estimate the cCR rate and to assess the positive predictive value (PPV) of cCR for a composite outcome measure (MFS, metastasis-free survival). *Patients achieving a clinical CR were offered the option to forgo cystectomy or proceed with immediate cystectomy.

To further evaluate the role of TURBT plus systemic therapy as definitive treatment for MIBC, we designed a phase 2 trial integrating (1) cisplatin-based chemotherapy plus PD-1 blockade; (2) standardized clinical restaging; and (3) translational analyses seeking to explore genomic, radiologic and immunologic biomarkers to refine future patient selection for this approach. Our primary goal was to test whether uniformly assessed and consistently defined cCR could identify patients who could safely forgo immediate cystectomy. We reasoned that a potentially effective personalized risk-adapted strategy would (1) tolerate missing some patients who might have been suitable candidates to forgo immediate cystectomy in favor of maximizing identification of patients who fare well without immediate cystectomy and (2) incorporate the ability of later cystectomy to achieve favorable cancer-related outcomes in the subset of patients with a cCR who experience local recurrence after initial surveillance. Therefore, our primary objectives were to estimate the cCR rate and to assess the positive predictive value of cCR for a composite outcome measure (2-year metastasis-free survival in patients forgoing immediate cystectomy or <ypT1N0 in patients electing immediate cystectomy; Extended Data Fig. 1).

## Results

### Patient characteristics and treatment

Cisplatin-eligible patients with cT2–T4aN0M0 MIBC received treatment with four cycles of gemcitabine and cisplatin plus nivolumab (Extended Data Fig. 1) followed by clinical restaging. Clinical restaging comprised magnetic resonance imaging (MRI) of the abdomen and pelvis (unless contraindicated, in which case computed tomography (CT) scans were substituted), CT of the chest, cystoscopy with biopsies according to a recommended template (Methods) and urine cytology. A cCR was defined as (1) no evidence of high-grade malignancy on biopsy; (2) no malignant cells on urine cytology; and (3) no definitive evidence of local or metastatic disease on cross-sectional imaging. Patients achieving a cCR were offered the option to proceed with cystectomy versus retain their bladder and receive eight additional doses of nivolumab (administered every 2 weeks) followed by surveillance. Patients not achieving a cCR were recommended to proceed with cystectomy.

**Fig. 1:**
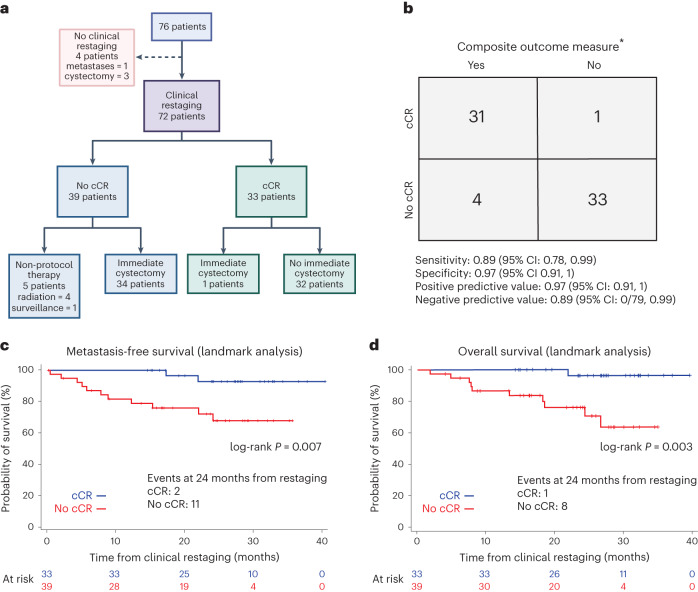
Study design and primary objectives of HCRN GU16-257. **a**, CONSORT diagram outlining disposition of patients enrolled on HCRN GU16-257 and demonstrating co-primary objective of estimating the cCR rate. **b**, Contingency table informing co-primary objective of assessing the positive predictive value of cCR for the composite outcome measure of 2-year metastasis-free survival in patients forgoing immediate cystectomy or <ypT1N0 in patients undergoing immediate cystectomy (*n* = 69 of 76 total patients). Seven patients were excluded for the following reasons: four patients who did not undergo clinical response assessment; two patients who did not achieve a cCR, who did not pursue cystectomy and who were lost to follow-up before 2 years; and one patient who achieved a cCR and without evidence of local or distant recurrence at 18 months and who was subsequently lost to follow-up. **c**, Metastasis-free survival according to cCR versus no cCR using landmark timepoint of post-cycle 4 restaging (*n* = 72; four patients were excluded who did not undergo clinical response assessment). Estimating metastasis-free survival was a secondary objective of the study. **d**, Overall survival according to cCR versus no cCR using landmark timepoint of post-cycle 4 restaging (*n* = 72; four patients were excluded who did not undergo clinical response assessment). Estimating overall survival was a secondary objective of the study. **a** and **b** were created with BioRender. *Composite outcome measure: 2-year metastasis-free survival in patients forgoing immediate cystectomy or <ypT1N0 in patients undergoing immediate cystectomy.

Between 8 August 2018 and 24 November 2020, 76 patients were enrolled with baseline characteristics as detailed in Table 1. The disposition of patients on study is outlined in Fig. 1a. Among the 76 patients enrolled, 72 underwent clinical restaging: one patient did not undergo restaging due to the development of metastatic disease, and three patients developed adverse events (cerebrovascular accident, deep venous thrombosis and increase in creatinine) and proceeded with cystectomy.

**Table 1:** Baseline patient characteristics (*n* = 76)

Characteristic	Category	*n*	% or range
Sex	Female	16	21%
	Male	60	79%
Race	Caucasian	58	76%
	African American	1	1%
	Asian	9	12%
	Unknown	8	11%
Age (years)	Median	69	39–85
Clinical stage	cT2N0M0	43	57%
	cT3N0M0	24	32%
	cT4N0M0	9	12%
Histology	UC	57	75%
	UC with squamous	7	9%
	UC with glandular	2	3%
	UC with micropapillary	6	8%
	UC with other variant	4	5%

### Coprimary endpoint analysis

The co-primary endpoint of cCR was achieved in 33 of 76 patients (43%, 95% confidence interval (CI): 32%, 55%; Fig. 1a). Lower baseline clinical T stage was associated with a higher likelihood of a cCR, although cCRs were observed in patients with cT2–T4 disease (Extended Data Table 1). Among the 33 patients achieving a cCR, only one opted for immediate cystectomy with surgical pathology, revealing a low-grade ypTaN0 urothelial cancer (UC). As shown in Fig. 1b, the positive predictive value of cCR (co-primary endpoint) for the composite outcome measure was 0.97 (95% CI: 0.91, 1), with the lower bound of the 95% CI exceeding the pre-specified threshold of 80%.

The median metastasis-free and overall survival for the entire study cohort was not reached at the time of the data lock (secondary endpoints). To further contextualize the prognostic impact of achieving a cCR as related to metastasis-free survival and overall survival, a post hoc landmark analysis was performed using the time of clinical restaging as ‘time 0’. On landmark analysis from the time of restaging, patients achieving a cCR experienced significantly longer metastasis-free survival and overall survival compared to patients not achieving a cCR (Fig. 1c,d).

### Clinical outcomes according to cCR status

**Fig. 2:**
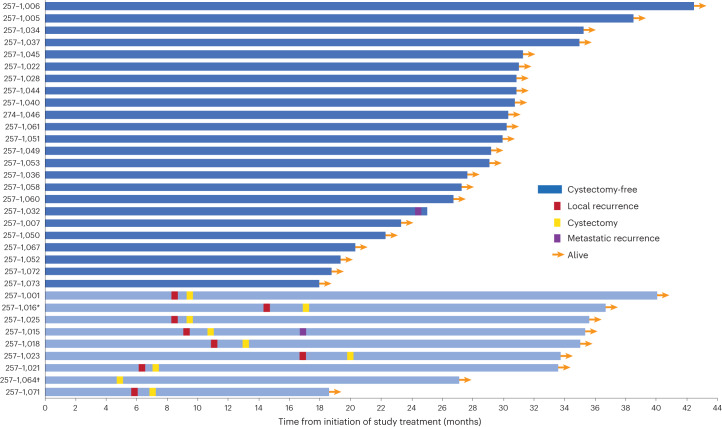
Clinical outcomes of patients enrolled on HCRN GU16-257 achieving a cCR. * Patient underwent cystectomy for radiographic changes concerning for local recurrence without evidence of cancer on biopsy or final cystectomy specimen. † Patient opted for immediate cystectomy.

The median follow-up for patients achieving a cCR was 30 months (range, 18–42 months) at the data lock and the clinical outcomes of this group are detailed in Fig. 2. Thirty-two patients opting to forgo immediate cystectomy received a median of eight (range, 0–8) cycles of maintenance nivolumab, and eight of 32 patients later underwent cystectomy for local recurrence (including one patient for an abnormal MRI scan with no cancer detected on TURBT or cystectomy). The clinical stage at the time of recurrence and the pathological stage at cystectomy are summarized in Supplementary Table 1; seven of eight patients had ≤ypT2N0 disease on cystectomy. Two additional patients developed non-invasive local recurrence during follow-up (low-grade cTa and cTis) and were managed with TURBT and intravesical BCG, respectively, without evidence of subsequent recurrence. Two of the 32 patients developed metastatic disease, including one patient with metastatic disease diagnosed 10 months after a cystectomy revealed ypT4N1 disease and the other presenting with malignant ascites with no evidence of recurrence in the bladder.

Thirty-nine patients did not achieve a cCR, and 34 of 39 underwent cystectomy (four received off protocol radiation and one declined any local therapy). The relationship between clinical restaging results in patients not achieving a cCR and the final cystectomy pathological stage is summarized in Supplementary Table 2.

### Safety

The treatment-emergent adverse events are detailed in Extended Data Table 2 and Supplementary Table 4. Grade ≥3 treatment-emergent adverse events occurred in 75% of patients. The most common all-grade treatment-emergent adverse events were fatigue, anemia, neutropenia and nausea, and the most common grade ≥3 treatment-emergent adverse events were anemia, neutropenia and urinary tract infections. One patient died due to sepsis subsequent to a bowel perforation occurring at the time of cystectomy, which was not attributed to systemic therapy.

### Genomic features associated with clinical outcomes

**Fig. 3:**
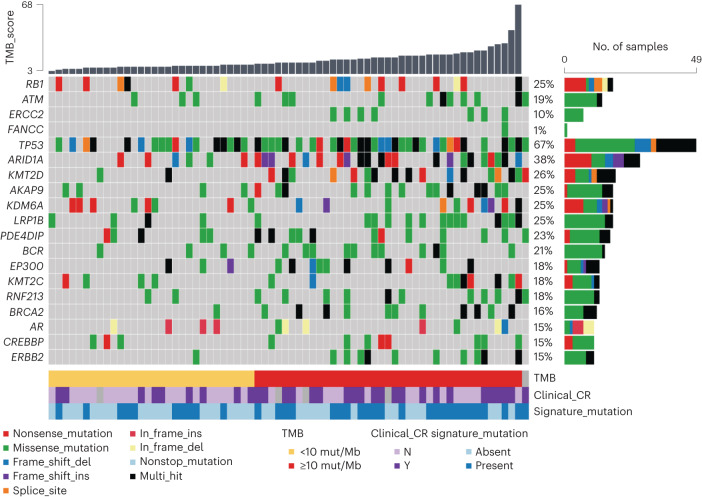
Genomic alterations in pre-treatment tumor tissue and association with clinical outcomes. Oncoplot revealing frequently mutated genes based on DNA sequencing of pre-treatment transurethral resection of bladder tumor specimens among 73 patients. Oncoplot is arranged according to TMB (Methods) and annotated based on the presence or absence of a mutation in a pre-specified set of genes (*RB1*, *ATM*, *ERCC2* and *FANCC*), referred to as ‘Signature_mutations’ and according to cCR categorization. Mutations are annotated: del, deletion; ins, insertion.

In an effort to refine future selection of patients for this risk-adapted treatment approach, a secondary objective of the study was to assess whether the presence of a set of genomic alterations in baseline TURBT tissue would enhance the positive predictive value of cCR. Tumor-only targeted DNA sequencing of pre-treatment TURBT tissue was available from 73 of 76 patients (Fig. 3). A panel of genes that, when mutated, had previously been correlated with response to cisplatin-based chemotherapy or PD-1/PD-L1 blockade (*ERCC2*, *RB1*, *ATM* and *FANCC*)^15–22^, as well as increased TMB (using an established cutpoint of ≥10 mutations per megabase (mut/Mb), which has served as the basis for tumor-agnostic PD-1 blockade regulatory approvals and for which sensitivity and specificity in bladder cancer has been established^23,24^), was pre-specified for analysis. Similar to the co-primary objective, the intent of this secondary objective was to assess the positive predictive value of the genomic alterations, added to cCR, for the composite outcome measure of 2-year metastasis-free survival in patients forgoing immediate cystectomy or <ypT1N0 in patients undergoing immediate cystectomy. However, the high positive predictive value of cCR alone precluded this analysis, and, instead, the positive predictive value of cCR with or without the pre-specified genomic alterations for the composite outcome of 2-year bladder-intact survival in patients forgoing immediate cystectomy or ≤ypT1N0 in patients undergoing immediate cystectomy was explored. As shown in Table 2 (and associated contingency table, Supplementary Table 4), the positive predictive value of the pre-specified genomic alterations added to cCR status did not clearly enhance the positive predictive value of cCR alone. The possible exception was the presence of a pathogenic mutation in *FANCC*, *ATM* and/or *RB1* in patients with a cCR, which was limited to five patients, all of whom had pathogenic *RB1* mutations; the relevance of this finding is unclear.

**Table 2:** Performance characteristics of cCR with or without genomic alterations in baseline TURBT tissue for a composite outcome measure of 2-year bladder-intact survival in patients forgoing immediate cystectomy or <ypT1N0 in patients undergoing immediate cystectomy

Measure	Performance metric
**cCR**	
Sensitivity	0.85 (95% CI: 0.72, 1.00)
Specificity	0.79 (95% CI: 0.66, 0.91)
Positive predictive value	0.72 (95% CI: 0.56, 0.87)
Negative predictive value	0.89 (95% CI: 0.79, 0.99)
**cCR + any mutation in** ***FANCC***, ***ATM*** **and/or** ***RB1***	
Sensitivity	0.31 (95% CI: 0.13, 0.49)
Specificity	0.93 (95% CI: 0.84, 1.00)
Positive predictive value	0.73 (95% CI: 0.46, 0.99)
Negative predictive value	0.67 (95% CI: 0.55, 0.80)
**cCR + any pathogenic**^**a**^ **mutation in** ***FANCC***, ***ATM*** **and/or** ***RB1***	
Sensitivity	0.19 (95% CI: 0.04, 0.34)
Specificity	1.00 (95% CI: 1.00, 1.00)
Positive predictive value	1.00 (95% CI: 1.00, 1.00)
Negative predictive value	0.66 (95% CI: 0.54, 0.78)
**cCR + any mutation in** ***ERCC2***	
Sensitivity	0.12 (95% CI: 0.00, 0.24)
Specificity	0.95 (95% CI: 0.88, 1.00)
Positive predictive value	0.60 (95% CI: 0.17, 1.00)
Negative predictive value	0.62 (95% CI: 0.50, 0.74)
**cCR + any pathogenic**^**a**^ **mutation** ***ERCC2***	
Sensitivity	0.08 (95% CI: 0.00, 0.18)
Specificity	0.95 (95% CI: 0.88, 1.00)
Positive predictive value	0.50 (95% CI: 0.01, 0.99)
Negative predictive value	0.61 (95% CI: 0.49, 0.73)
**cCR + any mutation in** ***FANCC***, ***ATM***, ***RB1*** **and/or** ***ERCC2***	
Sensitivity	0.38 (95% CI: 0.20, 0.57)
Specificity	0.90 (95% CI: 0.81, 0.99)
Positive predictive value	0.71 (95% CI: 0.48, 0.95)
Negative predictive value	0.69 (95% CI: 0.57, 0.82)
**cCR + any pathogenic**^**a**^ **mutation in** ***FANCC***, ***ATM***, ***RB1*** **and/or** ***ERCC2***	
Sensitivity	0.23 (95% CI: 0.07, 0.39)
Specificity	0.95 (95% CI: 0.88, 1.00)
Positive predictive value	0.75 (95% CI: 0.45, 1.00)
Negative predictive value	0.66 (95% CI: 0.53, 0.78)
**cCR + TMB ≥10 mut/Mb**	
Sensitivity	0.63 (95% CI: 0.43, 0.82)
Specificity	0.85 (95% CI: 0.74, 0.96)
Positive predictive value	0.71 (95% CI: 0.52, 0.91)
Negative predictive value	0.79 (95% CI: 0.67, 0.91)
**cCR + any** ***FANCC***, ***ATM***, ***RB1***, ***ERCC2***, **and/or TMB ≥ 10 mut/Mb**	
Sensitivity	0.61 (95% CI: 0.43, 0.80)
Specificity	0.85 (95% CI: 0.74, 0.96)
Positive predictive value	0.73 (95% CI: 0.54, 0.91)
Negative predictive value	0.77 (95% CI: 0.65, 0.90)

^a^At least presumed pathogenic mutation, as defined in the Methods.

An exploratory analysis was also performed to assess the association between the pre-specified genomic alterations and achieving a cCR. cCR rates were higher in patients with tumors harboring *ERCC2* mutations or TMB ≥10 mut/Mb versus patients with tumors without such alterations, but these associations did not achieve statistical significance after correction for false discovery (Extended Data Table 3).

### Radiographic features associated with clinical outcomes

**Extended Data Fig. 2:**
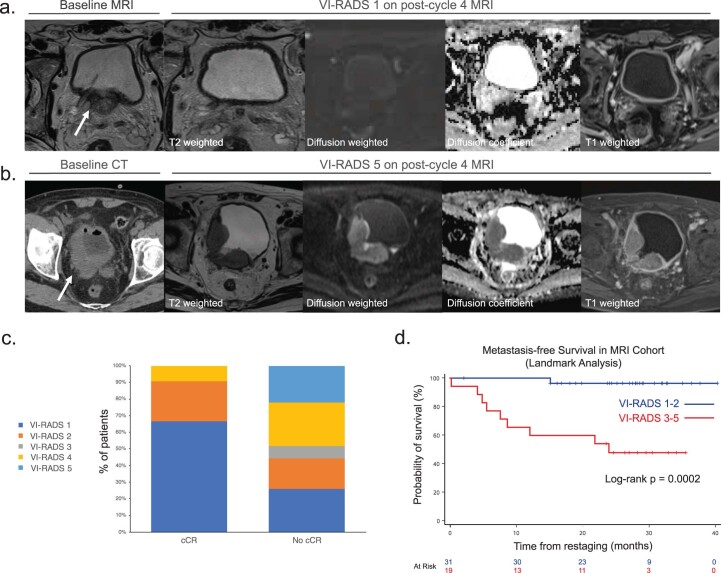
Vesical Imaging-Reporting And Data System (VI-RADS) scoring of bladder magnetic resonance imaging (MRI) post-treatment with gemcitabine, cisplatin, plus nivolumab and association with clinical outcomes. (**a**) Representative baseline image demonstrating posterior bladder wall mass and post- gemcitabine, cisplatin, plus nivolumab MRI sequences scored as VI-RADS 1. (**b**) Representative baseline image demonstrating posterior bladder wall mass and post- gemcitabine, cisplatin, plus nivolumab MRI sequences scored as VI-RADS 5. (**c**) Distribution of VI-RADS scores on MRI of the bladder post- gemcitabine, cisplatin, plus nivolumab (n = 50). (**d**) Landmark analysis for metastasis-free survival in study cohort with available restaging MRI scans (n = 50) according to VI-RADS 1-2 versus 3–5.

Conventional radiographic assessments are largely qualitative, and bladder tumors are particularly difficult to assess given the anatomy of the bladder and challenges distinguishing post-treatment bladder wall thickening from residual tumor^25^. Post-cycle-4 restaging MRI scans were recommended per protocol (unless otherwise contraindicated or not feasible, in which case CT scans were substituted) and were obtained in 50 of 76 patients. An exploratory analysis was performed involving central review of the MRI images with assignment of Vesical Imaging-Reporting and Data System (VI-RADS) scores^25^ (Extended Data Fig. 2a,b) by two independent reviewers blinded to clinical outcomes (weighted kappa: 0.63; 95% CI: 0.44, 0.82). The distribution of VI-RADS scores at the time of restaging, according to cCR status, is shown in Extended Data Fig. 2c. Only two patients who achieved a cCR had a restaging VI-RADS score greater than 2, although both experienced a subsequent local recurrence. Although VI-RADS scores of 1–2 versus 3–5 were enriched in patients achieving a cCR, 44% of patients not achieving a cCR had restaging VI-RADS scores of 1–2 (Extended Data Fig. 2c). On landmark analysis from the time of restaging, restaging VI-RADS score of ≤2 versus >2 was associated with significantly longer metastasis-free survival (*P* = 0.0002 from log-rank test; Extended Data Fig. 2d).

### Immunological features associated with clinical outcomes

**Fig. 4:**
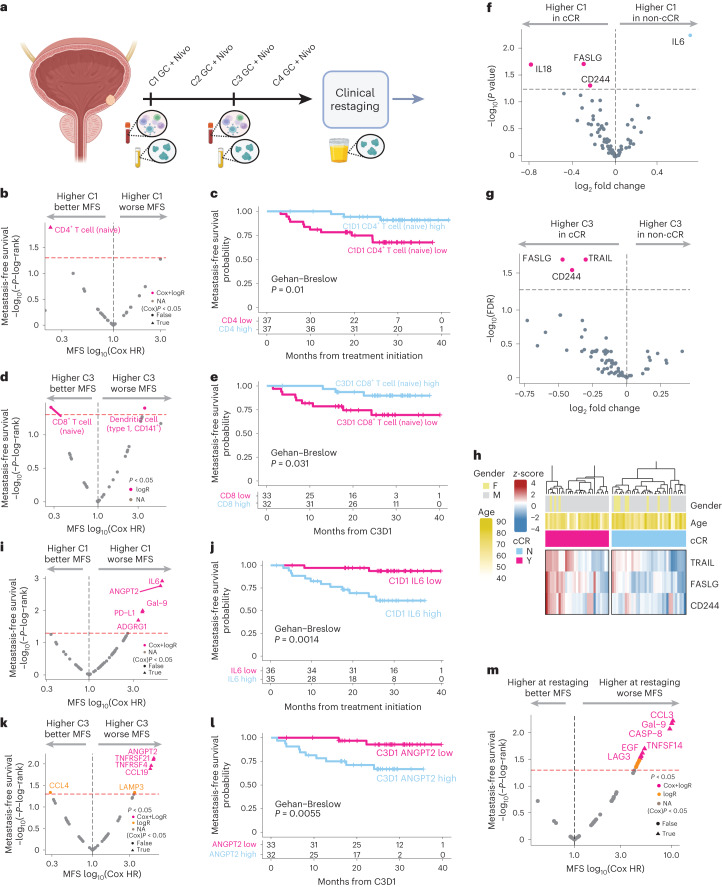
Association between peripheral blood immune populations as determined by mass cytometry (CyTOF) or peripheral blood or urine protein analytes and cCR or metastasis-free survival. **a**, Timing of sample collection. **b**, Volcano plot for metastasis-free survival (MFS) based on C1D1 peripheral blood CyTOF data (*n* = 74; patients without samples or outcome data were excluded) showing log-rank test (*y* axis) and Cox regression hazard ratio (*x* axis). **c**, Kaplan–Meier curves for CD4^+^ naive T cells on C1D1 with Gehan–Breslow *P* values. **d**, Volcano plot for MFS based on C3D1 peripheral blood CyTOF data (*n* = 65; patients without samples or outcome data were excluded) showing log-rank test (*y* axis) and Cox regression hazard ratio (*x* axis). **e**, Kaplan–Meier curves with Gehan–Breslow *P* values for CD8^+^ naive T cells at C3D1. **f**, Volcano plot showing the differential expression of peripheral blood proteins on C1D1 (*n* = 71; patients without samples or outcome data were excluded) according to cCR status (−log_10_(*P* value) in *x* axis and log_2_ fold change calculated using moderated t-statistic). **g**, Volcano plot showing the differential expression of peripheral blood proteins on C3D1 (*n* = 65; patients without samples or outcome data were excluded) according to cCR status (−log_10_(*P* value) in *x* axis and log_2_ fold change calculated using moderated t-statistic). **h**, Heat map showing the normalized expression (*z*-score) for peripheral blood proteins increased on C3D1 according to cCR status (hierarchically clustered using Ward’s algorithm). **i**, Volcano plot for MFS based on C1D1 expression of proteins showing log-rank test (*y* axis) and Cox regression hazard ratio (*x* axis). **j**, Kaplan–Meier curves for IL6 expression at C1D1 with Gehan–Breslow *P* values. **k**, Volcano plot for MFS based on C3D1 protein expression showing log-rank test (*y* axis) and Cox regression hazard ratio (*x* axis). **l**, Kaplan–Meier curves for ANGPT2 expression at C3D1 with Gehan–Breslow *P* values. **m**, Volcano plot for MFS based on urine proteins at time of clinical restaging (*n* = 59, patients without samples or outcome data were excluded) showing log-rank test (*y* axis) and Cox regression hazard ratio (*x* axis). For associations with cCR, adjustment for multiple comparisons was performed using the Benjamini–Hochberg method. **a** was created with BioRender. FDR, false discovery rate; HR, hazard ratio; Nivo, nivolumab; NA, not applicable.

**Extended Data Fig. 3:**
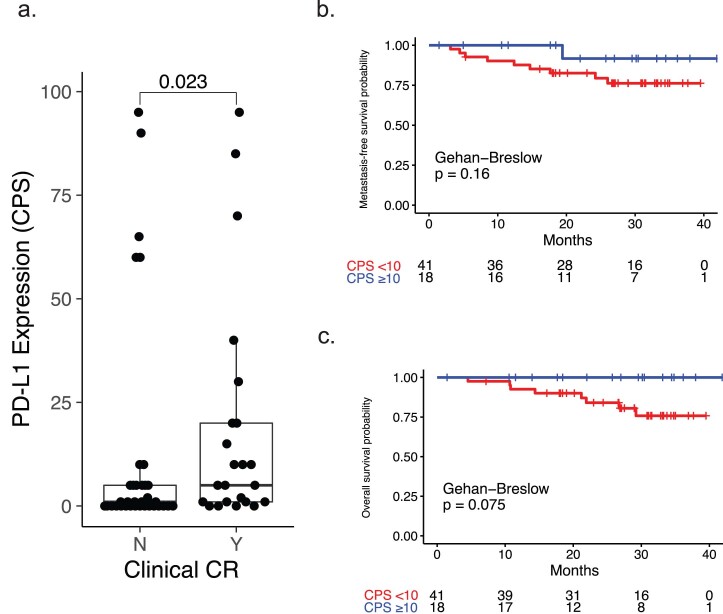
Relationship between PD-L1 immunohistochemical staining of pre-treatment transurethral resection of bladder tumor specimens (n = 59) and clinical outcomes. (**a**) Relationship between PD-L1 immunohistochemical staining and scoring according to the combined positive score (CPS) and achievement of a clinical complete response (Y, yes; N, no) in 59 patients with available samples for testing. Box and whisker plots demonstrating CPS for patients achieving a clinical complete response (min 0, max 95, median 5, 1^st^ quartile 1, 3^rd^ quartile 20) and not achieving a clinical complete response (min 0, max 95, median 1, 1^st^ quartile 0, 3^rd^ quartile 5). (**b**) Kaplan–Meier curve for metastasis-free survival according to PD-L1 expression of baseline TURBT specimens using the cut-point of CPS ≥ 10 versus < 10. (**c**) Kaplan–Meier curve for overall survival according to PD-L1 expression of baseline TURBT specimens using the cut-point of CPS ≥ 10 versus < 10.

**Extended Data Fig. 4:**
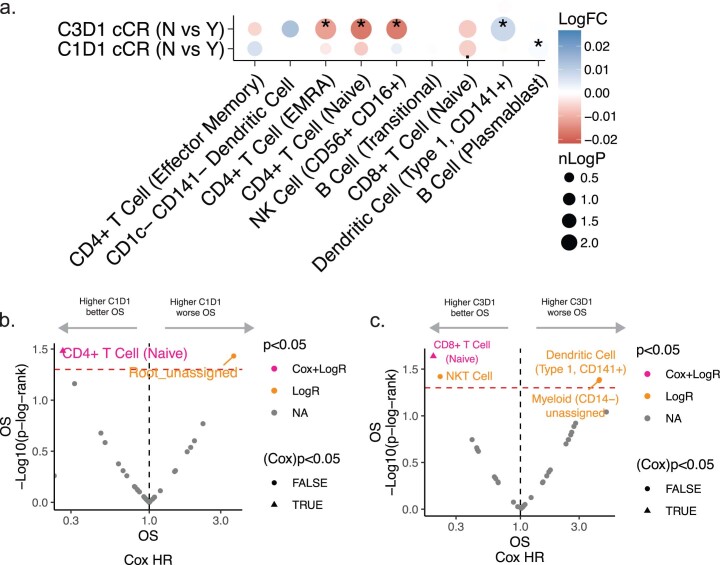
Peripheral blood mass cytometry (CyTOF) and association with response and overall survival. (**a**) Heatmap, showing the differential abundance results between cell populations and association with clinical complete response (cCR). The x-axis shows the cell type annotation from ASTROLABE. The y-axis shows the timepoint of the comparison between responders (Y) and non-responders (N). The direction of the logFC is dictated by the comparison. Negative logFC indicates increase of expression in responders. The size of the circle indicates the significance. The larger the circle the smaller the p value. Additionally, the stars on top of the circles indicate p < 0.05 significance. Circles without stars indicate p > 0.05 or non-statistically significant. (**b**) Peripheral blood CyTOF data. Volcano plot for overall survival (OS) based on cycle 1 day 1 (C1D1) abundances of cell populations showing log rank test (y-axis) and Cox regression hazard ratio (x-axis). Cell abundances significant for both tests are shown in pink. A lower hazard ratio (left side of the black line) is associated with reduced risk. (**c**) Peripheral blood CyTOF data. Volcano plot for OS based on cycle 3 day 1 (C3D1) abundances of cell populations showing log rank test (y-axis) and Cox regression hazard ratio (x-axis). Cell abundances significant for both tests are shown in pink. A lower hazard ratio (left side of the black line) is associated with reduced risk.

To determine whether baseline and/or on-treatment immune parameters were associated with achieving a cCR or with metastasis-free survival or overall survival, additional exploratory analyses were pursued. PD-L1 immunohistochemical staining (22C3 antibody clone) of baseline TURBT specimens was completed in a central laboratory. A higher PD-L1 combined positive score was associated with a higher cCR rate, although the relationship between higher PD-L1 expression and longer metastasis-free survival or overall survival did not achieve statistical significance (Extended Data Fig. 3a–c). Mass cytometry (CyTOF) was performed on peripheral blood mononuclear cells (PBMCs) to define frequency of immune subsets, and a panel of 92 soluble protein analytes was measured in the plasma (Olink) on cycle 1, day 1 and cycle 3, day 1 (Fig. 4a). Protein analytes were also measured in the urine at the time of post-cycle-4 clinical restaging. Although the abundance of specific immune cell populations on cycle 1, day 1 and cycle 3, day 1 correlated with achieving a cCR versus not, such findings did not achieve statistical significance after correction for false discovery (Extended Data Fig. 4a). A higher abundance of cycle 1, day 1 naive CD4^+^ T cells in peripheral blood was associated with significantly longer metastasis-free survival and overall survival (Fig. 4b,c and Extended Data Fig. 4b). On landmark analysis, a higher abundance of circulating naive CD8 T cells on cycle 3, day 1 was associated with significantly longer metastasis-free survival and overall survival (Fig. 4d,e and Extended Data Fig. 4c).

**Extended Data Fig. 5:**
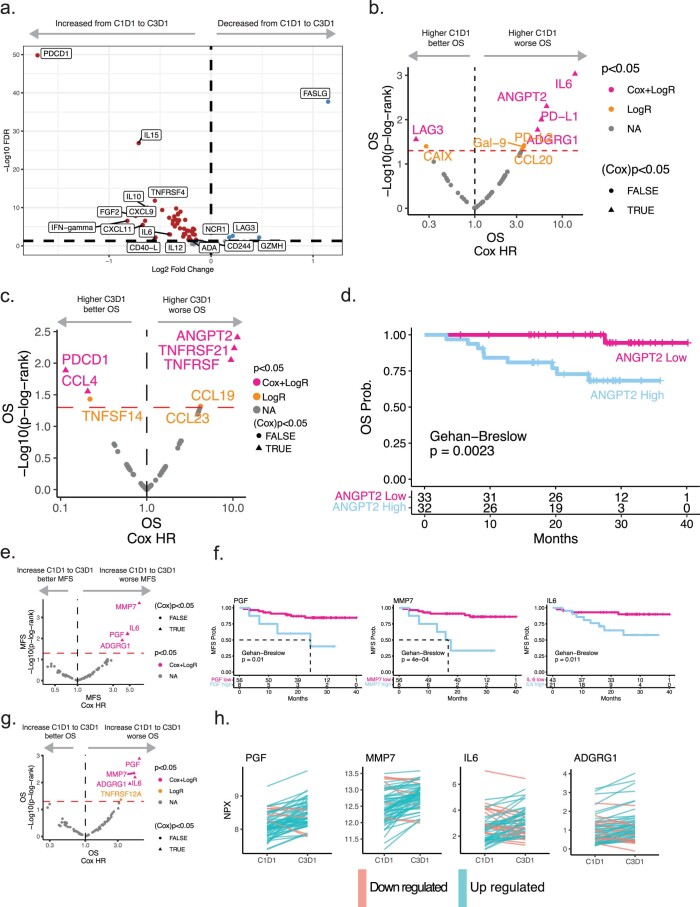
Peripheral blood protein analytes and association with response and overall survival. (**a**) Volcano plot demonstrating trend in peripheral blood analytes by Olink from cycle 1 day 1 (C1D1) to cycle 3 day 1 (C3D1) demonstrating largest increase in PD-1 (PDCD1). (**b**) Peripheral blood protein analyte data. Volcano plot for overall survival (OS) based on C1D1 levels of protein analytes showing log rank test (y-axis) and Cox regression hazard ratio (x-axis). Analytes significant for both tests are shown in pink. A lower hazard ratio (left side of the black line) is associated with reduced risk. (**c**) Peripheral blood protein analyte data. Volcano plot for overall survival (OS) based on C3D1 levels of protein analytes showing log rank test (y-axis) and Cox regression hazard ratio (x-axis). Analytes significant for both tests are shown in pink. A lower hazard ratio (left side of the black line) is associated with reduced risk. (**d**) Kaplan-Meier curves showing better OS as measured from C3D1 in patients with lower versus higher peripheral blood levels of angiopoietin-2 (ANGPT2). (**e**) Peripheral blood protein analyte data. Volcano plot for metastasis-free survival (MFS) based on changes in levels of protein analytes from C1D1 to C3D1 showing log rank test (y-axis) and Cox regression hazard ratio (x-axis). Analytes significant for both tests are shown in pink. A lower hazard ratio (left side of the black line) is associated with reduced risk. (**f**) Kaplan-Meier curves for the proteins in E showing MFS for patients with an increase in PGF, MMP7, or IL6 from C1D1 to C3D1 versus a decrease in levels of these proteins. (**g**) Peripheral blood protein analyte data. Volcano plot for OS based on changes in levels of protein analytes from C1D1 to C3D1 showing log rank test (y-axis) and Cox regression hazard ratio (x-axis). Analytes significant for both tests are shown in pink. A lower hazard ratio (left side of the black line) is associated with reduced risk. (**h**) Peripheral blood protein analyte data. Line plots showing individual changes in protein levels from C1D1 to C3D1. N = 64 patients for each analyte for C1D1 to C3D1 comparisons.

**Extended Data Fig. 6:**
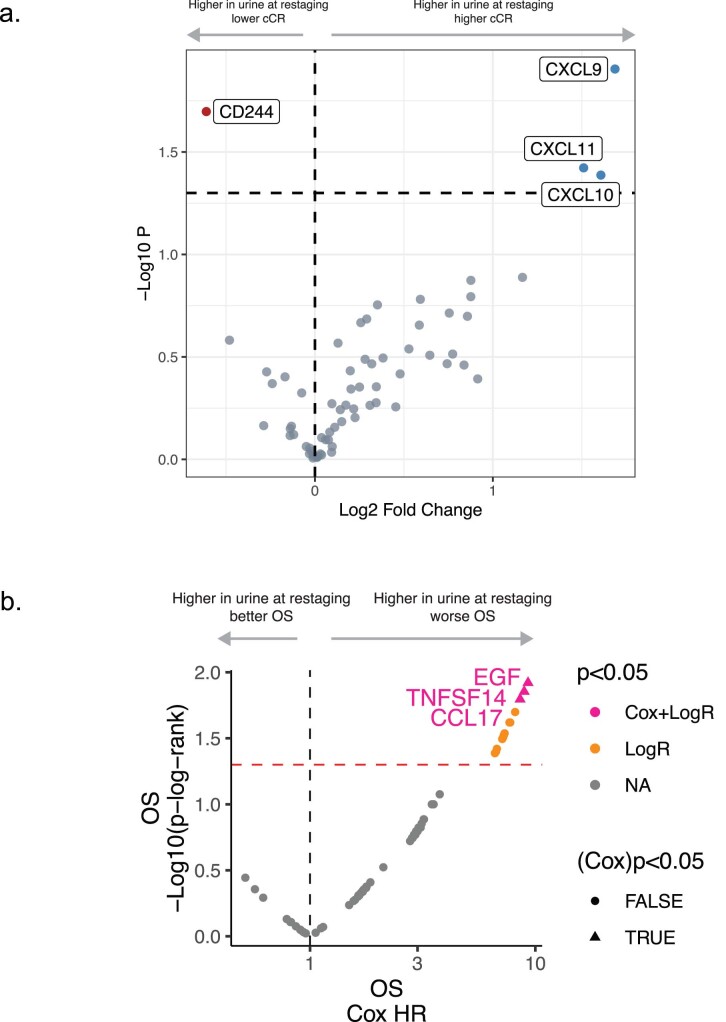
Urine protein analytes and association with response and survival. (**a**) Urine Olink protein analyte data. Volcano plot showing the differential expression at time of restaging among patients achieving or not achieving a clinical complete response. The statistical significance or -log10(Pval) is shown on y-axis and log2 protein levels is shown on x-axis. (**b**) Urine protein analyte data. Volcano plot for overall survival (OS) based levels of protein analytes in the urine at the time of restaging showing log rank test (y-axis) and Cox regression hazard ratio (x-axis). Analytes significant for both tests are shown in pink. A lower hazard ratio (left side of the black line) is associated with reduced risk.

Several plasma protein analytes significantly increased on treatment from cycle 1, day 1 to cycle 3, day 1 (Extended Data Fig. 5a). Cycle 1, day 1 levels of plasma analytes were not significantly associated with cCR after correction for false discovery (Fig. 4f). However, cycle 3, day 1 plasma levels of TNF-related apoptosis-inducing ligand (TRAIL), FasL and CD244 were significantly higher in patients achieving a cCR versus not (Fig. 4g,h). Higher cycle 1, day 1 plasma levels of several analytes were associated with significantly shorter metastasis-free and overall survival, including IL6 and angiopoietin-2 (ANGPT2) (Fig. 4i,j and Extended Data Fig. 5b,c). On landmark analysis at cycle 3, day 1, similar associations with metastasis-free survival and overall survival were observed for several plasma analytes, such as IL6 and ANGPT2, based on either cycle 3, day 1 levels or on-treatment changes in levels from cycle 1, day 1 to cycle 3, day 1 (Fig. 4k,l and Extended Data Fig. 5d–h). No urine analytes from samples obtained at the time of clinical restaging were significantly associated with achieving a cCR after correction for false discovery (Extended Data Fig. 6a), whereas increased urine levels of analytes, such as epidermal growth factor (EGF), at the clinical restaging timepoint were associated with both significantly inferior metastasis-free survival and overall survival (Fig. 4m and Extended Data Fig. 6b).

## Discussion

To our knowledge, this is among the first prospective trials to test TURBT plus cisplatin-based chemotherapy as definitive bladder-sparing treatment for MIBC; the first to define the performance characteristics of uniformly assessed and defined cCR as a tool for patient selection for this strategy; and the first to integrate immune checkpoint blockade into this approach. Our study demonstrates that stringently defined cCR is associated with favorable survival outcomes and that prolonged bladder-intact survival is achievable in a large subset of patients with MIBC and a cCR to TURBT and gemcitabine, cisplatin, plus nivolumab.

Radical cystectomy or radiation therapy are mainstays of local treatment for MIBC. However, despite such treatments, more than 50% of patients experience metastatic recurrence^2,26^. Radical cystectomy requires urinary diversion and is associated with a non-negligible risk of morbidity and mortality^1^. Concurrent chemoradiation is associated with an apprioximately 17% risk of late grade ≥2 pelvic toxicity, and approximately one-third of patients report worsening quality of life 6 months after completing treatment and persisting on long-term follow-up^27,28^. Furthermore, salvage cystectomy due to local recurrence is required in 12–19% of patients treated with radiation with or without concurrent chemotherapy^29^. Systemic therapy is associated with a different constellation of potential adverse events, and gemcitabine, cisplatin, plus PD-1 blockade demonstrated a toxicity profile consistent with other studies^13,14^. Each of these treatment modalities is an important component of optimal treatment of MIBC, and each is associated with specific tradeoffs. Risk-adapted MIBC treatment paradigms that balance both efficacy and survivorship while also reducing treatment-related burden could represent an important addition to patient-centered care.

Although the IMvigor 130 and Keynote 361 studies exploring concurrent administration of platinum-based chemotherapy and PD-1/PD-L1 blockade in patients with metastatic bladder cancer did not demonstrate improvements in survival, those studies pooled patients treated with cisplatin-based and carboplatin-based chemotherapy^30,31^. Cisplatin may induce distinct immunomodulatory effects and combine more favorably with immune checkpoint blockade^32^. Consistent with this hypothesis, CheckMate 901, exploring gemcitabine, cisplatin, plus nivolumab versus gemcitabine plus cisplatin in patients with metastatic bladder cancer, did demonstrate an improvement in progression-free and overall survival with the immunotherapy combination (https://news.bms.com/news/corporate-financial/2023/Opdivo-nivolumab-in-Combination-with-Cisplatin-Based-Chemotherapy-Shows-Overall-Survival-and-Progression-Free-Survival-Benefit-for-Cisplatin-Eligible-Patients-with-Unresectable-or-Metastatic-Urothelial-Carcinoma-in-the-Phase-3-CheckMate--901-Trial/default.aspx). Sequential chemotherapy followed by switch maintenance immune checkpoint blockade has also demonstrated improved progression-free and overall survival in metastatic bladder cancer and has become a standard of care^33,34^. The contribution of concurrent versus sequential nivolumab to the favorable outcomes observed in our study cannot be fully delineated.

Our study has potential limitations. The median follow-up of patients achieving a cCR was 30 months at the time of the data lock. The vast majority of local and distant recurrences occur within 2 years of treatment in previous bladder-sparing studies of MIBC, although whether the same pattern and timing holds true for patients not undergoing cystectomy or receiving radiation is not well established^2,26^. Therefore, longer-term follow-up data are needed to fully understand the impact of this treatment regimen on disease control. The need for later cystectomy in a subset of patients developing local recurrence after a cCR raises the question of whether all patients achieving a cCR should receive (chemo)radiation to further optimize the likelihood of bladder preservation. The complex interplay of issues related to organ preservation, cancer control and potential over-treatment with such an approach warrants further consideration and investigation. Patient-reported outcome data would provide important additional context, but such information was not collected in our study.

A disconnect between clinical and pathological staging has often been cited as a barrier to TURBT plus systemic therapy as definitive treatment for MIBC, although many analyses highlighting such a disconnect have been retrospective and without uniform approaches to clinical response assessment^35,36^. Notwithstanding, a focus solely on the discrepancies between clinical and pathological staging may undermine the possibility that cystectomy at the time of local recurrence can achieve similar survival to immediate cystectomy in the subset of patients with subclinical disease not detected at initial clinical restaging. Many cCRs in patients who later develop local recurrence may indeed represent ‘major pathological responses’ accompanied by a distinct tumor biology and prognosis; the relatively favorable outcomes observed in our patients achieving a cCR and undergoing cystectomy for local recurrence is supportive of this notion. That cCRs do not align completely with pCRs is unlikely unique to bladder cancer. Even in locally advanced mismatch repair protein deficient (dMMR) colorectal tumors that are highly sensitive to immunotherapy, early data indicate a 100% cCR rate with immune checkpoint blockade in a small cohort of patients with dMMR rectal cancer deferring definitive surgery or chemoradiation, whereas a 67% pCR rate was observed with neoadjuvant immune checkpoint blockade followed by colectomy in dMMR colon cancer^37,38^. As defined in our study, cCR was associated with favorable bladder-intact and overall survival outcomes.

Integrating pre-treatment and on-treatment biomarkers could potentially refine selection of patients achieving a cCR after TURBT plus systemic therapy for omission of additional local therapy. Mutations in a pre-specified set of genes selected based on previous work^15–22^ did not clearly enhance the ability of cCR to identify patients achieving prolonged bladder-intact survival. Our analysis is limited by the potential limitations of tumor-only DNA sequencing, the sample size and the paucity of pathogenic alterations in some genes (for example, *ATM*), although other studies have also been unable to confirm the relationship between these molecular alterations and clinical outcomes in patients with MIBC^36,39^. Ongoing clinical trials are prospectively assessing the role of such molecular alterations in selecting patients for definitive treatment with TURBT plus chemotherapy (NCT02710734 and NCT03609216). Although VI-RADS^25^, a standardized approach to bladder cancer MRI imaging and reporting, was developed for initial bladder cancer staging, our data highlight the prognostic impact of this system after systemic therapy for MIBC and the need for further study as a tool for selection of patients for bladder-sparing approaches in future trials.

Analysis of circulating immune parameters may facilitate biomarker discovery and insights related to the immunomodulatory effects of treatment. Mass cytometry analysis of PBMCs revealed that a higher abundance of pre-treatment naive CD4 T cells and on-treatment naive CD8 T cells was associated with longer metastasis-free and overall survival. Multiplex proteomic analysis of plasma revealed that increased on-treatment levels of cytotoxicity-related markers TRAIL, FasL and CD244 were associated with a higher likelihood of achieving a cCR. TRAIL and FasL are members of the tumor necrosis factor (TNF) superfamily and are expressed by immune effector cells, whereas CD244 is a surface receptor on natural killer (NK) cells and a subset of CD8 T cells^40^. Higher pre-treatment and on-treatment IL6 and ANGPT2 levels were associated with worse survival outcomes consistent with previous clinical and preclinical studies^41,42^. Overall, these findings are suggestive of a more robust NK cell and CD8 T cell immune response in patients with a more favorable response to treatment and underlying tumor-promoting inflammation in patients experiencing worse outcomes. The mechanisms by which on-treatment augmentation of immunity is achieved are currently being further explored, leveraging pre-treatment and post-treatment tumor tissue.

In our study, neoadjuvant gemcitabine, cisplatin, plus nivolumab after TURBT was associated with a cCR rate of 43%, and clinical response assessment identified patients with particularly favorable outcomes and facilitated bladder sparing. Genomic, imaging and immunological biomarkers have the potential to refine this treatment paradigm, but they require further investigation. These findings may help advance a more personalized approach to the management of MIBC.

## Methods

### Study design

HCRN GU 16–257 is phase 2, investigator-initiated, multicenter clinical trial. Cisplatin-eligible patients with MIBC enrolled at seven medical centers received treatment with gemcitabine, cisplatin, plus nivolumab (Fig. 1a). Clinical restaging was performed after cycle 4. Patients achieving a cCR were offered the option to proceed with radical cystectomy versus retain their bladder and receive eight additional doses of nivolumab followed by surveillance. Patients not achieving a cCR were recommended to proceed with radical cystectomy. The surveillance schedule is outlined in the protocol (Supplementary Information). The study was conducted in accordance with the Declaration of Helsinki. The protocol was approved by local ethics committees at the Icahn School of Medicine at Mount Sinai, the City of Hope Comprehensive Cancer Center, the Huntsman Cancer Institute University of Utah, the Oregon Health and Science University, the Penn Medicine Abramson Cancer Center, the Rutgers Cancer Institute of New Jersey, the University of Southern California and the University of Wisconsin, and written informed consent was provided by all patients before enrollment. The trial was registered at ClinicalTrials.gov (NCT03558087).

### Patients

Inclusion criteria:

- Written informed consent and HIPAA authorization for release of personal health information before registration
- Age ≥18 years at the time of consent
- Eastern Cooperative Oncology Group (ECOG) performance status of ≤1 within 28 d before registration
- Histological evidence of clinically localized muscle-invasive UC of the bladder (that is, cT2N0M0)
- Candidate for cystectomy as per treating physician
- Adequate organ function
- Adequate archival tissue identified at screening (that is, at least 15 unstained slides or paraffin block)
- Women of childbearing potential must have a negative serum or urine pregnancy test within 7 d before cycle 1, day 1.

Exclusion criteria:

- Prior treatment with systemic chemotherapy for muscle-invasive UC of the bladder
- Active infection requiring systemic therapy
- Pregnant or breastfeeding
- Any serious or uncontrolled medical disorder that, in the opinion of the investigator, may increase the risk associated with study participation or study drug administration, impair the ability of the subject to receive protocol therapy or interfere with the interpretation of study results
- Prior malignancy active within the previous 3 years except for locally curable cancers that have been apparently cured
- Subjects with active, known or suspected autoimmune disease. Subjects with vitiligo, type I diabetes mellitus, residual hypothyroidism due to autoimmune condition requiring only hormone replacement, psoriasis not requiring systemic treatment or conditions not expected to recur in the absence of an external trigger are permitted to enroll.
- Subjects with a condition requiring systemic treatment with either corticosteroids (>10 mg daily prednisone equivalents) or other immunosuppressive medications within 14 d of study drug administration. Inhaled or topical steroids and adrenal replacement doses >10 mg daily prednisone equivalents are permitted in the absence of active autoimmune disease.
- Prior treatment with an anti-PD-1, anti-PD-L1, anti-PD-L2, anti-CTLA-4 antibody or any other antibody or drug specifically targeting T cell co-stimulation or immune checkpoint pathways
- Grade ≥2 neuropathy (National Cancer Institute Common Terminology Criteria for Adverse Events (NCI CTCAE) version 4.03)
- Prior radiation therapy for bladder cancer
- Positive test for hepatitis B virus surface antigen (HBV sAg) or hepatitis C virus RNA or hepatitis C antibody (HCV antibody), indicating acute or chronic infection
- Known history of testing positive for HIV or known AIDS
- Evidence of interstitial lung disease or active, non-infectious pneumonitis

### Treatment

Cycles 1–4 of treatment included gemcitabine 1,000 mg m^−^^2^ on days 1 and 8, cisplatin 70 mg m^−^^2^ on day 1 and nivolumab 360 mg on day 1, all administered intravenously in 21-d cycles. Patients achieving a cCR and opting to proceed without cystectomy received single-agent nivolumab 240 mg intravenously every 2 weeks for eight doses. Patients with a cCR and forgoing immediate cystectomy then proceeded with surveillance using the following strategy: urine cytology every 3 months for years 1–2, every 6 months for years 2–4 and annually for year 5; cystoscopy every 3 months for years 1–2, every 6 months for years 2–4 and annually for year 5; and cross-sectional imaging of the chest, abdomen and pelvis every 3 months to year 1.5, every 6 months to year 3 and annually to year 5. Patients with an invasive local recurrence were recommended to proceed with cystectomy.

Adverse events were graded according to the NCI CTCAE version 4.03. Adverse events were managed according to algorithms based on the specific toxicity as defined in the protocol.

### Clinical restaging and cCR definition

After cycle 4 of gemcitabine, cisplatin, plus nivolumab, patients underwent clinical restaging including MRI of the abdomen and pelvis or CT if MRI was contraindicated and CT of the chest, rigid cystoscopy with biopsies and urine cytology. Transurethral resection of any visible tumor and/or the prior tumor site was performed. In addition, biopsies were obtained from the following sites: trigone, left, right, anterior, posterior and dome. In men, prostatic urethral biopsies were performed. A cCR was defined as meeting all of the following: (1) no evidence of malignancy on biopsy with the exception of low-grade papillary (Ta) tumors; (2) no malignant cells on urine cytology; and (3) no evidence of local or metastatic disease on cross-sectional imaging. Residual bladder wall changes on cross-sectional imaging were interpreted by the treating investigator in consultation with the local radiologist and in the context of the bladder biopsy results. A blinded post hoc central review of the restaging MRI scans was completed by two study radiologists (S.L. and B.E.A.) to assign a VI-RADS^25^ score—a standardized approach to bladder cancer MRI assessment and reporting. Inter-rater agreement was assessed using the weighted kappa statistic^43^. The VI-RADS value from the more experienced reviewer (S.L.) was used when there was not agreement.

### PD-L1 immunohistochemistry on baseline TURBT specimens

Immunohistochemistry for PD-L1 was performed in the Department of Pathology at the Mount Sinai Hospital using the 22C3 antibody clone. PD-L1 expression was quantified by a single genitourinary pathologist (G.K.H.) blinded to clinical outcome data using the combined positive score (CPS), defined as the percentage of PD-L1-expressing tumor and infiltrating immune cells relative to the total number of tumor cells. A cutpoint of CPS ≥ 10 was used to define ‘high’ PD-L1 expression as per previous studies in UC^44^.

### DNA sequencing of baseline TURBT specimens

Archival baseline TURBT tissue underwent tumor-only targeted DNA sequencing using the Illumina NextSeq platform (Caris Life Sciences). An Agilent custom-designed SureSelect XT assay (Caris MI TumorSeek 592-Gene NGS Panel) was used to enrich 591 whole-gene targets. Sequencing and gene variant calling were carried out as previously described; the pipeline automatically filters out known common germline population variants (that is, from databases such as dbSNP) and flags pathogenic mutations that are potentially germline^45^. To address artifacts that might be introduced in formalin-fixed, paraffin-embedded (FFPE) samples, samples with low depth or unusual variant composition are flagged for review and potential resequencing. Multiple non-reference reads (>20) were needed to support variant calling. In addition, if forward and reverse reads at the single-nucleotide polymorphism (SNP) locations largely deviated from balance, the variants were filtered out. For the flanking regions around the SNPs in the genes in Fig. 3, the mean sequencing depth was 1,243 (137–6,407). Mutations were considered pathogenic or presumed pathogenic according to guidelines set by the American College of Medical Genetics and the Association for Molecular Pathology^46^. Mutations in *ERCC2* were further annotated incorporating the results of published functional assays (K.M.)^47^. TMB was calculated using only missense mutations as previously described^48^.

### Mass cytometry (CyTOF)

Mass cytometry (CyTOF) was performed on PBMCs obtained on cycle 1, day 1 and cycle 3, day 1 of treatment. PBMCs were stained with the CyTOF antibody panel detailed in Supplementary Table 5. All antibodies were either purchased pre-conjugated from Fluidigm or conjugated in-house (using commercial X8 polymer conjugation kits purchased from Fluidigm) at the Human Immune Monitoring Center (HIMC), Icahn School of Medicine at Mount Sinai. All in-house conjugated antibodies were titrated and validated on healthy donor PBMCs. For longitudinal monitoring of phenotypic changes, cells from selected timepoints were thawed, counted and assessed for viability using the Nexcelom Cellaca Automated Cell Counter (Nexcelom Bioscience) along with acridine orange/propidium iodine staining (Nexcelom Bioscience). For sample timepoint batching, live-cell CyTOF barcoding was performed using anti-B2M antibodies conjugated to unique cadmium isotopes. Rhodium-103 viability and Human TruStain FcX staining were performed simultaneously at room temperature for 30 min. After cell washing in flow cytometry buffer (1× PBS + 0.2% BSA + 0.05% NaN_3_), cells were stained with a cocktail of surface antibodies (Supplementary Table 5). Surface-stained cells were further fixed with 1.6% formaldehyde. Each sample was then barcoded with the CyTOF Cell-ID 20-Plex Palladium Barcoding Kit (Fluidigm), pooled and fixed in freshly made 4% paraformaldehyde containing 125 nM intercalator-Ir (Fluidgm) and 300 nM OsO_4_ (Acros Organics) and stored at −80 °C in FBS + 10% DMSO. Samples were washed with cell staining buffer (Fluidigm) and re-suspended in CAS buffer containing EQ normalization beads (Fluidigm) and acquired on a Helios mass cytometer equipped with a wide-bore sample injector at an event rate of <400 events per second. After acquisition, repeat acquisitions of the same sample were concatenated and normalized using Fluidigm software. The FCS file was further cleaned using the HIMC internal pipeline. The pipeline removed any aberrant acquisition time windows of 3 s where the cell sampling event rate was too high or too low (2 s.d. from the mean). EQ normalization beads spiked into every acquisition and used for normalization were removed, along with events that had low DNA signal intensity. The pipeline also was used to demultiplex the cleaned and pooled FCS files into constituent single-sample files. The cosine similarity of every cell’s Pd barcoding channels to every possible barcode used in a batch was calculated and then was assigned to its highest similarity barcode. Once the cell had been assigned to a sample barcode, the difference between its highest and second highest similarity scores was calculated and used as a signal-to-noise metric. Any cells with low signal to noise were flagged as multiplets and removed from that sample. Finally, acquisition multiplets were removed based on the Gaussian parameters Residual and Offset acquired by the Helios mass cytometer.

Astrolabe was employed for automated computational annotation (Astrolabe Diagnostics). CyTOF analysis was performed using Astrolabe annotated data and statistical modeling with R. The data were loaded into R using the package ‘orloj’. Astrolabe gating strategies were manually reviewed for a subset of samples. Data were uploaded to Cytobank for quality control analysis and visualization.

### Multiplex protein immunoassay

Plasma (cycle 1, day 1 and cycle 3, day 1) and urine (at time of restaging) were analyzed using the Olink Immuno-Oncology panel, which measures 92 proteins involved in immune response and tumor biology, using the Olink multiplex assay (Olink Bioscience) according to the manufacturer’s instructions. The Olink panel uses proximity extension assay technology, which relies on pairs of DNA-labeled antibodies that bind to target proteins and generate unique reporter molecules that can be quantified by real-time polymerase chain reaction. The Olink panel provides normalized protein expression units (NPX), which are log_2_-transformed values proportional to protein concentration. One NPX difference is equal to a doubling of the protein concentration.

### Statistical analysis

The co-primary objectives of the study were to (1) estimate the cCR rate with gemcitabine, cisplatin, plus nivolumab and (2) assess the positive predictive value of cCR for a composite outcome measure of (1) 2-year metastasis-free survival in patients achieving a cCR and opting to not undergo immediate cystectomy or (2) <pT1N0 in patients with a cCR who opted for immediate cystectomy. Secondary objectives included assessing the association between genomic alterations in a pre-specified panel of genes detected in pre-treatment TURBT tissue (*ERCC2*, *ATM*, *RB1* and *FANCC*^15–22^) as well as TMB (using an established cutpoint of ≥10 mut/Mb^23,24^) and clinical outcomes. Additional secondary objectives included safety, metastasis-free survival, overall survival and bladder-intact survival.

The sample size was based on the following assumptions: (1) patients without a cCR would not be suitable to forgo cystectomy; (2) ~40% of enrolled patients would achieve a cCR; and (3) ~35% of enrolled patients would achieve the composite outcome measure. Therefore, our assumption implied that the negative predictive value of a cCR would be 1. The sample size was based on the CI width of the positive predictive value of cCR for the composite outcome measure and generated such that the lower bound of the 95% one-sided CI exceeded 80%. This required enrollment of 68 patients, and the sample size was increased to 76 to account for potential missing data.

Rates were calculated using percentages and compared among different groups using Fisher’s exact test. Time-to-event outcomes were analyzed using the Kaplan–Meier method and log-rank test. When comparing time-to-event outcomes for restaging cCR status and restaging VI-RADS, landmark analyses were conducted using the restaging times as the landmark time (that is, time 0). *P* values less than 0.05 were deemed statistically significant.

For analysis of multiplex protein immunoassay (Olink) data, the data were normalized with the reference samples using R software. The data distribution per sample was compared, and samples were inspected with warnings after NPX conversion. For CyTOF analysis, the data were normalized to percent of cell abundance and 95th percentile of surface protein expression. For Olink and CyTOF, differential protein expression and differential cell abundance, respectively, were calculated using a mixed effect linear model strategy to adjust for relevant clinical variables (ECOG performance status) and demographics (age, race and gender). First, the variance profiles and data distributions were explored to identify potential biases and assess the effect of relevant covariates in the analysis using the packages lme4, variancePartition and Dream. For Olink and CyTOF, quality control analysis served to identify biases such as low detection and poor sample quality. The filters included removing variables with more than 40–70% not available or under the limit of detection values. The variables were verified as linearly independent such that there was no redundancy in the data. After quality control, individual expression or abundance was modeled as a function of relevant endpoints and covariates. Differential expression or abundance analyses were performed applying a contrast matrix to each regression model (one per endpoint) and using the moderated t-statistic or log odds when appropriate. False discovery rate adjustment was performed on resulting *P* values for multiple testing as described by Benjamini and Hochberg^49^. The Kaplan–Meier method was used to estimate metastasis-free and overall survival. Comparisons of time-to-event distributions between groups were made with the log-rank and Gehan–Breslow tests. Univariable Cox proportional hazard regression models were used to estimate the hazard ratios and corresponding 95% CIs for metastasis-free and overall survival. Landmark analyses were employed for cycle 3, day 1 or restaging timepoints. All statistical analyses were performed using SAS software version 9.4 (SAS Institute) and RStudio version 4.0.0 (R Core Team).

### Reporting summary

Further information on research design is available in the Nature Portfolio Reporting Summary linked to this article.

## Online content

Any methods, additional references, Nature Portfolio reporting summaries, source data, extended data, supplementary information, acknowledgements, peer review information; details of author contributions and competing interests; and statements of data and code availability are available at 10.1038/s41591-023-02568-1.

### Supplementary information

Supplementary Information Supplementary Tables 1–5.

Reporting Summary

Supplementary Data Clinical Trial Protocol.

## Figures and Tables

**Extended Data Table 1:**
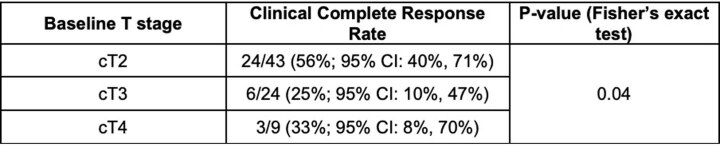
Association between baseline clinical stage and cCR rate

**Extended Data Table 2:**
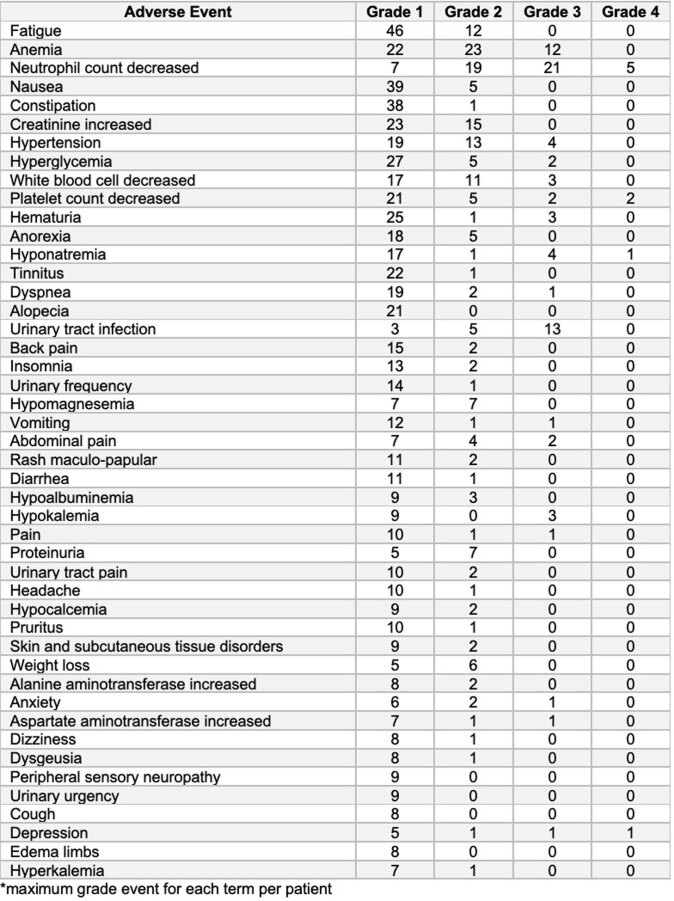
Number of treatment-emergent grade ≥3 adverse events occurring in ≥10% patient(s)* (*n* = 76 patients) per NCI CTCAE version 4.03

**Extended Data Table 3:**
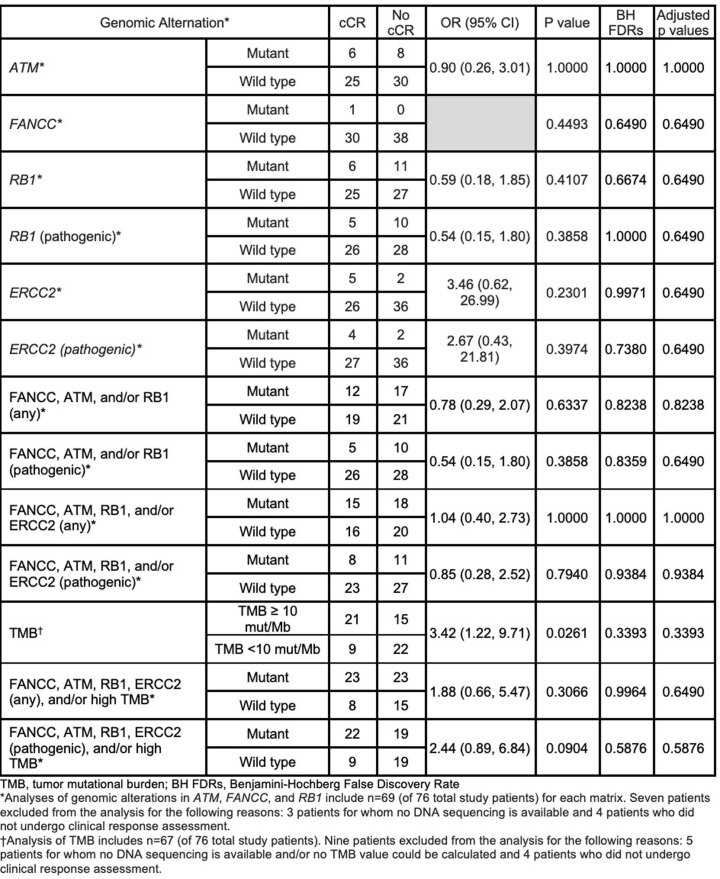
Association between pre-specified genomic alterations in pre-treatment transurethral resection of bladder tumor tissue and likelihood of achieving a cCR. *P* values are based on Fisher’s exact test

## Data Availability

In accordance with NIH’s Genomic Data Sharing Policy, the DNA sequencing data used to support the findings of this study have been deposited under controlled access in the database of Genotypes and Phenotypes (dbGaP) under accession number phs0003372. Genomic, clinical, mass cytometry and protein analyte data from this study used to support this publication will be made available upon reasonable request from a qualified medical or scientific professional for the specific purpose laid out in that request and may include de-identified individual participant data. Requests for secondary use of this data will require completing a data use agreement (https://osp.od.nih.gov/wp-content/uploads/Model_DUC.pdf) and submitting a data access request to the NIH.
