# Author Correction: Gemcitabine and cisplatin plus nivolumab as organ-sparing treatment for muscle-invasive bladder cancer: a phase 2 trial

**DOI:** 10.1038/s41591-024-02814-0

**Published:** 2024-01-19

**Authors:** Matthew D. Galsky, Siamak Daneshmand, Sudeh Izadmehr, Edgar Gonzalez-Kozlova, Kevin G. Chan, Sara Lewis, Bassam El Achkar, Tanya B. Dorff, Jeremy Paul Cetnar, Brock O. Neil, Anishka D’Souza, Ronac Mamtani, Christos Kyriakopoulos, Tomi Jun, Mahalya Gogerly-Moragoda, Rachel Brody, Hui Xie, Kai Nie, Geoffrey Kelly, Amir Horowitz, Yayoi Kinoshita, Ethan Ellis, Yohei Nose, Giorgio Ioannou, Rafael Cabal, Diane M. Del Valle, G. Kenneth Haines, Li Wang, Kent W. Mouw, Robert M. Samstein, Reza Mehrazin, Nina Bhardwaj, Menggang Yu, Qianqian Zhao, Seunghee Kim-Schulze, Robert Sebra, Jun Zhu, Sacha Gnjatic, John Sfakianos, Sumanta K. Pal

**Affiliations:** 1 Division of Hematology and Medical Oncology, Icahn School of Medicine at Mount Sinai, New York, NY USA; 2 Tisch Cancer Institute, Icahn School of Medicine at Mount Sinai, New York, NY USA; 3 Department of Urology, Keck School of Medicine of USC, Norris Comprehensive Cancer Center, Los Angeles, CA USA; 4 Department of Oncological Sciences, Tisch Cancer Institute, Icahn School of Medicine at Mount Sinai, New York, NY USA; 5 Department of Urology, City of Hope Comprehensive Cancer Center, Duarte, CA USA; 6 Department of Radiology, Tisch Cancer Institute, Icahn School of Medicine at Mount Sinai, New York, NY USA; 7 Department of Medical Oncology & Therapeutics, City of Hope Comprehensive Cancer Center, Duarte, CA USA; 8 Division of Hematology and Medical Oncology, Oregon Health and Science University, Portland, OR USA; 9 Department of Urology, University of Utah, Salt Lake City, UT USA; 10 Division of Hematology and Medical Oncology, Keck School of Medicine of USC, Norris Comprehensive Cancer Center, Los Angeles, CA USA; 11 Division of Hematology and Medical Oncology, University of Pennsylvania Abramson Cancer Center, Philadelphia, PA USA; 12 Division of Hematology and Medical Oncology, University of Wisconsin Carbone Cancer Center, Madison, WI USA; 13 Genentech, South San Francisco, CA USA; 14 Department of Pathology, Tisch Cancer Institute, Icahn School of Medicine at Mount Sinai, New York, NY USA; 15 Human Immune Monitoring Center, Icahn School of Medicine at Mount Sinai, New York, NY USA; 16 Precision Immunology Institute, Icahn School of Medicine at Mount Sinai, New York, NY USA; 17 Department of Genetics and Genomic Sciences, Icahn School of Medicine at Mount Sinai, New York, NY USA; 18 Icahn Institute for Data Science and Genomic Technology, Icahn School of Medicine at Mount Sinai, New York, NY USA; 19 Department of Pathology, Molecular and Cell-based Medicine, Icahn School of Medicine at Mount Sinai, New York, NY USA; 20 Gene Dx, Stamford, CT USA; 21 Department of Radiation Oncology, Dana-Farber Cancer Institute/Brigham & Women’s Hospital, Harvard Medical School, Boston, MA USA; 22 Department of Radiation Oncology, Tisch Cancer Institute, Icahn School of Medicine at Mount Sinai, New York, NY USA; 23 Department of Urology, Tisch Cancer Institute, Icahn School of Medicine at Mount Sinai, New York, NY USA; 24 Department of Biostatistics and Medical Informatics, University of Wisconsin Carbone Cancer Center, Madison, WI USA; 25 Present Address: Formerly with the Icahn School of Medicine at Mount Sinai, New York, NY USA

**Keywords:** Phase II trials, Translational research, Bladder cancer, Cancer immunotherapy, Chemotherapy

Correction to: *Nature Medicine* 10.1038/s41591-023-02568-1, published online 2 October 2023.

In the version of the article initially published, Diane M. Del Valle (Precision Immunology Institute, Icahn School of Medicine at Mount Sinai, New York, NY, USA) was missing from the author list; the surname of Amir Horowitz appeared incorrectly (Horwitz); the ClinicalTrials.gov identifier in the Abstract (now listing NCT03558087) was incorrect; and the Acknowledgements were missing additional thanks. The updates now appear in the HTML and PDF versions of the article.

