# Supplementary material for: Gemcitabine and cisplatin plus nivolumab as organ-sparing treatment for muscle-invasive bladder cancer: a phase 2 trial
Source: Nat Med. 2023 Oct 2;29(11):2825–34. doi: 10.1038/s41591-023-02568-1 (PMC10667093; doi:10.1038/s41591-023-02568-1)
Supplement: Supplementary file 1 — Supplementary Tables 1–5. [file 41591_2023_2568_MOESM1_ESM.pdf]

nature medicine

Article https://doi.org/10.1038/s41591-023-02568-1

Gemcitabine and cisplatin plus nivolumab as organ-sparing treatment for muscle-invasive bladder cancer: a phase 2 trial

In the format provided by the authors and unedited

nature medicine

Article https://doi.org/10.1038/s41591-023-02568-1

Gemcitabine and cisplatin plus nivolumab as organ-sparing treatment for muscle-invasive bladder cancer: a phase 2 trial

In the format provided by the authors and unedited

Inventory of Supporting Information

Supplementary Table 1. Clinical stage at the time of recurrence and final pathological stage in patients with local recurrence after achieving a clinical complete response and forgoing initial cystectomy

Supplementary Table 2. Restaging clinical T stage after 4 cycles of gemcitabine, cisplatin, plus nivolumab and final cystectomy pathological stage among patients not achieving a clinical complete response

Supplementary Table 3. Treatment emergent adverse events occurring in ≥1 patient(s)* (n=76 patients) per National Cancer Institute Common Terminology Criteria for Adverse Events (NCI CTCAE v4.03)

Supplementary Table 4. Contingency tables for positive predictive value of clinical complete response +/-prespecified genomic alterations for composite outcome measure of 2-year *bladder-intact overall survival* in patients forgoing immediate cystectomy or ≤ ypT1N0 in patients undergoing immediate cystectomy

Supplementary Table 5. Mass Cytometry Metals and Antibodies

Supplementary Table 1. Clinical stage at the time of recurrence and final pathological stage in patients with local recurrence after achieving a clinical complete response and forgoing initial cystectomy

cT stage at time of local recurrence	Cystectomy T stage	Cystectomy N Stage	Consensus Pathological Stage*
cT2	pT1	pN0	pT2N0
cT2	pT2	pN0	pT2N0
NA**	pT0	pN0	pT0N0
cTA	pT2	pN0	pT2N0
cTA	pT2	pN0	pT2N0
cT2	pT4	pN1	pT4N1
cT2	pTA	pN0	pT2N0
cT1	pTis	pN0	pT1N0

cT stage, clinical T stage; T, tumor; N, Node
*Consensus staging integrates highest T stage from restaging TURBT and cystectomy T stage
**Recurrence was determined based on MRI and no restaging cystoscopy or TURBT was performed

Supplementary Table 2. Restaging clinical T stage after 4 cycles of gemcitabine, cisplatin, plus nivolumab and final cystectomy pathological stage among patients not achieving a clinical complete response

Restaging T-stage	Cystectomy T stage	Cystectomy N Stage	Consensus Pathological Stage*
ycTis	ypT1	ypN0	ypT1N0
ycTis	ypT2A	ypN0	ypT2N0
ycTis	ypT3B	ypN0	ypT3N0
ycTis	ypTis	ypN0	ypTisN0
ycTis		Declined local treatment	
ycTis		Pursued radiation therapy	
ycT1	ypT1	ypN0	ypT1N0
ycT1	ypT1	ypN0	ypT1N0
ycT1	ypT4	ypN2	ypT4N0
ycT1	ypT2	ypN0	ypT2N0
ycT1	ypT2	ypN0	ypT2N0
ycT1	ypT2	ypN0	ypT2N0
ycT1	ypTis	ypN0	ypT1N0
ycT1	ypTis	ypN1	ypT1N1
ycT1		Pursued radiation therapy	
ycT1		Pursued radiation therapy	
ycT2	ypT0	ypN0	ypT2N0
ycT2	ypT2	ypN0	ypT2N0
ycT2	ypT2	ypN1	ypT2N1
ycT2	ypT3	ypN0	ypT3N0
ycT2	ypT3	ypN1	ypT3N1
ycT2	ypT4	ypN0	ypT4N0
ycT2	ypTis	ypN0	ypT2N0
ycT2	ypTis	ypN0	ypT2N0
ycT2	ypTis	ypN0	ypT2N0
ycT2	ypTis	ypN0	ypT2N0
ycT2		Pursued radiation therapy	
ycT3	ypT3	ypN2	ypT3N2
ycT3	ypT2	ypN1	ypT3N1
ycT3	ypT2	ypN1	ypT3N1
ycT3	ypT3	ypN2	ypT3N2
ycT3	ypT3	ypN0	ypT3N0
ycT3	ypT3	ypN2	ypT3N2
ycT3	ypTis	ypN0	ypT3N0
ycT4	ypT4	ypN2	ypT4N2
ycT4	ypT4	ypN2	ypT4N2
X**	ypT0	ypN0	ypT0N0
X***	ypT0	ypN0	ypT0N0
X***	ypT3	ypN0	ypT3N0
X***	ypT4	ypN1	ypT4N1
X**	ypT3	ypN0	ypT3N0
X**	ypT3	ypN1	ypT3N1
X**		Developed metastatic disease	

*Consensus staging integrates highest T stage from restaging TURBT and cystectomy T stage
**Did not undergo clinical restaging
***Underwent restaging MRI but no cystoscopy or biopsies

Supplementary Table 3. Number of treatment emergent adverse events occurring in ≥1 patient(s)* (n=76 patients) per National Cancer Institute Common Terminology Criteria for Adverse Events (NCI CTCAE v4.03)

Adverse Events	Grade 1	Grade 2	Grade 3	Grade 4	Grade 5	Total
Any adverse event	4	15	41	15	1**	76
Fatigue	46	12	0	0	0	58
Anemia	22	23	12	0	0	57
Neutrophil count decreased	7	19	21	5	0	52
Nausea	39	5	0	0	0	44
Constipation	38	1	0	0	0	39
Creatinine increased	23	15	0	0	0	38
Hypertension	19	13	4	0	0	36
Hyperglycemia	27	5	2	0	0	34
White blood cell decreased	17	11	3	0	0	31
Platelet count decreased	21	5	2	2	0	30
Hematuria	25	1	3	0	0	29
Anorexia	18	5	0	0	0	23
Hyponatremia	17	1	4	1	0	23
Tinnitus	22	1	0	0	0	23
Dyspnea	19	2	1	0	0	22
Alopecia	21	0	0	0	0	21
Urinary tract infection	3	5	13	0	0	21
Back pain	15	2	0	0	0	17
Cholesterol high	15	2	0	0	0	17
Insomnia	13	2	0	0	0	15
Urinary frequency	14	1	0	0	0	15
Hypomagnesemia	7	7	0	0	0	14
Vomiting	12	1	1	0	0	14
Abdominal pain	7	4	2	0	0	13
Rash maculo-papular	11	2	0	0	0	13
Diarrhea	11	1	0	0	0	12
Hypoalbuminemia	9	3	0	0	0	12
Hypokalemia	9	0	3	0	0	12
Pain	10	1	1	0	0	12
Proteinuria	5	7	0	0	0	12
Urinary tract pain	10	2	0	0	0	12
Headache	10	1	0	0	0	11
Hypocalcemia	9	2	0	0	0	11
Pruritus	10	1	0	0	0	11
Skin and subcutaneous tissue disorders	9	2	0	0	0	11
Weight loss	5	6	0	0	0	11
Alanine aminotransferase increased	8	2	0	0	0	10
Anxiety	6	2	1	0	0	9
Aspartate aminotransferase increased	7	1	1	0	0	9
Dizziness	8	1	0	0	0	9
Dysgeusia	8	1	0	0	0	9
Peripheral sensory neuropathy	9	0	0	0	0	9
Urinary urgency	9	0	0	0	0	9
Cough	8	0	0	0	0	8
Depression	5	1	1	1	0	8
Edema limbs	8	0	0	0	0	8
Hyperkalemia	7	1	0	0	0	8
Musculoskeletal disorder	6	1	0	0	0	7
Serum amylase increased	4	2	0	1	0	7
Acute kidney injury	1	1	4	0	0	6
Alkaline phosphatase increased	5	0	1	0	0	6
Arthritis	5	1	0	0	0	6

Gastroesophageal reflux disease	4	2	0	0	0	6
Gastrointestinal disorders - other	5	0	1	0	0	6
Genera /administration site conditions	4	2	0	0	0	6
Infections and infestations (other)	2	2	2	0	0	6
Thromboembolic event	2	0	4	0	0	6
Dry skin	5	0	0	0	0	5
Fever	2	1	2	0	0	5
Hearing impaired	5	0	0	0	0	5
Hypophosphatemia	3	1	1	0	0	5
Lipase increased	2	2	1	0	0	5
Respiratory or thoracic disorders	2	3	0	0	0	5
Sepsis	0	0	0	4	1	5
Arthralgia	4	0	0	0	0	4
Blurred vision	4	0	0	0	0	4
Epistaxis	3	1	0	0	0	4
Generalized muscle weakness	2	1	1	0	0	4
Hyperthyroidism	3	0	1	0	0	4
Hyperuricemia	4	0	0	0	0	4
Hypoglycemia	4	0	0	0	0	4
Hypotension	0	1	3	0	0	4
Lymphocyte count decreased	3	0	1	0	0	4
Pain in extremity	4	0	0	0	0	4
Tremor	3	1	0	0	0	4
Urinary incontinence	2	2	0	0	0	4
Atrial fibrillation	1	2	0	0	0	3
Bladder spasm	2	1	0	0	0	3
Bloating	2	1	0	0	0	3
Dehydration	1	0	2	0	0	3
Fall	2	1	0	0	0	3
Hiccups	3	0	0	0	0	3
Injection site reaction	3	0	0	0	0	3
Nasal congestion	2	1	0	0	0	3
Pelvic pain	2	1	0	0	0	3
Rash acneiform	3	0	0	0	0	3
Reproductive system and breast disorders	3	0	0	0	0	3
Sleep apnea	2	0	1	0	0	3
Superficial thrombophlebitis	0	3	0	0	0	3
Toothache	3	0	0	0	0	3
Partial thromboplastin time prolonged	2	0	0	0	0	2
Blood bilirubin increased	0	1	1	0	0	2
Chills	2	0	0	0	0	2
Dry mouth	2	0	0	0	0	2
Erectile dysfunction	0	1	1	0	0	2
Flank pain	2	0	0	0	0	2
Flushing	2	0	0	0	0	2
Gait disturbance	1	1	0	0	0	2
Hoarseness	2	0	0	0	0	2
Hypermagnesemia	2	0	0	0	0	2
Hypothyroidism	1	1	0	0	0	2
Ileus	1	1	0	0	0	2
Infusion related reaction	1	1	0	0	0	2
Kidney infection	0	0	2	0	0	2
Mucositis oral	2	0	0	0	0	2
Phlebitis	2	0	0	0	0	2
Presyncope	0	2	0	0	0	2
Productive cough	2	0	0	0	0	2
Rash pustular	2	0	0	0	0	2
Sinus tachycardia	1	1	0	0	0	2
Sore throat	2	0	0	0	0	2

Stoma site infection	2	0	0	0	0	2
Syncope	1	0	1	0	0	2
Upper respiratory infection	0	2	0	0	0	2
Urinary retention	2	0	0	0	0	2
Weight gain	1	0	1	0	0	2

*maximum grade event for each term per patient
**not attributed to systemic therapy

Supplementary Table 4. Contingency table for positive predictive value of clinical complete response +/- prespecified genomic alterations for composite outcome measure of 2-year *bladder-intact* overall survival in patients forgoing immediate cystectomy or ≤ ypT1N0 in patients undergoing immediate cystectomy.

		Composite outcome measure	
		Yes	No
Clinical complete response*	Yes	23	9
	No	4	33
Clinical complete response + any mutation in *FANCC*, A*TM*, and/or *RB1*†	Yes	8	3
	No	18	37
Clinical complete response + any pathogenic mutation in *FANCC*, A*TM*, and/or *RB1*†	Yes	5	0
	No	21	40
Clinical complete response + any mutation in ERCC2†	Yes	3	2
	No	23	38
Clinical complete response + any pathogenic mutation in *ERCC2*†	Yes	2	2
	No	24	38
Clinical complete response + any mutation in *FANCC*, A*TM*, *RB1, and/or ERCC2*†	Yes	10	4
	No	16	36
Clinical complete response + any pathogenic mutation in *FANCC*, A*TM*, *RB1, and/or ERCC2*†	Yes	6	2
	No	20	38
Clinical complete response + TMB ≥ 10 mut/Mb‡	Yes	15	6
	No	9	34
Clinical complete response + any mutation in *FANCC*, A*TM*, *RB1, ERCC2,* and/or TMB ≥ 10 mut/Mb†	Yes	16	6
	No	10	34

TMB, tumor mutational burden; BH FDRs, Benjamini-Hochberg False Discovery Rate

*Analysis of clinical complete response alone includes n=69 (of 76 total study patients). Seven patients excluded for following reasons: 4 patients who did not undergo clinical response assessment, 2 patients not achieving a clinical complete response, not pursuing cystectomy, and lost to follow-up prior to 2 years, and 1 patient achieving a clinical complete response and without evidence of local or distant recurrence at 18 months and subsequently lost to follow-up).

†Analyses of genomic alterations in *ATM*, *FANCC*, and *RB1* include n=66 (of 76 total study patients) for each matrix. Ten patients excluded from the analysis for the following reasons: 4 patients who did not undergo clinical response assessment, 2 patients not achieving a clinical complete response, not pursuing cystectomy, and lost to follow-up prior to 2 years, 1 patient achieving a clinical complete response and without evidence of local or distant recurrence at 18 months and subsequently lost to follow-up, and 3 patients without DNA sequencing data.

‡Analyses of TMB includes n=64 (of 76 total study patients) for each matrix. Twelve patients excluded from the analysis for the following reasons: 4 patients who did not undergo clinical response assessment, 2 patients not achieving a clinical complete response, not pursuing cystectomy, and lost to follow-up prior to 2 years, 1 patient achieving a clinical complete response and without evidence of local or distant recurrence at 18 months and subsequently lost to follow-up, and 5 patients without DNA sequencing data and/or for whom TMB could not be calculated.

Supplementary Table 5. Mass Cytometry Metals and Antibodies

Channel	Target	Clone	Manufacturer	Catalog #
89Y	CD45	HI30	Standard Bio Tools	3089003B
113In	CD57	HNK-1	Biolegend	359602
115In	CD11c	BU15	Biolegend	337202
141Pr	CD33	WM53	Biolegend	303410
142Nd	CD19	REA675	Miltenyi	130-122-301
143Nd	CD45RA	REA562	Miltenyi	130-122-292
144Nd	CD141	Phx-01	Biolegend	902101
145Nd	CD4	REA623	Miltenyi	130-122-283
146Nd	CD8	REA734	Miltenyi	130-122-281
147Sm	CLEC9A	8F9	Miltenyi	130-122-306
148Nd	CD16	REA423	Miltenyi	130-108-027
149Sm	CD127	A019D5	Standard Bio Tools	3149011B
150Nd	CD1c	REA694	Miltenyi	130-122-298
151Eu	CD123	REA918	Miltenyi	130-122-297
152Sm	CD66b	REA306	Miltenyi	130-108-019
154Sm	ICOS	C398.4A	Biolegend	313502
155Gd	CD27	REA499	Miltenyi	130-122-295
156Gd	PD-L1	29E.2A3	Biolegend	329710
158Gd	CD103	Ber-ACT8	BioLegend	350202
159Tb	CD24	ML5	Biolegend	311102
160Gd	CD14	REA599	Miltenyi	130-122-290
161Dy	CD56	REA196	Miltenyi	130-108-016
162Dy	gdTCR	REA591	Miltenyi	130-122-291
163Dy	CXCR5	REA103	Miltenyi	130-122-325
164Dy	CD69	FN50	Biolegend	310939
165Ho	CD64	10.1	Biolegend	305016
166Er	41BB	4B4-1	Biolegend	309802
167Er	CCR7	REA546	Miltenyi	130-122-300
168Er	CD3	REA613	Miltenyi	130-122-282
169Tm	CD25	REA570	Miltenyi	130-122-302
170Er	CD38	REA671	Miltenyi	130-122-288
171Yb	CD161	HP-3G10	BioLegend	339902
172Yb	CD39	A1	Biolegend	328202
173Yb	CXCR3	REA232	Miltenyi	130-108-022
174Yb	HLADR	REA805	Miltenyi	130-122-299
175Lu	PD-1	EH12.2H7	Standard Bio Tools	3174020B
176Yb	CCR4	REA279	Miltenyi	130-122-323
209Bi	CD11b	ICRF44	Standard Bio Tools	3209003B
