# Supplementary material for: Gemcitabine and cisplatin plus nivolumab as organ-sparing treatment for muscle-invasive bladder cancer: a phase 2 trial
Source: Nat Med. 2023 Oct 2;29(11):2825–34. doi: 10.1038/s41591-023-02568-1 (PMC10667093; doi:10.1038/s41591-023-02568-1)
Supplement: Supplementary file 2 — Reporting Summary [file 41591_2023_2568_MOESM2_ESM.pdf]

nature portfolio

Corresponding author(s): Matthew Galsky, MD

Last updated by author(s): Aug 19, 2023

Reporting Summary

Nature Portfolio wishes to improve the reproducibility of the work that we publish. This form provides structure for consistency and transparency in reporting. For further information on Nature Portfolio policies, see our Editorial Policies and the Editorial Policy Checklist.

Statistics

For all statistical analyses, confirm that the following items are present in the figure legend, table legend, main text, or Methods section.

n/a	Confirmed	
☐	☒	The exact sample size (*n*) for each experimental group/condition, given as a discrete number and unit of measurement
☒	☐	A statement on whether measurements were taken from distinct samples or whether the same sample was measured repeatedly
☐	☒	The statistical test(s) used AND whether they are one- or two-sided<br>*Only common tests should be described solely by name; describe more complex techniques in the Methods section.*
☐	☒	A description of all covariates tested
☐	☒	A description of any assumptions or corrections, such as tests of normality and adjustment for multiple comparisons
☐	☒	A full description of the statistical parameters including central tendency (e.g. means) or other basic estimates (e.g. regression coefficient) AND variation (e.g. standard deviation) or associated estimates of uncertainty (e.g. confidence intervals)
☐	☒	For null hypothesis testing, the test statistic (e.g. *F*, *t*, *r*) with confidence intervals, effect sizes, degrees of freedom and *P* value noted<br>*Give P values as exact values whenever suitable.*
☒	☐	For Bayesian analysis, information on the choice of priors and Markov chain Monte Carlo settings
☒	☐	For hierarchical and complex designs, identification of the appropriate level for tests and full reporting of outcomes
☒	☐	Estimates of effect sizes (e.g. Cohen's *d*, Pearson's *r*), indicating how they were calculated

Our web collection on statistics for biologists contains articles on many of the points above.

Software and code

Policy information about availability of computer code

Data collection	The OnCore Clinical Trial Management System was used for data collection
Data analysis	Software used for data analysis included: R, Astrolabe, Fluidigm Mass Cytometry software

For manuscripts utilizing custom algorithms or software that are central to the research but not yet described in published literature, software must be made available to editors and reviewers. We strongly encourage code deposition in a community repository (e.g. GitHub). See the Nature Portfolio guidelines for submitting code & software for further information.

Data

Policy information about availability of data

All manuscripts must include a data availability statement. This statement should provide the following information, where applicable:

- Accession codes, unique identifiers, or web links for publicly available datasets
- A description of any restrictions on data availability
- For clinical datasets or third party data, please ensure that the statement adheres to our policy

In accordance with NIH’s Genomic Data Sharing Policy, the DNA sequencing data used to support the findings of this study has been deposited under controlled-access in the database of Genotypes and Phenotypes (dbGaP) under the accession number phs0003372. Genomic, clinical, mass cytometry, and protein analyte data from this study used to support this publication will be made available upon reasonable request from a qualified medical or scientific professional for the

specific purpose laid out in that request and may include de-identified individual participant data. Requests for secondary use of this data will require completing a data use agreement (https://osp.od.nih.gov/wp-content/uploads/Model_DUC.pdf) and submitting a data access request to NIH.

Human research participants

Policy information about studies involving human research participants and Sex and Gender in Research.

Reporting on sex and gender	Our study reports a phase 2 trial in patients with muscle-invasive bladder cancer. Men are approximately 4 times more likely to be diagnosed with bladder cancer. Our study enrolled 79% men and 21% women.
Population characteristics	The baseline characteristics are outlined in Table 1 of the manuscript and are consistent with the demographics of muscle-invasive bladder cancer in the United States.
Recruitment	Patients were recruited from urology and medical oncology clinics at participating institutions. Patients were recruited from those seeking standard evaluation and clinical care for muscle-invasive bladder cancer in these clinics and all patients seen for routine clinical care were offered the option of trial participation if deemed potentially eligible.
Ethics oversight	The protocol was approved by local ethics committees at the Icahn School of Medicine at Mount Sinai, City of Hope Comprehensive Cancer Centers, Huntsman Cancer Institute University of Utah, Oregon Health and Science University, Penn Medicine Abramson Cancer Center, Rutgers Cancer Institute of New Jersey, University of Southern California, and University of Wisconsin and written informed consent was provided by all patients prior to enrollment.

Note that full information on the approval of the study protocol must also be provided in the manuscript.

Field-specific reporting

Please select the one below that is the best fit for your research. If you are not sure, read the appropriate sections before making your selection.

☒ Life sciences ☐ Behavioural & social sciences ☐ Ecological, evolutionary & environmental sciences

For a reference copy of the document with all sections, see nature.com/documents/nr-reporting-summary-flat.pdf

Life sciences study design

All studies must disclose on these points even when the disclosure is negative.

Sample size	The co-primary objectives of the study were to: (a) determine the cCR rate with gemcitabine, cisplatin, plus nivolumab and (b) determine the ability of cCR to predict clinical benefit from treatment. Clinical benefit was defined as either: (a) being metastasis-free at 2 years in patients achieving a cCR and opting to not undergo immediate cystectomy or (b) achieving a pCR (<pT1) in patients with a cCR opting for immediate cystectomy. Secondary objectives included the association between genomic alterations in a prespecified panel of genes detected in pre-treatment TURBT tissue (ERCC2, ATM, RB1, and FANCC 15–22), as well as tumor mutational burden (using an established cut-point of ⍰ 10 mutations/Mb23,24), and clinical outcomes. Additional secondary objectives included safety, metastasis-free survival, overall survival, and bladder-intact survival.<br>The sample size was based on the following assumptions: (a) patients without a cCR would not be suitable to forgo cystectomy, (b) ~40% of enrolled patients would achieve a cCR, and (c) ~35% of enrolled patients would achieve clinical benefit. Therefore, our assumption implied that the negative predictive value of a cCR would be 1. The sample size was based on the confidence interval width of the positive predictive value of cCR for clinical benefit and generated such that the lower bound of the 95% one-sided confidence interval exceeded 80%. This required enrollment of 68 patients and the sample size was increased to 76 to account for potential missing data.
Data exclusions	No data were excluded from the analysis.
Replication	This was a prospective clinical trial and replication was not within the scope of the trial.
Randomization	This was a phase 2 single arm trial employing a risk-adapted strategy and randomization was not deemed appropriate to the design prior to conducting this phase 2 portion which could then be used to inform the design of a randomized trial.
Blinding	There was no randomization in this trial and therefore blinding was not applicable.

Reporting for specific materials, systems and methods

We require information from authors about some types of materials, experimental systems and methods used in many studies. Here, indicate whether each material, system or method listed is relevant to your study. If you are not sure if a list item applies to your research, read the appropriate section before selecting a response.

Materials & experimental systems

n/a	Involved in the study	
☐	☒	Antibodies
☒	☐	Eukaryotic cell lines
☒	☐	Palaeontology and archaeology
☒	☐	Animals and other organisms
☐	☒	Clinical data
☒	☐	Dual use research of concern

Methods

n/a	Involved in the study	
☒	☐	ChIP-seq
☒	☐	Flow cytometry
☒	☐	MRI-based neuroimaging

Antibodies

Antibodies used

Channel Target Clone Manufacturer Catalog #
89Y CD45 HI30 Standard Bio Tools 3089003B
113In CD57 HNK-1 Biolegend 359602
115In CD11c BU15 Biolegend 337202
141Pr CD33 WM53 Biolegend 303410
142Nd CD19 REA675 Miltenyi 130-122-301
143Nd CD45RA REA562 Miltenyi 130-122-292
144Nd CD141 Phx-01 Biolegend 902101
145Nd CD4 REA623 Miltenyi 130-122-283
146Nd CD8 REA734 Miltenyi 130-122-281
147Sm CLEC9A 8F9 Miltenyi 130-122-306
148Nd CD16 REA423 Miltenyi 130-108-027
149Sm CD127 A019D5 Standard Bio Tools 3149011B
150Nd CD1c REA694 Miltenyi 130-122-298
151Eu CD123 REA918 Miltenyi 130-122-297
152Sm CD66b REA306 Miltenyi 130-108-019
154Sm ICOS C398.4A Biolegend 313502
155Gd CD27 REA499 Miltenyi 130-122-295
156Gd PD-L1 29E.2A3 Biolegend 329710
158Gd CD103 Ber-ACT8 BioLegend 350202
159Tb CD24 ML5 Biolegend 311102
160Gd CD14 REA599 Miltenyi 130-122-290
161Dy CD56 REA196 Miltenyi 130-108-016
162Dy gdTCR REA591 Miltenyi 130-122-291
163Dy CXCR5 REA103 Miltenyi 130-122-325
164Dy CD69 FN50 Biolegend 310939
165Ho CD64 10.1 Biolegend 305016
166Er 41BB 4B4-1 Biolegend 309802
167Er CCR7 REA546 Miltenyi 130-122-300
168Er CD3 REA613 Miltenyi 130-122-282
169Tm CD25 REA570 Miltenyi 130-122-302
170Er CD38 REA671 Miltenyi 130-122-288
171Yb CD161 HP-3G10 BioLegend 339902
172Yb CD39 A1 Biolegend 328202
173Yb CXCR3 REA232 Miltenyi 130-108-022
174Yb HLADR REA805 Miltenyi 130-122-299
175Lu PD-1 EH12.2H7 Standard Bio Tools 3174020B
176Yb CCR4 REA279 Miltenyi 130-122-323
209Bi CD11b ICRF44 Standard Bio Tools 3209003B

Validation

All antibodies were either purchased pre-conjugated from Fluidigm (Fluidigm, South San Francisco, CA) or conjugated in-house (using commercial X8 polymer conjugation kits purchased from Fluidigm) at the Human Immune Monitoring Center (HIMC), Icahn School of Medicine at Mount Sinai, New York. All in-house conjugated antibodies were titrated and validated on healthy donor PBMCs.

Clinical data

Policy information about clinical studies
All manuscripts should comply with the ICMJE guidelines for publication of clinical research and a completed CONSORT checklist must be included with all submissions.

Clinical trial registration

NCT03558087

Study protocol

Attached as supplemental data

Data collection

Between 8/2018-11/2020, 76 patients were enrolled at seven medical centers in the United States. The data were collected by medical oncology clinical research teams at the Icahn School of Medicine at Mount Sinai, City of Hope Comprehensive Cancer Centers, Huntsman Cancer Institute University of Utah, Oregon Health and Science University, Penn Medicine Abramson Cancer Center, Rutgers Cancer Institute of New Jersey, University of Southern California, and University of Wisconsin.

Outcomes

Our primary goal was to test whether uniformly assessed and consistently defined cCR could identify patients who could safely forgo immediate cystectomy. We reasoned that a potentially effective personalized risk-adapted strategy would: (a) tolerate missing some patients who might have been suitable candidates to forgo immediate cystectomy in favor of maximizing identification of patients who fare well without immediate cystectomy and (b) incorporate the ability of later cystectomy to achieve favorable cancer-related outcomes in the subset of patients with a cCR experiencing local recurrence after initial surveillance. Therefore, our primary objectives were to estimate the cCR rate and to assess the positive predictive value of cCR for a composite outcome measure (2-year metastasis-free survival in patients forgoing immediate cystectomy or <ypT1N0 in patients electing immediate cystectomy. Secondary outcomes included safety, the association between a prespecified panel of genomic biomarkers and clinical complete response and metastasis free survival, metastasis free survival, and overall survival. These outcomes were predefined at the time of designing the study as were used to inform the clinical trial protocol.
