# Supplementary material for: Gemcitabine and cisplatin plus nivolumab as organ-sparing treatment for muscle-invasive bladder cancer: a phase 2 trial
Source: Nat Med. 2023 Oct 2;29(11):2825–34. doi: 10.1038/s41591-023-02568-1 (PMC10667093; doi:10.1038/s41591-023-02568-1)
Supplement: Supplementary file 3 — Clinical Trial Protocol. [file 41591_2023_2568_MOESM3_ESM.pdf]

Neoadjuvant gemcitabine, cisplatin, plus nivolumab in patients with muscle-invasive bladder cancer with selective bladder sparing

Sponsor Investigator

Matthew D. Galsky, MD
Icahn School of Medicine at Mount Sinai
Tisch Cancer Institute

Co-Principal Investigators

Sumanta Pal, MD
City of Hope Comprehensive Cancer Center

Sia Daneshmand, MD
University of Southern California

Statistician

Menggang Yu, PhD

Trial Management Provided by
Hoosier Cancer Research Network, Inc.
7676 Interactive Way Suite 120
Indianapolis, IN 46278

Trial Supported by
Bristol-Myers Squibb Company
CA209-9AU

Investigational New Drug (IND) #: 136858

Initial Protocol Version Date: 01MAY2018
Protocol Amendment Version Date:
11JUL2019
15JUL2020
29DEC2020
12AUG2021

PROTOCOL SIGNATURE PAGE

Neoadjuvant gemcitabine, cisplatin, plus nivolumab in patients with muscle-invasive bladder cancer with selective bladder sparing

VERSION DATE: 12AUG2021

I confirm I have read this protocol, I understand it, and I will work according to this protocol and to the ethical principles stated in the latest version of the Declaration of Helsinki, the applicable guidelines for good clinical practices, whichever provides the greater protection of the individual. I will accept the monitor's overseeing of the study. I will promptly submit the protocol to applicable institutional review board(s).

________________________________ __________________________

Signature of Site Investigator Date

____

Site Investigator Name (printed)

____

Site Investigator Title

____

Name of Facility

____

Location of Facility (City and State)

PLEASE COMPLETE AND EMAIL A COPY TO HCRN

SYNOPSIS

**TITLE**	Neoadjuvant gemcitabine, cisplatin, plus nivolumab in patients with muscle-invasive bladder cancer with selective bladder sparing
**PHASE**	II
**OBJECTIVES**	**Primary Objectives**<br>• Determine the clinical complete response rate with gemcitabine, cisplatin, plus nivolumab<br>• Determine the ability of clinical complete response (cT0 or cTa) to predict benefit from treatment. Benefit will be defined as a pathologic complete response (<pT1) in patients undergoing cystectomy and 2 year metastasis-free in patients pursuing surveillance<br><br>**Secondary Objectives**<br>• Determine the safety of neoadjuvant gemcitabine, cisplatin, plus nivolumab<br>• Describe the proportion of patients with a clinical complete response to pursue cystectomy versus surveillance<br>• Determine the association between a prespecified panel of genomic biomarkers and benefit from treatment in patients achieving a clinical complete response. Benefit will be defined as a pathologic complete response (p<T1) in patients undergoing cystectomy and 2 years metastasis-free in patients pursuing surveillance<br>• Determine the pathologic complete response rate (<pT1) in patients pursuing cystectomy<br>• Determine the 2-year metastasis free survival.<br>• Determine overall survival (and bladder-intact overall survival rate)<br>• Determine the recurrence-free survival<br><br>**Exploratory Objectives**<br>• Explore the association of immune biomarkers, genomic alterations, circulating tumor DNA, gene expression, and radiographic characteristics (including radiomics) with clinical complete response rate, <pT1 rate (where applicable), recurrence-free survival, the bladder-intact overall survival, and overall survival<br>• Explore potential genomic and immunologic biomarkers in the urine and blood that correlate with treatment benefit

STUDY DESIGN

This is a phase 2 trial seeking to define the safety and activity of gemcitabine, cisplatin, plus nivolumab as neoadjuvant therapy in patients with muscle-invasive bladder cancer and to define the role of clinical complete response in predicting benefit in patients opting to avoid cystectomy

KEY ELIGIBILITY CRITERIA (See Section 3 for FULL eligibility criteria)

Inclusion Criteria

1. Written informed consent and HIPAA authorization for release of personal health information prior to registration. **NOTE:** HIPAA authorization may be included in the informed consent or obtained separately.
2. Age ≥ 18 years at the time of consent.
3. ECOG Performance Status of ≤ 1 within 28 days prior to registration.
4. Histological evidence of clinically localized muscle-invasive urothelial cancer of the bladder (i.e., cT2-4N0M0). Candidate for cystectomy as per treating physician.
5. Demonstrate adequate organ function as defined in the table below. All screening labs to be obtained within 28 days prior to registration.

**System**	**Laboratory Value**
**Hematological**	
Absolute Neutrophil Count (ANC)	≥ 1.5 x 10^9/L
Hemoglobin (Hgb)	≥ 9 g/dL
Platelets	≥ 100 x 10^9/L
**Renal**	
Calculated creatinine clearance[1]	Creatinine ≤ 1.5 or creatinine clearance ≥ 60 mL/min
**Hepatic**	
Bilirubin	≤ 1.5 × upper limit of normal (ULN) (except subjects with Gilbert Syndrome, who can have total bilirubin < 3.0 mg/dL)
Aspartate aminotransferase (AST)	≤ 3 × ULN
Alanine aminotransferase (ALT)	≤ 3 × ULN

1 Cockcroft-Gault formula will be used to calculate creatinine clearance

6. All subjects must have adequate archival tissue identified at screening (i.e., at least 15 unstained slides or paraffin block). Subjects without archival tissue must be discussed with the sponsor-investigator.

7. Women of childbearing potential must have a negative serum or urine pregnancy within 7 days prior to C1D1. **NOTE: "**Women of childbearing potential" is defined as any female who has experienced menarche and who has not undergone surgical sterilization (hysterectomy or bilateral oophorectomy) or who is not postmenopausal. Menopause is defined clinically as 12 months of amenorrhea in a woman over 45 in the absence of other biological or

physiological causes. In addition, women under the age of 62 must have a documented serum follicle stimulating hormone (FSH) level less than 40 mIU/mL.

NOTE: Women of childbearing potential (WOCBP) receiving nivolumab must be willing to abstain from heterosexual intercourse or to use 2 forms of effective methods of contraception from the time of informed consent to 5 months after the last dose of nivolumab or for the timeframe outlined per package insert for chemotherapy. This timeframe also applies to breastfeeding. The two contraception methods can be comprised of two barrier methods, or a barrier method plus a hormonal method.

Male subjects capable of fathering a child that are sexually active with partners of childbearing potential must be willing to abstain from heterosexual intercourse or to use 2 forms of effective methods of contraception from the time of informed consent to the timeframe outlined per package insert for chemotherapy. Contraception is not required for nivolumab. The timeframes described in the previous 2 sentences apply to sperm donation. Two contraception methods can be comprised of two barrier methods, or a barrier method plus a hormonal method.

Exclusion Criteria

1. Prior treatment with systemic chemotherapy for muscle-invasive urothelial cancer of the bladder
2. Active infection requiring systemic therapy
3. Pregnant or breastfeeding (**NOTE:** breast milk cannot be stored for future use while the mother is being treated on study).
4. Any serious or uncontrolled medical disorder that, in the opinion of the investigator, may increase the risk associated with study participation or study drug administration, impair the ability of the subject to receive protocol therapy, or interfere with the interpretation of study results.
5. Prior malignancy active within the previous 3 years except for locally curable cancers that have been apparently cured.
6. Subjects with active, known or suspected autoimmune disease. Subjects with vitiligo, type I diabetes mellitus, residual hypothyroidism due to autoimmune condition only requiring hormone replacement, psoriasis not requiring systemic treatment, or conditions not expected to recur in the absence of an external trigger are permitted to enroll.
7. Subjects with a condition requiring systemic treatment with either corticosteroids (> 10 mg daily prednisone equivalents) or other immunosuppressive medications within 14 days of study drug administration. Inhaled or topical steroids and adrenal replacement doses > 10 mg daily prednisone equivalents are permitted in the absence of active autoimmune disease.

	8. Prior treatment with an anti-PD-1, anti-PD-L1, anti-PD-L2, anti-CTLA-4 antibody, or any other antibody or drug specifically targeting T-cell co-stimulation or immune checkpoint pathways.<br>9. Grade ≥ 2 neuropathy (NCI CTCAE version 4.03).<br>10. Prior radiation therapy for bladder cancer<br>11. Positive test for hepatitis B virus surface antigen (HBV sAg) or hepatitis C virus ribonucleic acid (RNA) or hepatitis C antibody (HCV antibody) indicating acute or chronic infection.<br>12. Known history of testing positive for human immunodeficiency virus (HIV) or known acquired immunodeficiency syndrome (AIDS).<br>13. Evidence of interstitial lung disease or active, non-infectious pneumonitis.
**STATISTICAL CONSIDER ATIONS**	Our sample size is based on the confidence interval width of the positive predictive value (PPV) of clinical complete response to treatment. For full details, please see the Statistical Methods section.
**TOTAL NUMBER OF SUBJECTS**	N = 76
**ESTIMATED ENROLLMENT PERIOD**	Estimated 30 months
**ESTIMATED STUDY DURATION**	Estimated 48 months

TABLE OF CONTENTS

Schema **8**
1. Background and Rationale **9**
2. Study Objectives and Endpoints **17**
3. Eligibility Criteria **18**
4. Subject Registration **21**
5. Treatment Plan **21**
6. Dose Delays/Modifications **25**
7. Study Calendar & Evaluations **34**
8. Biospecimen Studies and Procedures **43**
9. Criteria for Disease Evaluation **45**
10. Drug Information **46**
11 Adverse Events **49**
12 Statistical Methods **53**
13 Trial Management **56**
14. Data Handling and Record Keeping **58**
15 Ethics **59**
16 References **60**

SCHEMA

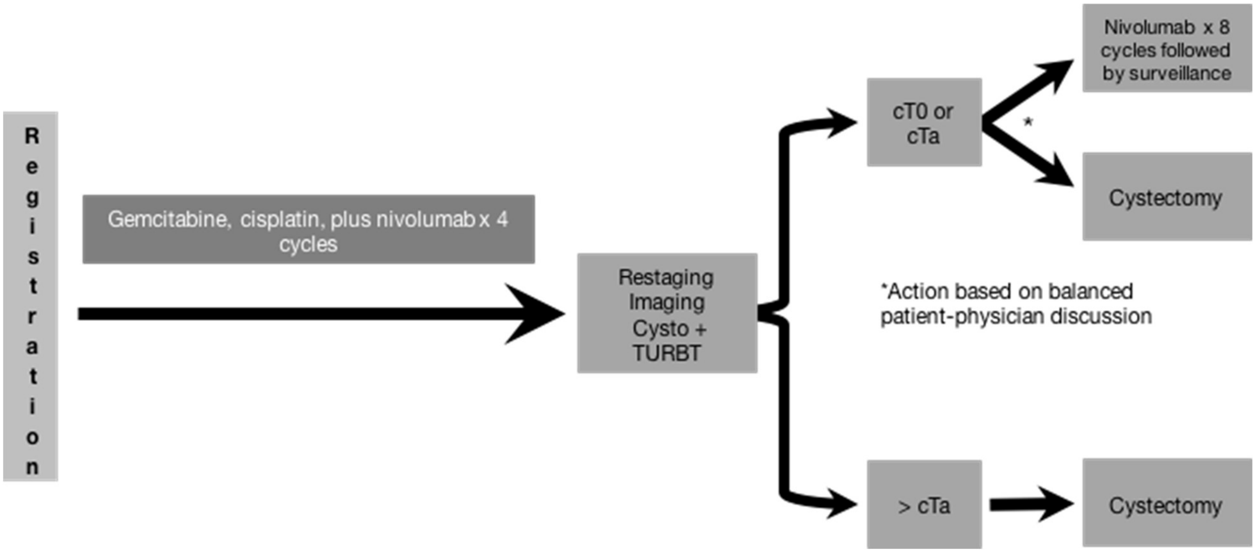

For baseline and restaging imaging studies after gemcitabine + cisplatin + nivolumab, an MRI abdomen and pelvis should be performed (see Table 7) unless contraindicated in which case a CT can be performed

1. BACKGROUND AND RATIONALE

1.1 Standard treatment of muscle-invasive bladder cancer

There are approximately 77,000 new cases of bladder cancer diagnosed each year in the United States. Approximately 30% of patients present with muscle-invasive bladder cancer (MIBC) without evidence of distant metastatic disease. Standard treatment for such patients is radical cystectomy; however, this approach is associated with a life-changing need for urinary diversion and a 90-day mortality rate of ~2-10%.[1–4] Furthermore, despite being performed with curative intent, approximately 50% of patients still develop lethal metastatic recurrence.[5] Accordingly, population-based data demonstrate that a large subset of patients with MIBC in the United States do not undergo radical cystectomy.[6]

In an attempt to improve outcomes, randomized trials have explored the integration of neoadjuvant cisplatin-based chemotherapy. Two randomized trials and a meta-analysis have demonstrated an improvement in survival with the use of neoadjuvant chemotherapy with an absolute survival benefit of approximately 5-10%.[7–9]

Radiation therapy, with or without concurrent chemoradiation, also represents a potential treatment options for MIBC. However, such treatment is associated with grade 3-4 toxicities in 20-30% of patients, late grade ≥ 3 pelvic toxicity in approximately 7% of patients, and the need for salvage cystectomy in 15-30% of patients.[10]

Together, these findings highlight the need for novel bladder-sparing treatment strategies for MIBC.

1.2 A large subset of patients with MIBC treated with neoadjuvant chemotherapy achieve a pathologic complete response

After patients undergo treatment with neoadjuvant chemotherapy followed by radical cystectomy, the bladder specimen is assessed for pathologic evidence of residual cancer. Important, in a subset of patients treated with neoadjuvant chemotherapy, there is no pathologic evidence of residual disease (i.e., pathologic complete response or pCR) and this finding has been associated with improved long term clinical outcomes. For example, in the Southwest Oncology Group 8710 trial comparing cystectomy alone versus 3 cycles of MVAC (methotrexate, vinblastine, doxorubicin, plus cisplatin) followed by cystectomy, 38% of patients treated with neoadjuvant chemotherapy achieved a pCR. The median survival of patients treated with neoadjuvant chemotherapy followed by cystectomy achieving a pCR was not reached compared with 3.8 years in patients with residual disease in the cystectomy specimen.[7] Several other analyses have confirmed that achieving a pCR is highly correlated with improved recurrence-free survival and overall survival (Table 1).[11]

Table 1. Pooled analysis of Nordic neoadjuvant studies demonstrating impact of pCR on outcomes according to clinical T stage (N=449; adapted from Rosenblatt et al[11])

Clinical T stage	5-year survival rate Neoadjuvant arm	5-year survival rate Control arm	p value
All	88.2	57.1	0.001
T2	86.2	59.1	0.023
T3	90.5	50	0.023
T4	100	–	

1.3 Systemic chemotherapy alone as definitive treatment for patients with MIBC

Because neoadjuvant cisplatin-based chemotherapy achieves a pCR in approximately 30-40% of patients with MIBC, a logical question is why this subset of patients requires surgical removal of the bladder (in which case the resected bladder specimen ultimately reveals no evidence of cancer)? The use of transurethral resection of bladder tumor (TURBT) plus systemic chemotherapy alone for the treatment of MIBC is not a new concept. In fact, this approach has been explored since the early 1990's.[12–15] In a series from Herr et al (Table 2), patients achieving a clinical CR (i.e., no evidence of disease on post-chemotherapy cystoscopy, TURBT, and CT scan) with chemotherapy and not undergoing cystectomy demonstrated similar rates of long term survival to patients undergoing immediate cystectomy.[13]

Table 2. Outcome of patients who achieved clinical T0N0M0 status after Neoadjuvant MVAC Chemotherapy (n=60) (adapted from Herr at al[13])

Post-MVAC Treatment	No. of Patients	Patients with Invasive Local Relapse		10-Year Survival			
				Overall		Bladder Intact	
		No.	%	No.	%	No.	%
No cystectomy	28	8	29	21	75	17	61
Partial cystectomy	15	5	33	11	73	8	53
Radical cystectomy	17	0	0	11	65	0	0

The body of literature describing the role of TURBT plus systemic therapy was systematically and comprehensively reviewed by Moran and colleagues in 2017.[16] This systematic review and meta-analysis revealed key findings highlighting the potential benefit of this strategy and the need for further study. These key findings are summarized below:

- Much of the literature describing chemotherapy plus TURBT as definitive management of MIBC is based on retrospective series and/or single center series.
- Prior studies of this approach have included a mix of approaches including the use of carboplatin-based chemotherapy, lack of post-treatment TURBT, and offering surveillance to patients even without a clinical complete response to treatment.
- Meta-analysis of these studies revealed an estimated 5-year survival rate of 72%.
- There have only been two contemporary *prospective* studies exploring chemotherapy plus

TURBT as definitive management for MIBC (described in further detail below). Despite the use of carboplatin-based chemotherapy in Southwest Oncology Group (SWOG) 0219 in all patients, and in the Solsona trial in a subset of patients, both studies demonstrated similar survival rates in patients opting for surveillance after chemotherapy versus cystectomy.

1.3.1 SWOG 0219

Southwest Oncology Group trial 0219 enrolled 74 patients with MIBC who received chemotherapy with a regimen of paclitaxel, gemcitabine, plus carboplatin.[14] Patients achieving a clinical CR to chemotherapy on this study could elect immediate cystectomy or proceed with cystoscopic surveillance alone (those without a clinical CR were to undergo immediate cystectomy). A clinical CR was achieved in 34 of 74 patients (46%). Of the 34 patients with a clinical CR, 10 underwent immediate cystectomy, 6 of whom had pathologic evidence of residual cancer. With a median follow-up of 22 months the Kaplan-Meier estimate of 2-year survival for the entire study population was 59% (95% CI 45, 72). Among the 34 patients with a clinical CR after chemotherapy, the 2-year survival estimate was 75% (95% CI 57, 93). Of the 24 patients with clinical CR who did not undergo cystectomy, the 2-year overall survival estimate was 76%. Of the 10 patients with a clinical CR who underwent cystectomy, the 2-year overall survival estimate was 70%. While long term outcomes were similar among the small subgroups of patients achieving clinical T0 status undergoing immediate cystectomy versus cystoscopic surveillance alone, the disconnect between clinical and pathologic CR status led the authors to conclude that definitive cystectomy after completion of chemotherapy should still be strongly considered. However, this study utilized a carboplatin-based regimen and cisplatin-based chemotherapy is standard neoadjuvant chemotherapy in MIBC and has been associated with higher complete response rates.[17] Further, this interpretation minimizes the potential role of salvage cystectomy.

Indeed, these considerations are exemplified in a recent retrospective series describing the outcomes of patients with MIBC achieving a clinical CR to cisplatin-based chemotherapy.[18] A series from Meyer et. al. included 109 patients treated at a single institution, 32 of whom achieved a clinical CR with neoadjuvant chemotherapy. Of 25/32 patients who declined radical cystectomy after achieving a clinical CR, 7 experienced local relapse during follow-up and proceeded to radical cystectomy. In the 25/32 patients who elected bladder preservation after achieving a clinical CR with neoadjuvant chemotherapy, the 5-year cancer specific survival was 88%.

1.3.2 Solsona trial

Solsona and colleagues enrolled 146 patients with MIBC, with persistent microscopic evidence of disease on re-staging TURBT, in a prospective trial.[19] Patients enrolled in this study could elect to proceed with radical cystectomy versus a bladder-sparing approach involving systemic chemotherapy alone. Systemic therapy was comprised of combination chemotherapy which evolved during the course of the study and could include carboplatin-based therapy in patients with impaired renal function. In this study, 75 patients opted for TURBT + chemotherapy and 71 patients opted for radical cystectomy and despite the lack of randomization, there was no significant difference between important covariates in these groups. Of the patients opting for the bladder-sparing approach, the cancer specific survival and progression-free survival with bladder

preserved at 5 years were 64.5% and 59.8% respectively, and at 10 years were 52.6% and 34.5%, respectively. Comparing the cancer specific survival of the bladder-sparing and radical cystectomy cohorts, there was no significant difference between them. Though this study differs in important ways from other TURBT plus chemotherapy approaches as definitive management of MIBC (i.e., the decision to pursue bladder-sparing was made prior to initiation of chemotherapy and there was no risk stratification based on clinical response to chemotherapy), comparable outcomes were still observed relative to the cystectomy cohort.

Together, the collective literature evaluating TURBT plus systemic therapy for the management of MIBC demonstrates the feasibility of this approach and relatively similar longer term outcomes based on non-randomized comparisons with patients underoing immediate cystectomy. However, aditional prospective studies are required as are improved systemic therapy approaches and potential predictive biomarkers to fully realize this potential paradigm shift in MIBC care.

1.4 Somatic mutations in DNA damage response genes, and high somatic mutational load, are associated with a high likelihood of achieving a pCR with neoadjuvant cisplatin-based chemotherapy

High throughput comprehensive genomic analyses of solid tumors, including MIBC, have uncovered recurrent somatic alterations providing clues to disease pathogenesis and identifying potential therapeutic targets. Somatic alterations in genes encoding proteins involved in DNA damage response and repair are relatively common in MIBC. Indeed, analysis of data from 389 urothelial cancer specimens from The Cancer Genome Atlas reveals deleterious (nonsense, frameshift, splice site, or hotspot missense) alterations in DNA damage response genes (DDR) in approximately ~28% of bladder cancers. Because computational predictions, and functional analyses, of many such alterations suggest these alterations result in loss of function, this raises the hypothesis that cancer cells with faulty DNA damage response and repair machinery may be particularly susceptible to DNA-damaging chemotherapy. Indeed, at least four studies have now demonstrated that the presence of somatic mutations in DDR genes correlates with an increased likelihood of achieving a pCR to cisplatin-based combination chemotherapy in patients with MIBC.[20–23] In an analysis of a cohort of 50 patients with MIBC (25 patients achieving a pCR and 25 patients not achieving a pCR with neoadjuvant chemotherapy), Rosenberg and colleagues performed whole exome sequencing on the pre-chemotherapy transurethral resection of bladder tumor (TURBT) specimens and demonstrated that the presence of mutations in *ERCC2* were found exclusively in the patients achieving a pCR.[21] *ERCC2* encodes a nucleotide excision repair protein and is mutated in ~12% of MIBCs. The correlation between *ERCC2* mutations and pCR was subsequently validated in an independent cohort.[22] Plimack et al performed targeted exome sequencing on a cohort of patients with MIBC treated with dose-dense MVAC.[23] These investigators demonstrated that alterations in one or more of three DDR genes (ATM, RB1, and FANCC) predicted pCR ($p<0.001$, 87% sensitivity, 100% specificity) and better overall survival ($p=0.007$). Notably, in both the studies from Rosenberg and Plimack, higher somatic mutational load was also associated with an increased likelihood of achieving a pCR. Iyer and colleagues performed targeted exome sequencing on a cohort of patients with MIBC treated on a phase 2 trial of neoadjuvant dose-dense gemcitabine plus cisplatin.[20] Mutations in a set of 29 DDR genes were correlated with pathologic response revealing that deleterious DDR gene mutations were associated with achieving a pCR with 92% specificity and a positive predictive value of 88%.

The performance characteristics of ATM, RB1, FANCC, and ERCC2 for predicting pCR with cisplatin-based chemotherapy are shown in Table 3. Together, these studies suggest that the presence of deleterious somatic DDR alterations, or high somatic mutational load, may aid in the identification of patients for bladder-sparing with TURBT plus systemic chemotherapy. However, the relatively poor sensitivity of these biomarkers indicates that this approach, if validated, would only identify a subset of patients suitable for this approach. Further validation and additional biomarker discovery is therefore indicated.

Table 3. Studies correlating DDR mutations with response to neoadjuvant chemotherapy

Gene(s)/Cohort	N	Sensitivity	Specificity	PPV	NPV
ATM, RB1, FANCC					
Discovery	34	87%	100%	100%	90%
Validation	24	64%	85%	78%	73%
ERCC2					
Discovery	50	36%	100%	100%	61%
Validation	48	80%	93%	80%	93%

Derived from Van Allen et al, Cancer Discovery, 2014; Plimack et al, European Urology, 2015; Liu et al, JAMA Oncology, 2016
PPV, positive predictive value; NPV, negative predictive value

1.5 DDR mutations may also increase the likelihood of response to immune checkpoint blockade

Though several putative predictive biomarkers of response to PD-1/PD-L1 blockade have been explored in clinical trials to date, mutational load (i.e., the number of non-synonymous mutations per tumor specimen) has been consistently demonstrated to correlate with response across several solid tumors including urothelial cancer of the bladder.[24,25] A higher mutational load is postulated to lead to a higher number of potential neoantigens thereby increasing the probability of developing an antitumor immune response.[26,27] In a prior study of patients with MIBC undergoing cystectomy, recurrence-free survival was significantly better in patients with DDR mutations, independent of the use of perioperative chemotherapy.[28] These findings raised the hypothesis that the improvement in outcomes associated with DDR mutations was the result of faulty DNA repair leading to an increase in mutational burden, neoantigen expression, and ultimately an antitumor immune response. Indeed, DNA mismatch repair gene mutations in colorectal and other cancers have been correlated with a particularly high response rate to immune checkpoint blockade.[29] However, the association between other DDR pathway gene mutations (e.g., homologous recombination, nucleotide excision repair, etc) and both mutational load and response to immune checkpoint blockade has been underexplored. To explore this notion, we utilized three cohorts: (1) TCGA MIBC cohort (n = 389), (2) Mount Sinai (MS) cohort (n = 67) of bladder cancer (cystectomy) specimens subjected to targeted exome sequencing for 341 genes (MSK-IMPACT), and (3) Phase 2 trial of gemcitabine, cisplatin, plus CTLA-4 blockade with ipilimumab (GCI) in metastatic urothelial cancer from which 28/36 enrolled patients had specimens suitable for whole exome sequencing. Deleterious mutations in

DDR genes were defined as nonsense, frameshift, splice site, or hotspot point mutations. Notably, the mutational load using all genes in the TCGA cohort, and restricted to the 341 IMPACT genes, were highly correlated (rs= 0.81, p < 0.001). Associations between deleterious DDR mutations and mutational load are shown in Table 4. In the MS cohort, CD8+cells/mm^2 by IHC were higher in tumors with deleterious DDR mutations versus no DDR mutations (p = 0.04). In the GCI cohort, the sensitivity, specificity, positive predictive value, and negative predictive value of a deleterious DDR mutation for objective response to treatment was 40.9% (95% CI 20.7-63.7%), 86.7% (95% CI 42.1-99.4%), 90% (55.5-99.8%), and 31.6% (95% CI 12.6-56.6%), respectively. Notably, 2 of 9 patients with deleterious DDR mutations, and with the highest mutational loads, achieved complete responses after GCI and are alive without evidence of disease at 2.1+ and 1.8+ years. These findings demonstrate that DDR mutations are associated with higher mutational load in bladder cancer, a high likelihood of response to chemotherapy plus immune checkpoint blockade, and may identify a subset of patients with metastatic urothelial bladder cancer achieving durable disease control. Further, these findings support exploring cisplatin-based chemotherapy with more efficacious immune checkpoint blockade in bladder cancer (i.e., PD-1 blockade) in patients with DDR-mutant, or high mutational load harboring, bladder cancer.

Table 4. Association between deleterious DDR mutations and mutational load in independent datasets

Cohort	N	Deleterious DDR mutation	Mut Load SNV/tumor, median (range)		p
			Deleterious DDR mutation	Other	
TCGA	389	28%	271 (26-3617)	148 (22-1016)	<0.001
MS	67	21%	31 (17-66)	18 (9-42)	<0.001
GCI	28	32%	344 (101-4292)	232 (84-1010)	0.027

Finally, the association between DDR mutations and response to PD-1/PD-L1 blockade was reported by Teo and colleagues.[30] Sixty patients with urothelial cancer enrolled in prospective trials of anti-PD-1/PD-L1 antibodies were analyzed. Any DDR and known or likely deleterious DDR mutations were identified in 28 (47%) and 15 (25%) patients, respectively. The presence of any DDR alteration was associated with a higher response rate (67.9% v 18.8%; P < .001). A higher response rate was observed in patients whose tumors harbored known or likely deleterious DDR alterations (80%) compared with DDR alterations of unknown significance (54%) and in those whose tumors were wild-type for DDR genes (19%; P < .001). DDR alterations also were associated with longer progression-free and overall survival.

Together, these findings indicate that the presence of DDR mutations might predict response to cisplatin-based chemotherapy and PD-1/PD-L1 blockade independently and might identify patients particularly sensitive to combination therapy.

1.6 Nivolumab

Nivolumab (BMS-936558 or MDX1106) is a fully human monoclonal immunoglobulin (Ig) G4 antibody that binds to the programmed cell death receptor-1 (PD-1), a negative regulatory molecule expressed by activated T and B lymphocytes. Inhibition of the interaction between PD-

1 and its ligands promote immune responses and antigen-specific T cell responses to both foreign and self antigens.

Nivolumab has demonstrated clinical activity as monotherapy, and as combination therapy in several tumor types including renal cell carcinoma, melanoma, non-small cell lung cancer, urothelial cancer, and some lymphomas. The majority of responses have been durable and exceeded 6 months. The overall safety experience with nivolumab, as a monotherapy or in combination with other therapeutics, is based on experience in approximately 4,000 subjects treated to date. For nivolumab monotherapy, the safety profile has been similar across tumor types.

1.7 Nivolumab in urothelial cancer

The safety and activity of single-agent nivolumab advanced urothelial cancer has been explored in two prospective clinical trials to date. CheckMate 032 was a phase 1/2 study enrolling 86 patients to treatment with nivolumab 3 mg/kg every 2 weeks; 78 received at least one dose of treatment.[31] A confirmed objective response was achieved in 19 (24.4%, 95% CI 15.3-35.4) of 78 patients and the vast majority of responses were durable. Based on these results, a large phase II trial was initiated to confirm the safety and activity of nivolumab in metastatic urothelial cancer. CheckMate 275 enrolled 270 patients with metastatic urothelial cancer that had progressed despite prior platinum-based chemotherapy.[32] With a minimum follow-up of 7 months, the objective response rate was 19.6% (95% CI 15.0-24.9%). While higher tumor PD-L1 expression (as measured by immunohistochemistry) on tumor cells was associated with a numerically higher objective response rate, responses were seen even in patients with PD-L1 expression < 1% [objective response rate = 16.1% (95% CI 10.5–23.1]. Responses occurred rapidly [median time to response = 1.9 months (1.6–5.9)] and were durable; at a median follow-up of 7 months, 77% of responses were ongoing. The safety profile of nivolumab was consistent with what has been observed across indications (Table 5 and 6).

Table 5. Treatment-related adverse events in ≥5% of patients (n=270)

Event	% Any Grade	%Grade 3–4
**All treatment-related adverse events**	64.4	17.8
Fatigue	16.7	1.9
Pruritus	9.3	0
Diarrhea	8.9	1.9
Decreased appetite	8.1	0
Hypothyroidism	7.8	0
Nausea	7.0	<1
Asthenia	5.9	1.5
Rash	5.9	1.1
Pyrexia	5.6	0
Treatment-related adverse events leading to discontinuation	4.8	3.0

Table 6. Immune mediated adverse events (n=270)

Events	%, Any Grade	%, Grade 3–4
**Skin**	17.4	1.5
Rash	5.9	1.1
**Endocrine**	14.4	<1
Hypothyroidism	7.8	0
**Gastrointestinal**	9.3	2.2
Diarrhea	8.9	1.9
Colitis	<1	<1
**Pulmonary**	4.1	1.1
Pneumonitis	3.7	<1
**Hepatic**	3.7	1.9
Elevated ALT enzymes	3.0	<1
Elevated AST enzymes	2.2	1.1
**Renal**	1.1	<1

Given the experience with nivolumab in patients with metastatic urothelial cancer, the Food and Drug Administration approval of atezolizumab (PD-L1 antibody) for the treatment of patients with platinum-resistant metastatic urothelial cancer[24], and the demonstration of an improvement in survival with pembrolizumab (PD-1 antibody) compared with chemotherapy in patients with platinum-resistant metastatic urothelial cancer, exploring PD-1/PD-L1 blockade in earlier bladder cancer disease states is warranted.

1.8 Combining nivolumab with chemotherapy

An expansion of CheckMate 012 study explored nivolumab in combination with platinum-based chemotherapy in patients with advanced non-small cell lung cancer including combination regimens with gemcitabine plus cisplatin, paclitaxel plus carboplatin, or pemetrexed plus cisplatin (n= 56).[33] The safety profile of nivolumab plus platinum-based chemotherapy was consistent with that expected for the individual agents. A second trial explored the combination of nivolumab 10 mg/kg + gemcitabine plus cisplatin, pemetrexed plus cisplatin, paclitaxel plus carboplatin plus bevacizumab or docetaxel (n=24) in patients with advanced non-small cell lung cancer.[34] This trial demonstrated similar findings with no new safety signals observed with the combination regimens.

1.9 Rationale for current trial

This is a phase 2 trial seeking to define the safety and activity of gemcitabine, cisplatin, plus nivolumab as neoadjuvant therapy in patients with muscle-invasive bladder cancer and to determine the potential role of a clinical complete response in identifying patients who can be definitively managed without cystectomy.

The current study is supported by several lines of evidence

1. Radical cystectomy is potentially curative for patients with MIBC but is a life-changing operation and achieves cure in only 50% of unselected patients. Further, the risk of mortality within 90-days of cystectomy in the United States is approximately 8%.[4]
2. Neoadjuvant chemotherapy results in an absolute survival improvement of approximately 5-10%. However, patients treated with neoadjuvant chemotherapy achieving a pCR have markedly improved outcomes.
3. TURBT plus chemotherapy as definitive management for MIBC, in patients achieving a clinical CR, has been shown to be feasible, leads to a relatively high rate of bladder-intact disease-free and overall survival, and achieves outcomes comparable immediate cystectomy in non-randomized studies. However, there have been a paucity of prospective studies seeking to refine this approach.
4. Chemotherapy and PD-1 blockade are non-cross resistant in urothelial cancer and are theoretically synergistic based on mechanistic considerations. Such combinations have already been shown to improves outcomes in other solid tumors (e.g., non-small cell lung cancer). Therfore, TURBT plus chemotherapy plus PD-1 blockade may represent a novel paradigm-shifting curative bladder sparing approach to patients with MIBC achieving a clinical complete response to treatment.
5. Genomic biomarkers may allow refinement of this approach and warrant further investigation.

2. STUDY OBJECTIVES AND ENDPOINTS

2.1 Objectives

2.1.1 Primary Objective

- Determine the clinical complete response rate (cT0 or cTa) with gemcitabine, cisplatin, plus nivolumab
- Determine the ability of clinical complete response (cT0 or cTa) to predict benefit from treatment. Benefit will be defined as a pathologic complete response (<pT1) in patients undergoing cystectomy and 2 year metastasis-free in patients pursuing surveillance

2.1.2 Secondary Objectives

- Determine the safety of neoadjuvant gemcitabine, cisplatin, plus nivolumab
- Describe the proportion of patients with a clinical complete response to pursue cystectomy versus surveillance
- Determine the association between a prespecified panel of genomic biomarkers and benefit from treatment in patients achieving a clinical complete response. Benefit will be defined as a pathologic complete response (p<T1) in patients undergoing cystectomy and 2 years metastasis-free in patients pursuing surveillance
- Determine the pathologic complete response rate (<pT1) in patients pursuing cystectomy
- Determine the 2-year metastasis free survival.
- Determine overall survival (and bladder-intact overall survival rate)
- Determine the recurrence-free survival

2.1.3 Exploratory Objectives

- Explore the association of immune biomarkers, genomic alterations, circulating tumor DNA, gene expression, and radiographic characteristics (including radiomics) with clinical complete response rate, <pT1 rate (where applicable), recurrence-free survival, the bladder-intact overall survival, and overall survival.
- Explore potential genomic and immunologic biomarkers in the urine and blood that correlate with treatment benefit

2.2 Endpoints

2.2.1 Primary Endpoint

- Clinical complete response rate will be defined as cT0 or cTa disease after gemcitabine, cisplatin, plus nivolumab. Benefit from treatment will be defined as a pathologic complete response (<pT1) in patients undergoing cystectomy and 2 years metastasis-free in patients proceeding with surveillance.

2.2.2 Secondary Endpoints

- Safety will be determined according to the NCI Common Terminology Criteria for Adverse Events (NCI CTCAE) v4.03
- Bladder-intact overall survival is defined as the time from initiation of treatment until death or cystectomy
- Recurrence-free survival is defined as the time from initiation of treatment to death or recurrence, depending on which occurs first
- Overall survival is defined as the time from initiation of treatment to death
- Pathologic complete response rate in patients undergoing radical cystectomy is defined as the proportion of patients with <pT1
- Positive predictive value of a panel of genomic alterations in predicting benefit from treatment

3. ELIGIBILITY CRITERIA

3.1 Inclusion Criteria

Subject must meet all the following applicable inclusion criteria to participate in this study:

1. Written informed consent and HIPAA authorization for release of personal health information prior to registration. **NOTE:** HIPAA authorization may be included in the informed consent or obtained separately.

2. Age ≥ 18 years at the time of consent.

3. ECOG Performance Status of ≤ 1 within 28 days prior to registration.

4. Histological evidence of clinically localized muscle-invasive urothelial cancer of the bladder (i.e., ct2-4n0m0). candidate for cystectomy as per treating physician.

5. Demonstrate adequate organ function as defined in the table below. All screening labs to be obtained within 28 days prior to registration.

**System**	**Laboratory Value**
**Hematological**	
Absolute Neutrophil Count (ANC)	≥ 1.5 x 10^9/L
Hemoglobin (Hgb)	≥ 9 g/dL
Platelets	≥ 100 x 10^9/L
**Renal**	
Calculated creatinine clearance[1]	Creatinine ≤ 1.5 or creatinine clearance ≥ 60 mL/min
**Hepatic**	
Bilirubin	≤ 1.5 × upper limit of normal (ULN) (except subjects with Gilbert Syndrome, who can have total bilirubin < 3.0 mg/dL)
Aspartate aminotransferase (AST)	≤ 3 × ULN
Alanine aminotransferase (ALT)	≤ 3 × ULN

1: Cockcroft-Gault formula will be used to calculate creatinine clearance

6. All subjects must have adequate archival tissue identified during screening (i.e., at least 15 unstained slides or paraffin block). Subjects without available archival tissue must be discussed with the sponsor-investigator.

7. Women of childbearing potential must have a negative serum or urine pregnancy within 7 days prior to C1D1. **NOTE:** **"**Women of childbearing potential" is defined as any female who has experienced menarche and who has not undergone surgical sterilization (hysterectomy or bilateral oophorectomy) or who is not postmenopausal. Menopause is defined clinically as 12 months of amenorrhea in a woman over 45 in the absence of other biological or physiological causes. In addition, women under the age of 62 must have a documented serum follicle stimulating hormone (FSH) level less than 40 mIU/mL.

   NOTE: Women of childbearing potential (WOCBP) receiving nivolumab must be willing to abstain from heterosexual intercourse or to use 2 forms of effective methods of contraception from the time of informed consent to 5 months after the last dose of nivolumab or for the timeframe outlined per package insert for chemotherapy. This timeframe also applies to breastfeeding. The two contraception methods can be comprised of two barrier methods, or a barrier method plus a hormonal method.

   Male subjects capable of fathering a child that are sexually active with partners of childbearing potential must be willing to abstain from heterosexual intercourse or to use 2 forms of effective methods of contraception from the time of informed consent to the timeframe outlined per package insert for chemotherapy. Contraception is not required for nivolumab. The timeframes described in the previous 2 sentences apply to sperm donation. Two contraception methods can be comprised of two barrier methods, or a barrier method plus a hormonal method.

HIGHLY EFFECTIVE METHODS OF CONTRACEPTION

- Male condoms with spermicide
- Hormonal methods of contraception including combined oral contraceptive pills, vaginal ring, injectables, implants and intrauterine devices (IUDs) such as Mirena® by WOCBP subject.
- Nonhormonal IUDs, such as ParaGard®
- Tubal ligation
- Vasectomy
- Complete Abstinence which is defined as complete avoidance of heterosexual intercourse and is an acceptable form of contraception for all study drugs.

LESS EFFECTIVE METHODS OF CONTRACEPTION

- Diaphragm with spermicide
- Cervical cap with spermicide
- Vaginal sponge
- Male Condom without spermicide
- Female Condom. A male and female condom must not be used together
- Progestin only pills by WOCBP subject

3.2 Exclusion Criteria

Subjects meeting any of the criteria below may not participate in the study:

1. No prior systemic chemotherapy for muscle-invasive urothelial cancer of the bladder

2. Active infection requiring systemic therapy

3. Pregnant or breastfeeding (**NOTE:** breast milk cannot be stored for future use while the mother is being treated on study).

4. Any serious or uncontrolled medical disorder that, in the opinion of the investigator, may increase the risk associated with study participation or study drug administration, impair the ability of the subject to receive protocol therapy, or interfere with the interpretation of study results.

5. Prior malignancy active within the previous 3 years except for locally curable cancers that have been apparently cured.

6. Subjects with active, known or suspected autoimmune disease. Subjects with vitiligo, type I diabetes mellitus, residual hypothyroidism due to autoimmune condition only requiring hormone replacement, psoriasis not requiring systemic treatment, or conditions not expected to recur in the absence of an external trigger are permitted to enroll.

7. Subjects with a condition requiring systemic treatment with either corticosteroids (> 10 mg daily prednisone equivalents) or other immunosuppressive medications within 14 days of study drug administration. Inhaled or topical steroids and adrenal replacement

doses > 10 mg daily prednisone equivalents are permitted in the absence of active autoimmune disease.

8. Prior treatment with an anti-PD-1, anti-PD-L1, anti-PD-L2, anti-CTLA-4 antibody, or any other antibody or drug specifically targeting T-cell co-stimulation or immune checkpoint pathways.

9. Grade ≥ 2 neuropathy (NCI CTCAE v4).

10. Prior radiation therapy for bladder cancer

11. Known history of positive test for hepatitis B virus surface antigen (HBV sAg) or hepatitis C virus ribonucleic acid (RNA) or hepatitis C antibody (HCV antibody) indicating acute or chronic infection. Testing not required.

12. Known history of testing positive for human immunodeficiency virus (HIV) or known acquired immunodeficiency syndrome (AIDS). Testing not required

13. Evidence of interstitial lung disease or active, non-infectious pneumonitis.

14. Solid organ or allogeneic stem cell transplant

4. SUBJECT REGISTRATION

All subjects must be registered through HCRN's electronic data capture (EDC) system. A subject is considered registered when an "on study" date is entered into the EDC system.

Subjects must be registered prior to starting protocol therapy. Subjects must begin study treatment **within 7 business days** of registration.

5. TREATMENT PLAN

5.1 Treatment Overview

Subjects with cT2-T4aN0M0 urothelial cancer of the bladder will be enrolled. After completing four cycles of neoadjuvant gemcitabine, cisplatin, plus nivolumab, subjects will undergo a restaging MRI of the abdomen and pelvis (and CT chest). A CT of the abdomen and pelvis may be performed if there are contraindications for MRI. Patients will also undergo a restaging cystoscopy and biopsies/TURBT. If there is evidence of gross residual disease at cystoscopy, biopsies/TURBT may be performed at the discretion of the treating physician but such patients will be considered as having not achieved a clinical complete response and will proceed with cystectomy.

Patients achieving a clinical complete response to treatment (defined in section 9.1) may opt to proceed with cystectomy or may opt to proceed with an additional 8 cycles of single agent nivolumab followed by surveillance. This decision should be based on a balanced discussed with the treating physician and documented in the medical record. Patients opting for surveillance will

sign a separate consent form outlining the potential risks, benefits, and knowledge gaps regarding this approach.

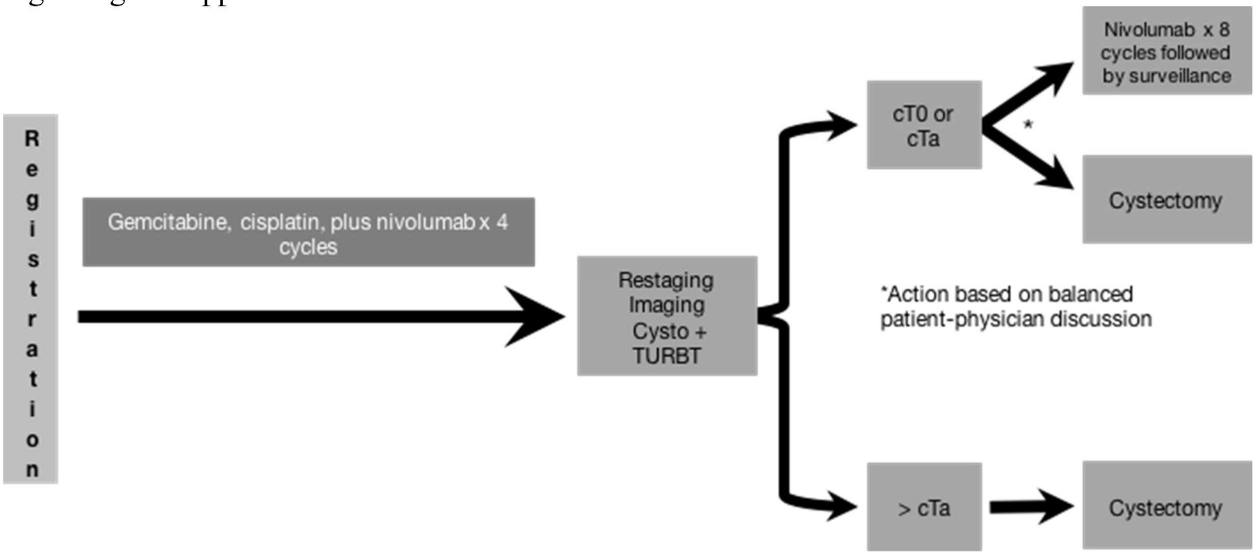

For restaging imaging studies after gemcitabine + cisplatin + nivolumab, an MRI abdomen and pelvis should be performed unless contraindicated – the preferred MRI protocol is outlined in Table 7.

5.2 Pre-Medication Guidelines

5.2.1 Gemcitabine

There are no required/recommended premedications for the administration of gemcitabine. Antiemetics may be administered per institutional guidelines.

5.2.2 Cisplatin

The antiemetic regimen is at the discretion of the site investigator and according to institutional guidelines. However, non-steroid containing pre-medication regimens are preferred when possible given the incompletely defined impact of such medications on response to immune checkpoint blockade.

5.2.3 Nivolumab

There are no required premedications for the administration of nivolumab.

5.3 Medication Administration

Cycles 1-4: Gemcitabine, Cisplatin, plus Nivolumab

Agent	Route	Dose	Administration Time	Frequency * (± 2 days)	Cycle Length	Total # Cycles
Nivolumab	IV	360 mg	30 minutes	Day 1	21 days	4 cycles
Gemcitabine	IV	1000 mg/m^2	Per institutional guidelines	Day 1, 8		

Cisplatin	IV	70 mg/m^2	Per institutional guidelines	Day 1		

Cycles 5-12: Maintenance Single Agent Nivolumab (starting ~ 2-6 weeks after completing Cycle 4 of gemcitabine, cisplatin, plus nivolumab) [only in patients achieving cT0 or cTa status on restaging after 4 cycles of gemcitabine, cisplatin, plus nivolumab and opting to proceed without cystectomy]

Agent	Route	Dose	Administration Time	Frequency * (± 2 days)	Cycle Length	Total # Cycles
Nivolumab	IV	240 mg	30 minutes	Day 1	14 days	8 cycles

NOTE: * Infusions may be given ± 2 days for reasons such as observed holidays, inclement weather, scheduling conflicts, etc. It should be clearly documented in patient's chart and case report forms. During Cycles 1-4, nivolumab should be administered first followed by gemcitabine then cisplatin. An infusion window of – 5 minutes and + 10 minutes may be applied.

The body surface area and chemotherapy drug dose should be recalculated ONLY if the patient's weight changes by > 10% during the course of the study.

5.4 Concomitant Medications

5.4.1 Allowed Concomitant Medications

Medications specifically prohibited in the exclusion criteria are not allowed during the ongoing trial. If there is a clinical indication for any medication specifically prohibited during the trial, discontinuation from trial therapy or vaccination may be required. The site investigator should discuss any questions regarding this with the sponsor-investigator. The final decision on any supportive therapy rests with the site investigator and/or the subject's primary physician. However, the decision to continue the subject on trial therapy schedule requires the mutual agreement of the site investigator, the sponsor-investigator and the subject.

- All treatments that the site investigator considers necessary for a subject's welfare may be administered at the discretion of the treating investigator in keeping with the community standards of medical care.
- All concomitant medications received within 30 days before the first dose of trial treatment and 30 days after the last dose of trial treatment should be recorded.
- Concomitant therapy includes any prescription medications or over-the-counter preparations used by a patient between the 7 days preceding the screening evaluation and the treatment discontinuation visit.
- Patients who use oral contraceptives, hormone-replacement therapy, prophylactic or therapeutic anticoagulation therapy (such as low-molecular-weight heparin or warfarin at a stable dose level), or other allowed maintenance therapy should continue their use. Females of reproductive potential should use highly effective means of contraception as outlined in the eligibility.

5.4.2 Prohibited Concomitant Medications

Subjects are prohibited from receiving the following therapies during the Treatment Phase of this trial:

- Antineoplastic systemic chemotherapy or biological therapy not specified in this protocol
- Immunotherapy not specified in this protocol
- Radiation therapy

5.5 Supportive Care

The use of supportive care will be permitted as clinically indicated and according to institutional guidelines. The use of white blood cell and red blood cell growth factors should be consistent with ASCO guidelines. However, growth factors should be not used during Cycle #1 or in lieu of recommended dose reductions.

6. DOSE DELAYS/MODIFICATIONS

The NCI Common Terminology Criteria for Adverse Events (CTCAE) v4.03 will be used to grade adverse events.

Subjects enrolled in this study will be evaluated clinically and with standard laboratory tests before and at regular intervals during their participation in this study as specified in Section 7.

Subjects will be evaluated for adverse events (all grades), serious adverse events, and adverse events requiring study drug interruption or discontinuation at each study visit for the duration of their participation in the study.

6.1 Initiation of New Cycle of Treatment

Dose modifications will be based on blood counts within 3 days prior to Day 1 or Day 8 of each cycle.

Each treatment cycle will begin only when the criteria below and those outlined in Section 6.5 (Dose Delays for Nivolumab) are met:

- ANC $\geq$ 1.5 K/mm^3
- Platelets $\geq$ 100 K/mm^3
- Resolution of non-hematologic treatment-related toxicities to $\leq$ Grade 1 or baseline

Subjects requiring treatment to be held for toxicity > 4 weeks should proceed with definitive management of their tumor (e.g., cystectomy) as appropriate as determined by their treatment physician.

6.2 Treatment Limiting Adverse Event

A treatment-limiting adverse event is any adverse event related to study treatment (includes cisplatin, gemcitabine and nivolumab) experienced during the study resulting in treatment termination.

6.3 Dose Delays/Modifications for Treatment Related Hematological Toxicity

6.3.1 Dose Delays/Modifications for Day 1 of Treatment (Cycle 1 through Cycle 4)

The following dose modifications will be based on blood counts done within 3 days prior to <u>**Day 1**</u> of each Cycle of therapy.

Treatment with nivolumab will also be HELD until patients meet criteria to resume dosing with gemcitabine and cisplatin.

ANC		Platelets	Cisplatin dose	Lowest gemcitabine dose in prior Cycle	Day 1 dose of gemcitabine
≥ 1.5 K/mm^3	And	≥ 100 K/mm^3	Continue dose of cisplatin from prior cycle (eg, if no prior dose reductions, continue at 70 mg/m^2)	1000 mg/m^2	1000 mg/m^2
≥ 1.5 K/mm^3	And	≥ 100 K/mm^3		800 mg/m^2	800 mg/m^2
≥ 1.5 K/mm^3	And	≥ 100 K/mm^3		600 mg/m^2	600 mg/m^2
< 1.5 K/mm^3	Or	< 100 K/mm^3	*Hold and recheck CBC with differential	Any	*Hold and recheck CBC with differential

*Once ANC ≥ 1500 and platelets ≥ 100,000, resume therapy with gemcitabine reduced by 1 dose level. If gemcitabine has already been reduced by 1 dose level, discuss dose reduction with sponsor-investigator. Granulocyte colony stimulating factors may be used at the discretion of the site investigator; however, growth factors should be not used during Cycle #1 or in lieu of recommended dose reductions.

6.3.2 Dose Delays/Modifications for Day 8 Treatment (Cycle 1 through Cycle 4)

The following dose modifications of gemcitabine will be based on blood counts done within 3 days prior to <u>**Day 8**</u> of each Cycle of therapy.

ANC		Platelets	if Day 1 dose level of gemcitabine was:	then Day 8 dose level of gemcitabine will be:
≥ 1.5 K/mm^3	And	≥ 100 K/mm^3	1000 mg/m^2	1000 mg/m^2
≥ 1.5 K/mm^3	And	≥ 100 K/mm^3	800 mg/m^2	800 mg/m^2
≥ 1.5 K/mm^3	And	≥ 100 K/mm^3	600 mg/m^2	600 mg/m^2
1.0 K/mm^3 to 1.4 K/mm^3	And	≥ 100 K/mm^3	1000 mg/m^2	800 mg/m^2
1.0 K/mm^3 to 1.4 K/mm^3	And	≥ 100 K/mm^3	800 mg/m^2	600 mg/m^2
<1.0 K/mm^3	Or	< 100 K/mm^3	Any	Hold and recheck CBC with differential*

*Treatment held on Day 8 should not be made up at a later date; resume next cycle as scheduled with gemcitabine reduced by 1 dose level.

There should be no dose re-escalation after a dose reduction.

6.3.3 Febrile Neutropenia

If febrile neutropenia develops in a given cycle, hold gemcitabine, cisplatin, and nivolumab during febrile neutropenia.

Resume gemcitabine and cisplatin at one dose lower than the dose administered in the last cycle. This dose should be used for all subsequent cycles. The dose of nivolumab will be unchanged. Granulocyte colony stimulating factors may be used at the discretion of the site investigator.

6.4 Dose Delays/Modifications for Other Treatment Related Non-Hematological Toxicity Secondary to Gemcitabine or Cisplatin

Dose delays/reductions for non-hematologic toxicities attributable to gemcitabine or cisplatin (with the exception of alopecia or nausea/vomiting not optimally managed with antiemetics) are outlined in the table below. Only the drugs felt to be contributing to the toxicity per the site investigator should be dose reduced. Patients with treatment-related nausea that is Grade ≥ 2 despite optimal use of antiemetics will be dose reduced by 1 dose level.

If the Day 8 dose of gemcitabine is held for non-hematologic toxicity, the “Day 8” dose of gemcitabine may be administered the following week provided the adverse event was adequately improved or resolved. If the adverse event has not adequately improved or resolved within 2 weeks, the “day 8” dose of gemcitabine should be held for that cycle and treatment should resume (once the adverse event improves/resolves) with the next cycle of treatment.

6.4.1 Dose Modifications for Non-Hematologic Toxicities

**Non-Hematologic toxicity**	**Gemcitabine/Cisplatin**
Grade 0-2	No change
Grade 3	Hold until Grade ≤ 1 and resume treatment reduced by 1 dose level
Grade 4	Hold until Grade ≤ 1 and resume treatment reduced by 1 dose level

6.4.2 Dose Modifications for Gemcitabine and Cisplatin

**Dose level**	**Gemcitabine**	**Cisplatin**
Dose level -1	800 mg/m^2	60 mg/m^2
Dose level -2	600 mg/m^2	50 mg/m^2

Dose re-escalation after a dose reduction for nonhematologic toxicity should not occur without discussion with the sponsor-investigator.

If toxicity is specifically attributable to cisplatin or gemcitabine and warrants discontinuation of that particular agent, patients may be considered for continuation on treatment with cisplatin (or gemcitabine) and nivolumab but this must be discussed with the sponsor-investigator.

6.5 Dose Delays for Nivolumab

Dose reductions or dose escalations are not permitted.

6.5.1 Dose Delay Criteria

Because of the potential for clinically significant nivolumab-related AEs requiring early recognition and prompt intervention, management algorithms have been developed for suspected AEs of selected categories. [see current Investigator Brochure and Appendix A]

Dose delay criteria apply for all drug-related adverse events (regardless of whether or not the event is attributed to nivolumab). All study drugs must be delayed until treatment can resume. In rare circumstances, treatment with gemcitabine plus cisplatin could potentially be continued in the setting of select adverse events related to nivolumab after discussion and approval from the sponsor-investigator.

Nivolumab administration should be delayed for the following

Any Grade ≥ 2 non-skin, drug-related AE, with the following exceptions:

- Grade 2 drug-related fatigue or laboratory abnormalities do not require a treatment delay
- Any Grade 3 skin, drug-related AE
- Any Grade 3 drug-related laboratory abnormality, with the following exceptions for lymphopenia, leukopenia, AST, ALT, total bilirubin, or asymptomatic amylase or lipase:
    - Grade 3 lymphopenia or leukopenia does not require dose delay.
    - If a subject has a baseline AST, ALT, or total bilirubin that is within normal limits, delay dosing for drug-related Grade ≥ 2 toxicity.
    - If a subject has baseline AST, ALT, or total bilirubin within the Grade 1 toxicity range, delay dosing for drug-related Grade ≥ 3 toxicity.
    - Any Grade ≥ 3 drug-related amylase or lipase abnormality that is not associated with symptoms or clinical manifestations of pancreatitis does not require dose delay. The sponsor-investigator should be consulted for such Grade ≥ 3 amylase or lipase abnormalities.

Any AE, laboratory abnormality, or intercurrent illness which, in the judgment of the site investigator, warrants delaying the dose of study medication.

Subjects who require delay of nivolumab should be re-evaluated weekly or more frequently if clinically indicated and resume nivolumab dosing when re-treatment criteria are met.

6.5.2 Criteria to Resume Treatment

Subjects may resume treatment with nivolumab when the drug-related AE(s) resolve to Grade ≤1 or baseline value, with the following exceptions:

- Subjects may resume treatment in the presence of Grade 2 fatigue
- Subjects who have not experienced a Grade 3 drug-related skin AE may resume treatment in the presence of Grade 2 skin toxicity
- Subjects with baseline Grade 1 AST, ALT or total bilirubin who require dose delays for reasons other than a 2-grade shift in AST, ALT or total bilirubin may resume treatment in the presence of Grade 2 AST, ALT OR total bilirubin
- Subjects with combined Grade 2 AST, ALT AND total bilirubin values meeting discontinuation parameters should have treatment permanently discontinued
- Drug-related pulmonary toxicity, diarrhea, or colitis, must have resolved to baseline before treatment is resumed.
- Drug-related endocrinopathies adequately controlled with only physiologic hormone replacement may resume treatment.

If the criteria to resume treatment are met, the subject should restart treatment at the next scheduled timepoint per protocol. However, if the treatment is delayed past the next scheduled timepoint per protocol, the next scheduled timepoint will be delayed until dosing resumes.

If treatment is delayed or interrupted for > 4 weeks, the subject must be permanently discontinued from study therapy, and should proceed with definitive treatment of their primary tumor as per their treating physician, unless otherwise discussed and approved by sponsor-investigator (e.g., in the setting of completion of a steroid taper).

6.6 Management Algorithms

Guidelines for the management of immune related events can be found in the current Investigator Brochure AND in the Appendix A. Site investigators should decide the appropriate source of AE management for each protocol.

Immuno-oncology (I-O) agents are associated with AEs that can differ in severity and duration than AEs caused by other therapeutic classes. Nivolumab is considered an immuno-oncology agent in this protocol. Early recognition and management of AEs associated with immuno-oncology agents may mitigate severe toxicity. Management algorithms have been developed to assist investigators in assessing and managing the following groups of AEs: Gastrointestinal, Renal, Pulmonary, Hepatic, Endocrinopathies, Skin, Neurological.

For subjects expected to require more than 4 weeks of corticosteroids or other immunosuppressants to manage an AE, consider recommendations provided in the algorithms. The guidance provided in these algorithms should not replace the site investigator's medical judgment but should complement it.

6.7 Treatment of Nivolumab Related Infusion Reactions

Since nivolumab contains only human immunoglobulin protein sequences, it is unlikely to be immunogenic and induce infusion or hypersensitivity reactions. However, if such a reaction were to occur, it might manifest with fever, chills, rigors, headache, rash, pruritis, arthralgias, hypo- or hypertension, bronchospasm, or other symptoms of allergic-like reactions.

All Grade 3 or 4 infusion reactions should be reported as an SAE if criteria are met. Infusion reactions should be graded according to NCI CTCAE v4.03 guidelines. Treatment recommendations are provided below and may be modified based on local treatment standards and guidelines as appropriate:

For Grade 1 symptoms: (Mild reaction; infusion interruption not indicated; intervention not indicated)

- Remain at bedside and monitor subject until recovery from symptoms. The following prophylactic premedications are recommended for future infusions: diphenhydramine 50 mg (or equivalent) and/or paracetamol 325 to 1000 mg (acetaminophen) at least 30 minutes before additional nivolumab administrations.

For Grade 2 symptoms: (Moderate reaction requires therapy or infusion interruption but responds promptly to symptomatic treatment [e.g., antihistamines, non-steroidal anti-inflammatory drugs, narcotics, corticosteroids, bronchodilators, IV fluids]; prophylactic medications indicated for 24 hours).

- Stop the nivolumab infusion, begin an IV infusion of normal saline, and treat the subject with diphenhydramine 50 mg IV (or equivalent) and/or paracetamol 325 to 1000 mg (acetaminophen); remain at bedside and monitor subject until resolution of symptoms. Corticosteroid or bronchodilator therapy may also be administered as appropriate. If the infusion is interrupted, then restart the infusion at 50% of the original infusion rate when symptoms resolve; if no further complications ensue after 30 minutes, the rate may be increased to 100% of the original infusion rate. Monitor subject closely. If symptoms recur, then no further nivolumab will be administered at that visit. Administer diphenhydramine 50 mg IV, and remain at bedside and monitor the subject until resolution of symptoms. The amount of study drug infused must be recorded on the electronic case report form (eCRF). The following prophylactic premedications are recommended for future infusions: diphenhydramine 50 mg (or equivalent) and/or paracetamol 325 to 1000 mg (acetaminophen) should be administered at least 30 minutes before additional nivolumab administrations. If necessary, corticosteroids (recommended dose: up to 25 mg of IV hydrocortisone or equivalent) may be used.

For Grade 3 or Grade 4 symptoms: (Severe reaction, Grade 3: prolonged [i.e., not rapidly responsive to symptomatic medication and/or brief interruption of infusion]; recurrence of symptoms following initial improvement; hospitalization indicated for other clinical sequelae [e.g., renal impairment, pulmonary infiltrates]). Grade 4: (life threatening; pressor or ventilatory support indicated).

- Immediately discontinue infusion of nivolumab. Begin an IV infusion of normal saline, and treat the subject as follows. Recommend bronchodilators, epinephrine 0.2 to 1 mg of a 1:1,000 solution for subcutaneous administration or 0.1 to 0.25 mg of a 1:10,000

solution injected slowly for IV administration, and/or diphenhydramine 50 mg IV with methylprednisolone 100 mg IV (or equivalent), as needed. Subject should be monitored until the investigator is comfortable that the symptoms will not recur. Nivolumab will be permanently discontinued. Investigators should follow their institutional guidelines for the treatment of anaphylaxis. Remain at bedside and monitor subject until recovery from symptoms.

In the case of late-occurring hypersensitivity symptoms (e.g., appearance of a localized or generalized pruritis within 1 week after treatment), symptomatic treatment may be given (e.g., oral antihistamine, or corticosteroids).

6.8 Discontinuation Criteria

Treatment with nivolumab should be permanently discontinued for the following (while all study therapy should be discontinued for the majority of these scenarios, there are rare situations in which it could be acceptable to continue treatment with gemcitabine plus cisplatin despite discontinuing nivolumab. Continuing treatment with gemcitabine plus cisplatin should be discussed and approved by the sponsor-investigator):

- Any Grade 2 drug-related uveitis or eye pain or blurred vision that does not respond to topical therapy and does not improve to Grade 1 severity within the re-treatment period OR requires systemic treatment
- Any Grade 3 non-skin, drug-related adverse event lasting > 7 days, with the following exceptions for drug-related laboratory abnormalities, uveitis, pneumonitis, bronchospasm, hypersensitivity reactions, and infusion reactions, and endocrinopathies:
    - Grade 3 drug-related uveitis, pneumonitis, bronchospasm, hypersensitivity reaction, or infusion reaction of any duration requires discontinuation
    - Grade 3 drug-related endocrinopathies adequately controlled with only physiologic hormone replacement do not require discontinuation
    - Grade 3 drug-related laboratory abnormalities do not require treatment discontinuation except those noted below
- Grade 3 drug-related thrombocytopenia > 7 days or associated with bleeding requires discontinuation
- Any drug-related liver function test (LFT) abnormality that meets the following criteria require discontinuation:
    - AST or ALT > 5 x ULN
    - Total bilirubin > 3 x ULN
    - Concurrent AST or ALT > 3 x ULN and total bilirubin > 2 x ULN
- Any Grade 4 drug-related adverse event or laboratory abnormality, except for the following events which do not require discontinuation:
    - Isolated Grade 4 amylase or lipase abnormalities that are not associated with symptoms or clinical manifestations of pancreatitis and decrease to < Grade 4 within 1 week of onset.
    - Isolated Grade 4 electrolyte imbalances/abnormalities that are not associated with clinical sequelae and are corrected with supplementation/appropriate management within 72 hours of their onset
    - Grade 4 lymphopenia or leukopenia

  - Grade 4 drug-related endocrinopathy adverse events, such as adrenal insufficiency, ACTH deficiency, hyper- or hypothyroidism, or glucose intolerance, which resolve or are adequately controlled with physiologic hormone replacement (corticosteroids, thyroid hormones) or glucose-controlling agents, respectively, may not require discontinuation after discussion with and approval from the sponsor-investigator.
- Any dosing interruption lasting > 6 weeks with the following exceptions (these potential exceptions must be reviewed and approved by the sponsor-investigator):
  - Dosing delays or interruptions to allow for prolonged steroid tapers to manage drug-related adverse events are allowed. Prior to re-initiating treatment in a subject with a dosing interruption lasting > 6 weeks, the Investigator must be consulted. Tumor assessments should continue as per protocol even if dosing is interrupted or delayed
  - Dosing interruptions or delays lasting > 6 weeks that occur for non-drug-related reasons may be allowed if approved by the sponsor-investigator. Prior to re-initiating treatment in a subject with a dosing interruption lasting > 6 weeks, the sponsor-investigator must be consulted. Tumor assessments should continue as per protocol even if dosing is interrupted
- Any adverse event, laboratory abnormality, or intercurrent illness which, in the judgment of the site investigator, presents a substantial clinical risk to the subject with continued nivolumab dosing

6.9 Protocol Therapy Discontinuation

In addition to discontinuation from therapy related to toxicities, a subject will also be discontinued from protocol therapy and followed per protocol under the following circumstances outlined below. The reason for discontinuation of protocol therapy will be documented on the electronic case report form (eCRF)

- Documented disease progression
- Site investigator determines a change of therapy would be in the best interest of the subject
- Subject requests to discontinue protocol therapy, whether due to unacceptable toxicity or for other reasons
  - In a subject decides to prematurely discontinue protocol therapy (“refuses treatment”), the subject should be asked if he or she may still be contacted for further scheduled study assessments. The outcome of that discussion should be documented in both the medical records and in the eCRF.
- Female subject becomes pregnant
- Protocol therapy interruptions as defined above

6.10 Protocol Discontinuation

If a subject decides to discontinue from the protocol (and not just from protocol therapy) all efforts should be made to complete and report study assessments as thoroughly as possible. A complete final evaluation at the time of the subject’s protocol withdrawal should be made with an explanation of why the subject is withdrawing from the protocol. If the reason for removal of a subject from the study is an adverse event, it will be recorded on the eCRF.

SCHEMA FOR CALENDARS

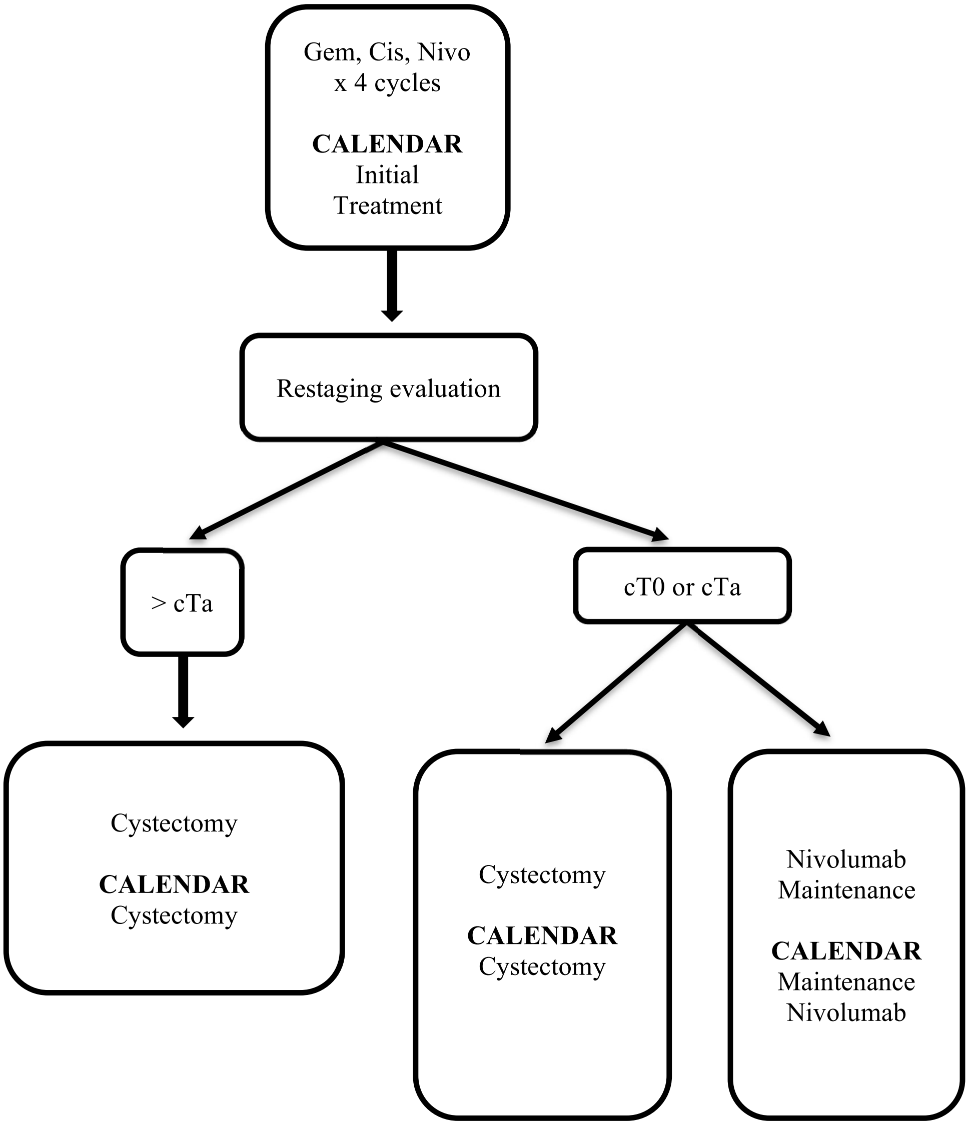

7. STUDY CALENDAR & EVALUATIONS

Initial Treatment Calendar

Study Evaluation<br>Cycle = 21 days (total of 4 cycles)	Screen	Treatment Cycles 1-4		Restaging (within 4 weeks of GC+N)[11]	Post Restaging Visit[12]	**Subjects who do not proceed with maintenance nivo or cystectomy**[13]
	-28 days	Day 1	Day 8			
		± 2 days				
**REQUIRED ASSESSMENTS**						**See Footnote 13 for complete details**<br><br>A **D100 visit** will occur to assess adverse events. (± 7 days)<br><br>After the D100 visit, subjects enter **long term follow up** for disease progression (local and distant) per **Table 8** (± 14 days).<br><br>Once disease progression is documented, subjects enter **survival follow up** for 2 years (± 14 days).
Informed Consent[15]	X[15]				X[15]	
Medical History[1]	X					
Physical Exam	X	X				
Vital signs and ECOG Performance Status[2]	X	X				
AEs & concomitant medications	X	X		X[14]		
**LABORATORY ASSESSMENTS**						
Complete Blood Cell Count with diff (CBC)	X	X[5]	X			
Comprehensive Metabolic Profile (CMP)[3]	X	X[5]	X			
PT/INR and aPTT	X					
Thyroid Function (TSH, T4, free T3)[4]	X	C3D1				
Pregnancy test (serum or urine) (WOCBP)[5]	X[5]	X[5]				
**DISEASE ASSESSMENT**						
CT of chest[6]	X			X[6]		
MRI of abdomen and pelvis (MRI preferred; CT may be performed if MRI contraindicated)[6]	X[6]			X[6]		
Rigid Cystoscopy + biopsies/TURBT[8]				X[8]		
Urine cytology[6]				X[6]		
**TREATMENT**						
Cisplatin		X				
Gemcitabine		X	X			
Nivolumab		X				
**SPECIMEN COLLECTION**						
Archival Tumor Tissue[7]	X					
Tumor and/or Normal tissue[8]				X[8,14]		
Blood Samples[9]		X[9]		X[9,14]		
Urine Sample[10]				X[10,14]		
**BANKING SAMPLES**						
Blood Sample[11]		X[11]				
**FOLLOW UP**						
Survival						

Key to Footnotes

[1.] Medical history will include (1) diagnosis and staging information, (2) prior anti-cancer treatment, (3) smoking questionnaire (4) trial awareness question and (5) genetic sequencing information.

[2.] Vital signs to include blood pressure, weight, and ECOG performance status. Height should be obtained at screening only.

[3] If screening (baseline) CBC and CMP were performed within 7 days of Day 1 of treatment, these do not need to be repeated. CMP to include sodium, potassium, chloride, creatinine, blood urea nitrogen; liver function tests (LFTs) to include AST, ALT, total bilirubin, alkaline phosphatase. CMP at screening to include magnesium, phosphate, uric acid, lactate dehydrogenase

[4] Thyroid function testing will be done at screening and Cycle 3. TSH will be performed in addition to T3 and T4 (free versus total per physician's discretion).

[5] For women of childbearing potential (WOCBP): urine or serum βhCG, within 7 days prior to Day 1.

[6] Imaging studies need not be performed at baseline if completed within 30 days of registration.Within 4 weeks of completion of 4 cycles of gemcitabine, cisplatin, plus nivolumab, subjects will undergo urine cytology and a restaging CT chest and MRI of the abdomen and pelvis. The standard of care preferred imaging sequences for the post-chemotherapy MRI abdomen and pelvis are outlined in Table 7. For patients with contraindications, to MRI, a CT of the abdomen and pelvis may be performed. CT/MRI scans will be submitted for central analysis and images will be de-identified prior to sending to a central location. See Correlative Laboratory Manual (CLM) for additional details.

[7] Archival tissue must be identified at screening and shipped by C2D1. Subjects without available archival tissue must be discussed with the sponsor-investigator. Fixed paraffin-embedded blocks/slides will be obtained in all patients for next generation genomic sequencing as well as for additional research. See Correlative Laboratory Manual (CLM) for additional details.

[8] Fixed paraffin-embedded blocks/slides will be requested from any restaging or progression cystoscopy/TURBTs/urothelial biopsies. Fresh tissue may be obtained at select sites. Snap frozen sections will be obtained from the cystectomy specimen. These will include specimens from specimens without evidence of cancer ("complete response" specimens). Samples should be shipped by post-restaging follow-up visit (for TURBT and/or biopsy specimens). A sample of tissue is required if available to be used for correlative analysis. See CLM for additional details.

[9] Serial blood samples will be collected to support biomarker research. Blood will be collected pre- dose C1D1, C3D1, after completion of Cycle 4 (i.e., at time of restaging evaluation or on follow-up to review restaging results) and at progression. See CLM and for additional details.

[10] Urine will be collected after completion of 4 cycles of gemcitabine, cisplatin, plus nivolumab at the time of restaging or at post-restaging/D30 safety visit as detailed in the CLM.

[11] A blood sample will be collected prior to treatment C1D1 and banked for future unspecified cancer related research. See CLM for additional information.

[12] The post restaging visit will be done within about 2 weeks of the restaging evaluation. Procedures completed during the restaging or post-restaging assessment will meet the requirements of the D30 safety visit for those subjects not proceeding to nivolumab maintenance. During this visit the following will be done (1) review restaging studies, (2) confirm collection and submission of tissue and blood biospecimens and (3) determine plans for *cystectomy* versus *maintenance nivolumab* versus *other treatment*. Subjects who have achieved a cT0 or cTa disease with gem + cis + nivolumab may opt to proceed to the single agent nivolumab phase of the study and be followed per the nivolumab maintenance calendar. Subjects who have achieved a cT0 or cTa disease with gem + cis + nivolumab who opt to proceed to cystectomy will be followed per the cystectomy calendar. Subjects who have > cTa disease with gem + cis + nivolumab will proceed with cystectomy as per standard care and be followed per the cystectomy calendar. Patients who do not proceed directly to other treatment will be followed per the calendar above.

[13] A D100 visit (± 7 days) to assess adverse events will occur for subjects that do not proceed with maintenance nivolumab or any definitive local therapy for any reason. This may be accomplished via clinic visit, phone call, or other avenues as appropriate. After the D100 visit, these subjects will be followed for disease progression (local and distant) per Table 8. Follow up information may be obtained via clinic visit, phone call, or other avenues as appropriate. Once disease progression is documented, subjects will enter a survival follow up period every 6 months for 2 years from the time of documented progression. Follow up may be accomplished via clinic visit, phone call, or other avenues as appropriate.

[14] Correlative samples and AE/conmeds collection done after completion of Cycle 4 should be done at one visit if possible (i.e., EITHER at time of restaging evaluation OR at post-restaging/D30 safety visit). If samples or AE/conmeds were missed at the time of the restaging evaluation, they may be collected at the post-restaging/D30 safety visit. This will not be considered a deviation.

[15] The main consent should be obtained prior to any study related procedures being performed. An additional consent (Addendum ICF) will be obtained at the post staging visit for subjects who have achieved a cT0 or cTa disease with gem + cis + nivolumab and opt to proceed to the single agent nivolumab phase of the study.

Cystectomy Calendar

	Treatment	Follow Up Visit #1 D100 visit[4]	Follow Up Visit #2[5]	Long Term Follow Up[6]	Disease progression ± 14 days	Survival Follow Up[7]
	Cystectomy	30-60 days post-cystectomy	~3 months post Visit #1	± 14 days		± 14 days
**REQUIRED ASSESSMENTS**				**See Table 8**		
Physical Exam		X				
AEs & concomitant medications		X				
**LABORATORY ASSESSMENTS**						
Complete Blood Cell Count with diff (CBC)		X				
Comprehensive Metabolic Profile (CMP)		X				
**DISEASE ASSESSMENT**						
CT of chest[1]			X[1]			
CT or MRI of abdomen and pelvis[1]			X[1]			
**SPECIMEN COLLECTION**						
Tumor and/or Normal tissue[2]	X[2]	X[2]	X[2]		X[2]	
Blood Samples[3]		X[3]	X[3]		X[3]	
**FOLLOW UP**						
Survival						X[7]

[1] The standard of care preferred imaging sequences for the post-chemotherapy MRI of the abdomen and pelvis are outlined in Table 7 and the CLM. For patients with contraindications to MRI, a CT of the abdomen and pelvis may be performed. CT/MRI images scans will be submitted for central analysis and images will be de-identified prior to sending to a central location. See CLM for additional details.

[2] Tissue from the cystectomy specimen is required and will be shipped by the post-cystectomy visit. Fixed paraffin-embedded blocks/slides will be requested from any restaging or progression cystoscopy/TURBTs/urothelial biopsies. See CLM for additional details.

[3] Blood samples will be collected to support biomarker research at Follow Up Visit #1, Follow Up Visit #2 and at progression. See CLM for additional details.

[4]. Follow Up Visit #1 will occur 30-60 days after cystectomy. This visit will be considered the D100 safety visit.

[5] Follow Up Visit #2 will occur about 3 months after Follow Up Visit #1.

[6] Subjects will be followed for disease progression (local and distant) during Long Term Follow Up per Table 8. Follow up information may be obtained via clinic visit, phone call, or other avenues as appropriate.

[7] Once disease progression is documented, subjects will enter a survival follow up period every 6 months for 2 years from the time of documented progression. Follow up may be accomplished via clinic visit, phone call, or other avenues as appropriate.

Maintenance Nivolumab Calendar (only in subjects who have achieved a cT0 or cTa disease with gem + cis + nivolumab (see section 9) and opt for surveillance; treatment should be initiated ~2-6 weeks after completing gem + cis + nivolumab)

Study Evaluation<br>Cycle = 14 days<br>Single agent nivolumab phase = Cycles 5-12<br>(total of 8 cycles)	Cycles 5-12	Restaging	Safety follow up visits[7]	Long-term Follow up[8]	Progression	Survival Follow Up[9]
	Day 1<br>± 2 days	After 4 months<br>(after Cycle 12)[5]	30/100 days post last dose<br>± 7 days	± 14 days	± 14 days	± 14 days
**REQUIRED ASSESSMENTS**						
Informed Consent	X					
Physical Exam	X		D30			
Vital signs and ECOG Performance Status[1]	X		D30			
AEs & concomitant medications	X		D30/100			
**LABORATORY ASSESSMENTS**						
Complete Blood Cell Count with diff (CBC)	X		D30			
Comprehensive Metabolic Profile (CMP)[2]	X		D30			
Thyroid Function (TSH, T4, free T3)[3]	X					
**DISEASE ASSESSMENT**						
CT of chest[4]		X		**Per Table 8**		
CT or MRI of abdomen and pelvis[4]		X				
Cystoscopy +/- biopsies[4]		X				
Urine cytology[4]		X				
**TREATMENT**						
Nivolumab	X					
**SPECIMEN COLLECTION**						
Tumor and/or Normal tissue[5]		X	D30[5]		X[5]	
Blood Samples[6]	X[6]		D30[6]		X[6]	
**FOLLOW-UP**						
Survival Status, Subsequent Therapy						X[9]

CMP to include sodium, potassium, chloride, creatinine, blood urea nitrogen; liver function tests (LFTs) to include AST, ALT, total bilirubin, alkaline phosphatase
CBC with differential and platelet count to include Hgb, platelet count and neutrophil count.

Key to Footnotes

[1] Consent for treatment with nivolumab maintenance will be obtained prior to C1D1.

[2] Vital signs to include blood pressure, weight, and ECOG performance status.

[3] Thyroid function testing will be done prior to C5D1 then every other cycle during treatment (C7, C9, etc.). TSH will be performed in addition to T3 and T4 (free versus total per physician's discretion).

[4] After 4 months of treatment (approximately after cycle 12) of single agent maintenance nivolumab, subjects will undergo a restaging CT chest, MRI or CT of the abdomen and pelvis, and urine cytology. The standard of care preferred imaging sequences for the post-chemotherapy MRI abdomen and pelvis are outlined in Table 7. For patients with contraindications, to MRI, a CT of the abdomen and pelvis may be performed. A restaging cystoscopy +/- biopsies will be performed. Subjects with cT0 or cTa disease will follow-up as described in Table 8. For definition of cT0 or Ta disease, see Section 9. Patients with cT0 or cTa disease, but with positive cytology, will undergo evaluation as per their treating physician. Subjects with cTis disease may receive intravesical therapy as per their treating urologist. Subjects with ≥ cT1 disease will proceed with definitive local treatment as per discussions with their treating physician. . Such patients should subsequently be followed up as per the cystectomy calendar. CT/MRI images will be submitted for central analysis and images will be de-identified prior to sending. See CLM for additional details.

[5] Fixed paraffin-embedded blocks/slides will be requested from any restaging or progression cystoscopy/TURBTs/urothelial biopsies. These samples may also be obtained at progression. These samples may also be obtained at the safety visit after disease progression. See CLM for additional details.

[6] Serial blood samples will be collected to support biomarker research; blood will be collected pre-dose Cycle 8, Cycle 12, and at the time of progression. These samples may also be obtained at the safety visit after disease progression. See CLM for additional details.

[7] The initial safety follow-up visit should only occur when subjects permanently stop study treatment for whatever reasons (toxicity, progression, or at discretion of site investigator) and should be performed 30 days (±7 days) after the last dose of treatment. Subjects who have an ongoing ≥ grade 2 or serious AE (SAE) at this visit will continue to be followed until the AE resolves to ≤ Grade 1 or baseline, deemed clinically insignificant, and/or until a new anti-cancer treatment starts, whichever is earlier. The Day 100 safety follow up visit may be done via phone call or other avenues as appropriate.

[8] For subjects who complete single-agent maintenance nivolumab with no evidence of ≥ cTis, or who discontinue treatment early for any reason with no evidence of ≥ cTis, follow-up should proceed as per Table 8. For subjects with > cTis disease proceeding with cystectomy (or subjects with ≤ cTis disease who *opt to* proceed to cystectomy), subsequent follow-up should be according to Table 8. For subjects who develop cTis, treatment should proceed as per the treating physician.

[9] Once disease progression is documented, subjects will enter a survival follow up period every 6 months for 2 years from the time of documented progression. Follow up may be accomplished via clinic visit, phone call, or other avenues as appropriate.

Table 7. Preferred standard MRI sequences (see CLM for details)

COR HASTE T2 ABD_PEL
AX HASTE T2 ABD_PEL
SAG T2 PEL
AX T2 PEL
COR T2 PEL
AX DWI ABD*
AX DWI PEL*
ABD_PEL_T1 in -and-out-of-phase
PRE_UROGRAM
AX_ABD_PEL_VIBE_PRE
Abd_Pel_AX_VIBE
POST_Urogram

Table 8. Long term follow-up

Procedure	Frequency
Urine cytology (for patients not undergoing cystectomy)	~q3 months; Year 1 and 2<br>~q6 months; Year 3 and 4<br>~Annually; Year 5<br>Subsequently as per treating physician
Cystoscopy (for patients not undergoing cystectomy)	~q3 months Year 1 and 2<br>~q6 months Year 3 and 4<br>~Annually Year 5<br>Subsequently as per treating physician
MRI or CT abdomen/pelvis and CT chest (for all subjects)	~q3 months Year 1 and 1.5<br>~q6 months Year 2 and 2.5<br>~Annually Year 3

*Once disease progression is documented, subjects will enter a survival follow up period every 6 months for 2 years from the time of documented progression. Follow up may be accomplished via clinic visit, phone call, or other avenues as appropriate.

8. BIOSPECIMEN STUDIES AND PROCEDURES

Tumor tissue, peripheral blood, and possibly normal urothelium will be used for biospecimen-based research in this study. Full details of specimen collection and processing can be found in the CLM. Correlative studies will include genomic sequencing of tumor tissue and/or peripheral blood and immune monitoring studies including T cell receptor sequencing and flow cytometry and/or mass cytometry on tissue and/or peripheral blood, and antigen-specific T cell assays. Plasma will also be collected for ctDNA analyses and gene expression profiling. HLA-typing may also be performed to facilitate neoantigen predictions or mechanisns of immunotherapy resistance. Urine will also be collective for correlative analyses including but not limited to circulating DNA and/or cellular subsets.

8.1 Tissue Collection

8.1.1 Archival Tissue

Tumor tissue obtained during the initial TURBT (i.e., prior to study enrollment) will be identified at screening and shipped by C2D1. Subjects without available archival tissue must be discussed with the sponsor-investigator. Fixed paraffin-embedded blocks/slides will be requested from any restaging cystoscopy/TURBTs/urothelial biopsies.

8.1.2 Tumor and normal tissue collection

Normal and tumor tissue from standard of care procedures (i.e., tumor and normal tissue obtained during restaging cystoscopies with TURBT or random bladder biopsies) or from other clinical evaluations (e.g., confirmation of disease progression, management of complication of disease progression, etc.) will be collected during this study. Specimens obtained in these settings may be accessed by the research team to facilitate an understanding of the pharmacodynamic effects of treatment at the level of the tumor and microenvironment including mechanisms of treatment resistance. Cystectomy specimens will be collected and processed for FFPE and snap freezing. Samples will be shipped by post-restaging follow-up visit (for TURBT and/or biopsy specimens) and by the post-cystectomy visit for cystectomy specimens. Fresh tissue may also be collected from the procedures outlined above.

8.1.3 Tissue Analysis/Genomic Sequencing

Archival baseline TURBT tissue will be first utilized for mutation analyses by next generation sequencing at Caris Life Sciences (http://www.carislifesciences.com) using the Caris Molecular Intelligence assay. The assay used in this study is the same assay that is commercially available from Caris Life Sciences and performed in the Caris CLIA, CAP, ISO 18159 and NY State accredited/certified laboratory. Therefore, results will be returned to treating physicians given the potential for the information to inform later lines of treatment should subjects experience disease progression on this protocol. However, the results of this testing will be returned after a decision has been made in subjects achieving a complete clinical response regarding surveillance versus cystectomy and will not be used to assign treatment in this study. Direct sequence analysis will be performed on genomic DNA isolated from a formalin-fixed paraffin-embedded tumor sample using the Illumina NextSeq platform. An Agilent custom-designed SureSelect XT assay will be used to enrich 592 whole-gene targets. Copy number variation will also be determined for a subset of the targets. All variants reported by this assay are detected with >99% confidence based

on the frequency of the mutation present and the amplicon coverage. This assay requires samples to have at least 20% tumor nuclei, following microdissection.

Upon satisfying sample requirements for next generation sequencing, additional nucleic acids extracted from tissue will be stored for future genetic analysis. All remaining tissue will be stored for future immunohistochemistry analyses.

Additional tissue based analyses from baseline, on-treatment, and post-treatment tissue collected as outlined in 8.1.1 and 8.1.2 may include RNA (including the potential for single-cell RNA sequencing)-based analyses, DNA-based analyses (targeted or whole exome sequencing), T-cell receptor sequencing, flow cytometry or mass cytometry, and protein based analyses such as multiplex immunohistochemistry or tissue imaging technologies (e.g., MIBI)

8.2 Whole Blood Samples

For blood samples, the volume of blood and type of tube to be used for each collection is specified in the CLM. Collection of whole blood samples is mandatory for participation in this study.

8.2.1 Whole Blood for T-Cell Analysis

The quantity and composition of immune cells in the peripheral blood will be analyzed by flow cytometry and/or mass cytometry. Antigen specific T cell responses may also be assessed.

8.2.2 Plasma for ctDNA and Gene Expression Profiling

Plasma will be collected for ctDNA. ctDNA will be isolated and analyzed for quantitative expression using sequence specific primers and/or for exome sequencing. RNA may also be isolated from plasma for gene expression profiling.

8.2.3 Whole Blood for Banking

Whole blood will be collected pre-dose C1D1 for banking for future unspecified cancer related research.

8.3 Urine Samples

Urine will be collected after completion of 4 cycles of gemcitabine, cisplatin, plus nivolumab at the time of restaging as detailed in the CLM. Urine will be used to assess for potential biomarkers of benefit from treatment including but not limited to cell free DNA, circulating tumor DNA, and immune cell subsets.

8.4 Storage of Biospecimens

Excess biospecimens not completely utilized in these experiments will be stored indefinitely at HCRN for future use in research focused on GU malignant diseases that are yet to be determined. All specimens collected will maintain the assigned unique study number of the corresponding patient. Coded samples may be shared with other research institutions. We believe that allowing for storage and usage of the remaining samples for future research is ethically justified and a preferred option to discarding these materials given the potential impact on improving clinical outcomes for subjects with bladder cancer. Subjects will be given the option to store excess specimens during the informed consent process.

8.5 Confidentiality of Biospecimens

Samples that are collected will be identified by a subject's study number assigned at the time of registration to the trial. Any material issued to collaborating researchers will be anonymized and only identified by the subject's study number.

9. CRITERIA FOR DISEASE EVALUATION

9.1 Clinical Complete Response

A clinical complete response, or cT0/cTa disease, will be defined as follows:

Modality	Result
Rigid cystoscopy	No evidence of disease or papillary lesions that are resected and confirmed as Ta tumors
Bladder biopsies (if applicable)	< or = to Ta
Urine Cytology	Negative (Subjects with positive urine cytology but otherwise meeting all criteria for a clinical complete response may continue on single-agent nivolumab or surveillance as per protocol but should undergo appropriate workup as per their treating physicians).
Cross sectional imaging	No definitive evidence of local disease as per the treating physician. Given that residual bladder wall thickening, or even the appearance of a mass, can be observed in patients with a complete pathologic response, the assessment should be made by the treating investigator in consultation with the local radiologist and sponsor investigator and in the context of the bladder biopsy results (e.g., patients with residual bladder wall thickening and no evidence of malignancy on biopsies may be considered to have a complete clinical response).<br><br>No evidence of distant metastatic disease. Equivocal lesions should be biopsied for confirmation if possible.

9.9.3 Recurrence-Free Survival

A measurement from the date of initiation of treatment until the development of local or distant recurrence or death occurs. Local recurrence will be defined as invasive recurrences in the intact bladder. Distant recurrence will be defined as per RECIST 1.1. Subjects who have not progressed will be right-censored at the date of the last disease evaluation.

9.9.4 Metastasis-Free Survival

Metastasis-free survival is defined as the time from initiation of treatment to the development of metastatic disease. Confirmation of metastatic recurrence with a biopsy is recommended in all situations.

9.9.5 Overall Survival

Overall survival is defined as the date of initiation of treatment to date of death from any cause.

10. DRUG INFORMATION

10.1 Nivolumab

Nivolumab is an anti-PD1 antibody. Nivolumab is a soluble protein consisting of 4 polypeptide chains, which include 2 identical heavy chains and 2 identical light chains.

Other Names: Nivolumab, BMS-936558, MDX1106, anti-PD-1;**Molecular Wt.**:146,221 daltons (143,619.17 daltons, protein portion); **Appearance**: Clear to opalescent, colorless to pale yellow liquid, few particulates may be present; **Solution pH**: 5.5 to 6.5

10.1.1 Supplier/How Supplied

Nivolumab Injection, 100 mg/10 mL (10 mg/mL) – Investigational Product will be provided.

BMS will supply nivolumab at no charge to subjects participating in this clinical trial.

The site investigator shall take responsibility for and shall take all steps to maintain appropriate records and ensure appropriate supply, storage, handling, distribution, and usage of investigational product in accordance with the protocol and any applicable laws and regulations.

10.1.2 Preparation

Nivolumab Injection, 100 mg/10 mL (10 mg/mL)

Nivolumab injection is to be administered as an IV infusion through a 0.2-micron to 1.2-micron pore size, low-protein binding in-line filter at the protocol-specified doses and infusion times. It is not to be administered as an IV push or bolus injection. Nivolumab injection can be infused undiluted (10 mg/mL) or diluted with 0.9% Sodium Chloride Injection, USP or 5% Dextrose Injection, USP to protein concentrations as low as 0.35 mg/mL. When the dose if fixed, nivolumab injection can be infused undiluted or diluted so as not to exceed a total infusion volume of 120 mL. During drug product preparation and handling, vigorous mixing or shaking is to be avoided. Care must be taken to assure sterility of the prepared solution as the product does not contain any antimicrobial preservative or bacteriostatic agent. No incompatibilities between nivolumab and polyvinyl chloride (PVC) and non-PVC/non-DEHP (di(2-ethylhexyl)phthalate) containers/IV components or glass bottles have been observed.

10.1.3 Storage and Stability

Nivolumab Injection, 100 mg/10 mL (10 mg/mL)

Vials of nivolumab injection must be stored at 2° to 8°C (36° to 46°F) and protected from light and freezing.

Undiluted Nivolumab Injection and Diluted Nivolumab Injection in the IV Container.

The administration of nivolumab infusion must be completed within 24 hours of preparation. If not used immediately, the infusion solution may be stored under refrigeration conditions (2°to 8°C, 36°to 46°F) for up to 24 hours, and a maximum of 8 hours of the total 24 hours can be at room temperature (20° to 25°C, 68° to 77°F) and room light. The maximum 8-hour period under room temperature and room light conditions includes the product administration period.
After final drug reconciliation, unused nivolumab vials should be disposed at the site following procedures for the disposal of anticancer drugs.

10.1.4 Handling and Disposal
Preparation should be performed by trained personnel in accordance with good practices rules, especially with respect to asepsis.

A copy of the drug destruction certificate should be maintained for provision to BMS at the end of the study.

10.1.5 Dispensing
Nivolumab must be dispensed only from official study sites and to eligible subjects under the supervision of the site investigator. Nivolumab should be stored in a secure area according to local regulations. It is the responsibility of the site investigator to ensure that study drug is only dispensed to subjects.

10.1.6 Adverse Events
For a detailed list of adverse events please see the current investigator's brochure. The most common side effects of nivolumab are:

- Fatigue
- Skin reactions: including rash, itching, hives, redness, and dry skin. Toxic epidermal necrolysis, a potentially life threatening disease characterized by blistering and peeling of the top layer of skin resembling that of a severe burn, has occurred in one subject who received ipilimumab after nivolumab treatment.
- Diarrhea
- Nausea
- Abdominal pain
- Decreased appetite
- Low red blood cells
- Fever
- Joint pain or stiffness

10.2 Cisplatin

10.2.1 Drug Name
Cisplatin Other: Platinol (NSC-119875); Cis-diamminedichloroplatinum

10.2.2 Classification
Alkylating agent

10.2.3 Action
Cisplatin forms covalent bonds with nucleophilic sites on guanine present in all DNA. As cisplatin is a bifunctional agent, it is able to bind to 2 sites in a DNA strand. This results in the formation of inter- and intra- chain cross-linkings, which interferes with cellular transcription and replication. Regulatory mechanisms detect the abnormal DNA and so activate a chain of responses to try and correct it. This, ultimately, causes cell death (apoptosis).

10.2.4 Availability
Cisplatin is commercially available.

10.2.5 Storage, Reconstitution, and Administration

Cisplatin should be stored, reconstituted, and administered as per institutional guidelines.

10.2.6 Side Effects

For a detailed list of side effects, please see the current prescribing information. Side effects of cisplatin may include:

- Nephrotoxicity
- Ototoxicity
- Hematologic toxicity (low leukocyte or platelet count, decreased hemoglobin
- Nausea, vomiting, diarrhea
- Hair loss
- Electrolyte disturbances
- Peripheral neuropathy
- Vision problems
- Liver toxicity
- Infusion reaction

10.3 Gemcitabine

10.3.1 Drug Name

Gemcitabine
Other: 1" – Deoxy – 2, 2" – difluorocytidine monohydrochloride, Gemcitabine, NSC #613327

10.3.2 Classification

Nucleoside analogue

10.3.3 Action

Gemcitabine is metabolized intracellularly by nucleoside kinases to the active diphosphate (dFdCDP) and triphosphate (dFdCTP) nucleosides. The cytotoxic effect of gemcitabine is attributed to a combination of 2 actions of the diphosphate and the triphosphate nucleosides, which leads to inhibition of DNA synthesis. First, gemcitabine diphosphate inhibits ribonucleotide reductase, which is responsible for catalyzing the reactions that generate the deoxynucleoside triphosphates for DNA synthesis. Inhibition of this enzyme by the diphosphate nucleoside causes a reduction in the concentrations of deoxynucleotides, including dCTP. Second, gemcitabine triphosphate competes with dCTP for incorporation into DNA. The reduction in the intracellular concentration of dCTP (by the action of the diphosphate) enhances the incorporation of gemcitabine triphosphate into DNA (self-potentiation). After the gemcitabine nucleotide is incorporated into DNA, only 1 additional nucleotide is added to the growing DNA strands. After this addition, there is inhibition of further DNA synthesis. DNA polymerase epsilon is unable to remove the gemcitabine nucleotide and repair the growing DNA strands (masked chain termination).

10.3.4 Availability

Gemcitabine is commercially available

10.3.5 Storage, Reconstitution, and Administration

Gemcitabine should be stored, reconstituted, and administered according to institutional guidelines.

10.3.6 Side Effects

For a detailed list of side effects, please see the current prescribing information. Side effects of gemcitabine may include:

- Nephrotoxicity
- Hematologic toxicity (low platelet count, decreased hemoglobin
- Nausea, vomiting, diarrhea
- Hair loss
- Peripheral neuropathy
- Liver toxicity
- Infusion reaction
- Fever
- Infection
- Rash
- Pulmonary toxicity
- Edema
- Flu-like symptoms

11 ADVERSE EVENTS

The descriptions and grading scales found in the NCI CTCAE v4.03 will be utilized for AE assessment. A copy of the CTCAE v4.03 can be downloaded from the CTEP website at http://ctep.cancer.gov.

11.1 Definitions

11.1.1 Adverse Event (AE)

An AE is any untoward medical occurrence whether or not considered related to the study drug that appears to change in intensity during the course of the study. The following are examples of AEs:

- Unintended or unfavorable sign or symptom
- A disease temporally associated with participation in the protocol
- An intercurrent illness or injury that impairs the well-being of the subject

Abnormal laboratory values or diagnostic test results constitute AEs only if they induce clinical signs or symptoms or require treatment or further diagnostic tests Hospitalization for elective surgery or routine clinical procedures that are not the result of an AE (e.g., surgical insertion of central line) should not be recorded as an AE. Disease progression should not be recorded as an AE, unless it is attributable to the study regimen by the site investigator.

11.1.2 Serious Adverse Event (SAE)

A SAE is an adverse event that:

- Results in death. **NOTE:** Death due to disease progression should not be reported as a SAE, unless it is attributable by the site investigator to the study drug(s)
- Is life-threatening (defined as an event in which the subject was at risk of death at the time of the event; it does not refer to an event which hypothetically might have caused death if it were more severe)
- Requires inpatient hospitalization for >24 hours or prolongation of existing hospitalization. **NOTE:** Hospitalization for anticipated or protocol specified procedures such as administration of chemotherapy, central line insertion, metastasis interventional therapy, resection of primary tumor, or elective surgery, will not be considered serious adverse events.
- Results in persistent or significant disability/incapacity
- Is a congenital anomaly or birth defect
- Is an important medical event (defined as a medical event(s) that may not be immediately life-threatening or result in death or hospitalization but, based upon appropriate medical and scientific judgment, may jeopardize the subject or may require intervention (e.g., medical, surgical) to prevent one of the other serious outcomes listed in the definition above). Examples of such events include, but are not limited to, intensive treatment in an emergency room or at home for allergic bronchospasm; blood dyscrasias or convulsions not resulting in hospitalization; or the development of drug dependency or drug abuse.

11.1.3 Unexpected Adverse Event

For this study, an AE is considered unexpected when it varies in nature, intensity or frequency from information provided in the current IB or when it is not included as a potential risk. Unexpected also refers to AEs that are mentioned in the IB as occurring with a class of drugs or are anticipated from the pharmacological properties of the drug, but are not specifically mentioned as occurring with the particular drug under investigation.

11.1.4 Relatedness

AEs will be categorized according to the likelihood that they are related to the study drug(s). Specifically, they will be categorized using the following terms:

**Unrelated**	Adverse Event is ***not related*** to the study drug(s)
**Unlikely**	Adverse Event is ***doubtfully related*** to the study drug(s)
**Possible**	Adverse Event ***may be related*** to the study drug(s)
**Probable**	Adverse Event is ***likely related*** to the study drug(s)
**Definite**	Adverse Event is ***clearly related*** to the study drug(s)

11.2 Reporting

11.2.1 Adverse Events

- AEs will be recorded from time of signed informed consent until 100 days after discontinuation of study drug(s) or until a new anti-cancer treatment starts, whichever occurs first.
- AEs will be recorded regardless of whether or not they are considered related to the study drug(s).
- All AEs will be recorded in the subject's medical record and on the appropriate study specific eCRF form within the EDC system.
- AEs considered related to study drug(s) will be followed until resolution to ≤ Grade 1 or baseline, deemed clinically insignificant, and/or until a new anti-cancer treatment starts, whichever occurs first.
- Transient asymptomatic laboratory abnormalities that do not require treatment will not be collected as adverse events.

11.2.2 Serious Adverse Events (SAEs)

11.2.2.1 Site Requirements for Reporting SAEs to HCRN

- SAEs will be reported from time of signed informed consent until 100 days after discontinuation of study drug(s) or until a new anti-cancer treatment starts, whichever occurs first.
- SAEs will be reported on the SAE Submission Form **within 1 business day** of discovery of the event.
- SAEs include events related and unrelated to the study drug(s).
- All SAEs will be recorded in the subject's medical record and on the appropriate study specific eCRF form within the EDC system.
- All SAEs regardless of relation to study drug will be followed until resolution to ≤ Grade 1 or baseline and/or deemed clinically insignificant and/or until a new anti-cancer treatment starts, whichever occurs first.

The site will submit the completed SAE Submission Form to HCRN **within 1 business day** of discovery of the event**.** The form may be submitted to HCRN electronically to safety@hoosiercancer.org. The site investigator is responsible for informing the IRB and/or other local regulatory bodies as per local requirements.

The original copy of the SAE Submission Form and the email correspondence must be kept within the study file at the study site.

Once the SAE has resolved (see resolution guidelines listed in 11.2.2.1), sites must submit a follow-up SAE Submission Form within a reasonable timeframe to HCRN electronically to safety@hoosiercancer.org.

11.2.2.2 HCRN Requirements for Reporting SAEs to Bristol-Myers Squibb Company

HCRN will report all SAEs to BMS **within 1 business day** of receipt of the SAE Submission Form from a site. Follow-up information will be provided to BMS as it is received from site. HCRN will submit all SAEs to BMS Global Pharmacovigilance (GPV&E) via email to worldwide.safety@bms.com or fax @ 609-818-3804.

The sponsor-investigator will reconcile the clinical database SAE cases (case level only) transmitted to BMS Global Pharmacovigilance (Worldwide.Safety@bms.com). Frequency of reconciliation must be every 3 months and prior to the database lock or final data summary. BMS GPV&E will email, upon request from the Investigator, the GPV&E reconciliation report. Requests for reconciliation should be sent to aepbusinessprocess@bms.com. The data elements listed on the GPV&E reconciliation report will be used for case identification purposes. If the Investigator determines a case was not transmitted to BMS GPV&E, the case should be sent immediately to BMS.

11.3 Sponsor-Investigator Responsibilities

HCRN will send a SAE summary to the sponsor-investigator **within 1 business day** of receipt of SAE Submission Form from a site. The sponsor-investigator will promptly review the SAE summary and assess for expectedness and relatedness.

11.4 HCRN Responsibilities to FDA

HCRN will manage the Investigational New Drug Application (IND) associated with this protocol on behalf of the sponsor-investigator. HCRN will cross-reference this submission to the Bristol-Myers Squibb's parent IND at the time of submission. Additionally, HCRN will submit a copy of these documents to Bristol-Myers Squibb's at the time of submission to FDA.
For protocols conducted under an IND, HCRN will be responsible for all communication with the FDA in accordance with 21CFR312 including but not limited to the 7 and 15 Day Reports, as well as an Annual Progress Report. Additionally, HCRN will submit a copy of these reports to Bristol-Myers Squibb's at the time of submission to FDA.

11.5 IND Safety Reports Unrelated to this Trial

Bristol-Myers Squibb's will provide to HCRN IND safety reports (through the FastTrack Portal) from external studies that involve the study drug(s) per their guidelines. HCRN will forward safety reports to the sponsor-investigator who will review these reports and determine if revisions are needed to the protocol or consent. HCRN will forward these reports to participating sites **within 1 business day** of receiving the sponsor-investigator's review. Based on the sponsor-investigator's review, applicable changes will be made to the protocol and informed consent document (if required). All IND safety reports will also be made available to sites via the EDC system.

Upon receipt from HCRN, site investigators (or designees) are responsible for submitting these safety reports to their respective IRBs, as per their IRB policies.

12 STATISTICAL METHODS

12.1 Study Design

This is a phase 2 trial seeking to define the safety and activity of gemcitabine, cisplatin, plus nivolumab as neoadjuvant therapy in patients with muscle-invasive bladder cancer and to define the role of clinical complete response in predicting benefit in patients opting to avoid cystectomy.

12.2 Endpoints

12.2.1 Definition of Primary Endpoint

Clinical complete response rate will be defined as cT0 or cTa disease after gemcitabine, cisplatin, plus nivolumab. Benefit from treatment will be defined as a pathologic complete response (<pT1) in patients undergoing cystectomy and 2 years metastasis-free in patients proceeding with surveillance

12.2.2 Definition of Secondary Endpoints

- Safety will be determined according to the NCI Common Terminology Criteria for Adverse Events (NCI CTCAE) v4.03
- Bladder-intact overall survival is defined as the time from initiation of treatment until death or cystectomy
- Recurrence-free survival which is defined as the time from initiation of treatment to death or recurrence, depending on which occurs first
- Overall survival is defined as the time from initiation of treatment to death
- Pathologic complete response rate in patients undergoing radical cystectomy is defined as the proportion of patients with <pT1
- Positive predictive value of a panel of genomic alterations in predicting benefit from treatment

12.3 Sample Size and Power Analysis

In our sample size justification and power analysis, we make the following assumptions.

1. Patients without a clinical complete response (i.e, cT0 or cTa disease post-treatment) would not be suitable for surveillance (i.e., patients with cT0 or cTa disease will not have a pathologic complete response). Non clinical complete response implies no benefit.
2. Approximately 40% of patients will have clinical complete response (though few studies have prospectively explored the clinical complete response rate with cisplatin-based chemotherapy, note that the clinical complete response rate with gemcitabine, carboplatin, plus paclitaxel in was 46% in the SWOG 0219 study from carboplatin-based chemotherapy was 46%).
3. Approximately 35% of patients will have a pathologic complete response (or if not undergoing cystectomy will be metastasis-free at 2 years). True benefit rate is around 35%
4. The overall prevalence of having at least one of the genomic alterations pre-specified in our analysis is 19%~37% per the table below.

Biomarker	Functional Prediction*	Prevalence
FANCC, ATM, RB1, and/or ERCC2	Pathogenic + Presumed Pathogenic	19.3%
	Pathogenic + Presumed Pathogenic + Variant of Unknown Significance	32.9%
FANCC, ATM, RB1, ERCC2, and/or high Tumor Mutational Load	Pathogenic + Presumed Pathogenic	26.6%
	Pathogenic + Presumed Pathogenic + Variant of Unknown Significance	37.1%

*Presumed benign and benign mutations excluded

Our primary analysis will focus on the ability of clinical complete response (cT0 + cTa) to predict benefit as defined by <pT1 in patients undergoing cystectomy and 2-years metastasis-free in patients opting for surveillance. However, a secondary analysis will incorporate the association between having at least one of the biomarkers noted above and benefit as based on prior data, the use of the biomarker panel may increase specificity compared with clinical response assessment but decrease sensitivity. Then the assumption 1 basically implies that the negative predictive value (NPV) of clinical complete response alone is 1. Our sample size is then based on the confidence interval width of the positive predictive value (PPV) of clinical complete response alone. From our assumptions 1, 2, and 3, we have the following table (Table 9):

Table 9: Distribution of cT0 and benefit status

	**Clinical complete response**	**No clinical complete response**	*Total*
**Benefit="yes"**	**35%**	**0%**	*35%*
**Benefit="no"**	**5%**	**60%**	*65%*
Total	*40%*	*60%*	*100%*

The PPV from the table is 87.5%. The sample size is generated such that the lower bound of the 95% one-sided confidence interval exceeds 80%. Based on the formula from Steinberg et al[35] available from the "bdpv" package in the r language, a sample size of 68 satisfies this criterion. Assuming a 10% missing data rate (e.g. due to unmeasurable biomarker or unobservable outcome), we propose to recruit 76 patients.

Next we consider the association between the prespecified panel of genomic biomarkers and benefit from treatment in patients achieving a clinical complete response. In particular, we will test whether the presence of alternations in the prespecified panel of genomic biomarkers (which we name as biomarker+) is associated with benefit among patients achieving a clinical complete response. With 68 patients, we assume 28 will achieve a clinical complete response. Now based on preliminary results of the biomarker association (Table 3 in Section 1.4), we assume 90% of the biomarker+ patients will achieve a clinical complete response. Assuming also that at least 90% of the biomarker+ and clinical complete response patients will have benefit="yes", the power levels based on the Fisher's exact test (with type I error of 0.05) range from 0.39 to 0.92 when the overall prevalence of being biomarker+ goes from 19% to 37%. The two extreme cases are listed in the following two tables (Tables 10 and 11).

Table 10: Power = 0.39 for the association between biomarker and pCR among cT0="yes" patients

	**Clinical complete response**		**No clinical complete response**	*Total*
	**Biomarker+**	**Biomarker-**		
**Benefit="yes"**	**5**	**18**	**0**	*23*
**Benefit="no"**	**1**	**4**	**40**	*45*
Total	*6*	*22*	*40*	*68*

Table 11: Power = 0.92 for the association between biomarker and pCR among cT0="yes" patients

	**Clinical complete response**		**No clinical complete response**	*Total*
	**Biomarker+**	**Biomarker-**		
**Benefit="yes"**	**11**	**12**	**0**	*23*
**Benefit="no"**	**0**	**5**	**40**	*45*
Total	*11*	*17*	*40*	*68*

12.4 Data Analysis Plans

12.4.1 Analysis Plans for Primary Objective

All subjects who have received at least one cycle of treatment will be evaluable for the primary endpoint. To determine the ability of clinical complete response (cT0 or cTa) to predict benefit from treatment, sensitivity, specificity, PPV, and NPV will be calculated, together with confidence intervals. Concordance measure such as Cohen's Kappa between clinical complete response and benefit will also be calculated.

12.4.2 Analysis Plans for Secondary Objectives

Any subject who receives at least one dose of treatment on this protocol is evaluable for toxicity. Toxicity rates will be summarized using frequency tables. Time to event outcomes (i.e. local recurrence free survival, overall survival, and bladder-intact overall survival) will be analyzed using the Kaplan-Meier method. Pathologic complete response rate in patients undergoing radical cystectomy will be summarized using frequency tables. PPV (and sensitivity and specificity) of the panel of genomic alterations in predicting benefit from treatment will also be analyzed.

12.4.3 Analysis Plans for Exploratory Objectives

Association of markers of immune among themselves will be conducted using the Pearson correlation. Association of markers of immune with the time-to-event clinical outcomes will be explored using the Cox proportional hazards model. Model assumptions will be checked and if violated, other models will be attempted. Association of markers of immune modulation with clinical complete response, and benefit status where applicable, will be conducted using t-test.

Association between genomic alterations and clinical complete response, and benefit status, will be conducted using Chi-square test. Association between genomic alterations and time-to-event outcomes will be explored using the log-rank test. These analyses will also be performed on circulating tumor DNA, gene expression, and radiographic characteristics.

12.5 Interim Analysis/Criteria for Stopping Study

We will use three stopping rules for this study.

1. The first is for excessive immune-related Grade ≥ 3 adverse events,
2. The second is for high muscle-invasive local recurrence rates, and the
3. Third is for high metastatic recurrence rates.

The first stopping rule is for <u>all subjects</u> whereas the second and third rules are <u>among those subjects achieving clinical complete response and proceeding with surveillance</u>.

The first stopping rule will be employed after 7, 14, and 21 subjects finish at least one cycle of treatment. This safety stopping rule will be performed with respect to immune-related Grade ≥ 3 adverse events. Two-sided 95% exact binomial confidence intervals (CIs) of these Grade ≥ 3 immune-related adverse event rates will be constructed. If their lower bounds exceed 3%, the study drug will be considered unacceptably toxic for this patient population and enrollment will be halted. The 3% is chosen based on the toxicity profile of single agent PD-1 or PD-L1 blockade in patients with urothelial cancer.[24,36] This corresponds to ≥ 2+ (2 or more) out of 7, ≥ 3+ (3 or more) out of 14, and ≥ 3+ out of 21 subjects.

The second and third stopping rule will be employed only among those subjects achieving clinical complete response and proceeding with surveillance, and excessive muscle-invasive bladder cancer recurrence and/or metastatic recurrence would be of concern. We will stop the trial if we are confident that either the muscle-invasive local recurrence rate or the metastatic recurrence rate exceed 30%.[13] Two-sided 95% exact binomial confidence intervals (CIs) of this recurrence rate will be constructed. If their lower bounds exceed 30%, enrollment will be halted. This corresponds to 5 out of 5, ≥ 7+ (7 or more) out of 10, ≥ 9+ out of 15 subjects, and ≥ 11+ out of 20 subjects.

Enrollment will not be held for data maturations/stopping rule assessments.

13 TRIAL MANAGEMENT

13.1 Data and Safety Monitoring Plan (DSMP)

The study will be conducted with guidance from the Icahn School of Medicine/Tisch Cancer Institute’s DSMP

HCRN oversight activities include:

- Review and process all adverse events requiring expedited reporting as defined in the protocol
- Provide trial accrual progress, safety information and data summary reports to the sponsor-investigator
- Submit data summary reports to the DSMB for review according to DSMB Charter

13.2 Tisch Cancer Institute Cancer Center Data Safety Monitoring Committee

HCRN will provide the following for the DSMC to review:

- Adverse event summary report
- Audit results, if applicable
- Data related to stopping/decision rules described in study design
- Study accrual patterns
- Protocol deviations

The DSMC will review study data at least every 6 months. Documentation of DSMC reviews will be provided to sponsor-investigator and HCRN. Issues of immediate concern by the DSMC will brought to the attention of the sponsor-investigator and other regulatory bodies as appropriate. The sponsor-investigator will work with HCRN to address the DSMC's concerns.

13.3 Data Quality Oversight Activities

Remote validation of EDC system data will be completed on a continual basis throughout the life cycle of the study. Automated edit check listings will be used to generate queries in the EDC system and transmitted to the site to address in a timely fashion. Corrections will be made by the study site personnel. Participating sites may also be subject to quality assurance audits as well as inspection by appropriate regulatory agencies.

13.3.1 Onsite Monitoring

Monitoring visits to the trial sites may be made periodically during the trial to ensure key aspects of the protocol are followed. Selected source documents will be reviewed for verification of agreement with data entered into the EDC system. It is important for the site investigator and their relevant personnel to be available for a sufficient amount of time during the monitoring visits or audit, if applicable. The site investigator and institution guarantee access to source documents by HCRN or its designee.

The trial site may also be subject to quality assurance audit by the sponsor-investigator or its designee, as well as inspection by appropriate regulatory agencies.

13.4 Compliance with Trial Registration and Results Posting Requirements

Under the terms of the Food and Drug Administration Modernization Act (FDAMA) and the Food and Drug Administration Amendments Act (FDAAA), the sponsor-investigator of the trial is solely responsible for determining whether the trial and its results are subject to the requirements for submission to the Clinical Trials Data Bank, http://www.clinicaltrials.gov. All results of primary and secondary objectives must be posted to CT.gov within a year of completion. The sponsor-investigator has delegated responsibility to HCRN for registering the trial and posting the results on clinicaltrials.gov. Information posted will allow subjects to identify potentially appropriate trials for their disease conditions and pursue participation by calling a central contact number for further information on appropriate trial locations and study site contact information.

14. DATA HANDLING AND RECORD KEEPING

14.1 Data Management

HCRN will serve as the Clinical Research Organization for this trial. Data will be collected through a web based clinical research platform, a system compliant with Good Clinical Practices and Federal Rules and Regulations. HCRN personnel will coordinate and manage data for quality control assurance and integrity. All data will be collected and entered into the EDC system by study site personnel from participating institutions.

14.2 Case Report Forms and Submission

Generally, clinical data will be electronically captured in the EDC system and correlative results will be captured in the EDC system or other secure database(s). If procedures on the study calendar are performed for standard of care, at minimum, that data will be captured in the source document. Select standard of care data will also be captured in the EDC system, according to study-specific objectives.

The completed dataset is the sole property of the sponsor-investigator's institution and should not be exported to third parties, except for authorized representatives of appropriate Health/Regulatory Authorities, without permission from the sponsor-investigator and HCRN.

14.3 Record Retention

To enable evaluations and/or audits from Health Authorities/HCRN, the site investigator agrees to keep records, including the identity of all subjects (sufficient information to link records; e.g., hospital records), all original signed informed consent forms, copies of all source documents, and detailed records of drug disposition. All source documents are to remain in the subject's file and retained by the site investigator in compliance with the site contract with HCRN. No records will be destroyed until HCRN confirms destruction is permitted.

14.4 Confidentiality

There is a slight risk of loss of confidentiality of subject information. All records identifying the subjects will be kept confidential and, to the extent permitted by the applicable laws and/or regulations, will not be made publicly available. Information collected will be maintained on secure, password protected electronic systems. Paper files that contain personal information will be kept in locked and secure locations only accessible to the study site personnel.

Subjects will be informed in writing that some organizations including the sponsor-investigator and his/her research associates, HCRN, Bristol-Myers Squibb's, IRB, or government agencies, like the FDA, may inspect their medical records to verify the information collected, and that all personal information made available for inspection will be handled in strictest confidence and in accordance with local data protection laws.

If the results of the study are published, the subject's identity will remain confidential.

15 ETHICS

15.1 Institutional Review Board (IRB) Approval

The final study protocol and the final version of the informed consent form must be approved in writing by an IRB. The site investigator must submit written approval by the IRB to HCRN before he or she can enroll subjects into the study.

The site investigator is responsible for informing the IRB of any amendment to the protocol in accordance with local requirements. In addition, the IRB must approve all advertising used to recruit subjects for the study. The protocol must be re-approved by the IRB, as local regulations require.

Progress reports and notifications of serious and unexpected adverse events will be provided to the IRB according to local regulations and guidelines.

15.2 Ethical Conduct of the Study

The study will be performed in accordance with ethical principles originating from the Declaration of Helsinki. Conduct of the study will be in compliance with ICH Good Clinical Practice, and with all applicable federal (including 21 CFR parts 56 & 50), state, or local laws.

15.3 Informed Consent Process

The site investigator will ensure the subject is given full and adequate oral and written information about the nature, purpose, possible risks and benefits of the study. Subjects must also be notified they are free to discontinue from the study at any time. The subject should be given the opportunity to ask questions and allowed time to consider the information provided.

The subject's signed and dated informed consent must be obtained before conducting any procedure specifically for the study. The site investigator must store the original, signed informed consent form. A copy of the signed informed consent form must be given to the subject.

16 REFERENCES

1. Stimson CJ, Chang SS, Barocas DA, et al. Early and Late Perioperative Outcomes Following Radical Cystectomy: 90-Day Readmissions, Morbidity and Mortality in a Contemporary Series. J Urol 2010;184(4):1296–300.
2. Hounsome LS, Verne J, McGrath JS, Gillatt DA. Trends in Operative Caseload and Mortality Rates after Radical Cystectomy for Bladder Cancer in England for 1998–2010. Eur Urol 2015;67(6):1056–62.
3. Liberman D, Lughezzani G, Sun M, et al. Perioperative Mortality Is Significantly Greater in Septuagenarian and Octogenarian Patients Treated With Radical Cystectomy for Urothelial Carcinoma of the Bladder. Urology 2011;77(3):660–6.
4. Marqueen KE, Waingankar N, Sfakianos J, et al. Identifying high surgical risk in muscle-invasive bladder cancer (MIBC) patients undergoing radical cystectomy (RC). J Clin Oncol 2018;36(6_suppl):460–460.
5. Bochner BH, Kattan MW, Vora KC. Postoperative nomogram predicting risk of recurrence after radical cystectomy for bladder cancer. J Clin Oncol 2006;24(24):3967–72.
6. Gray PJ, Fedewa SA, Shipley WU, et al. Use of Potentially Curative Therapies for Muscle-invasive Bladder Cancer in the United States: Results from the National Cancer Data Base. Eur Urol 2012;
7. Grossman HB, Natale RB, Tangen CM, et al. Neoadjuvant chemotherapy plus cystectomy compared with cystectomy alone for locally advanced bladder cancer. N Engl J Med 2003;349(9):859–66.
8. Griffiths G, Hall R, Sylvester R, Raghavan D, Parmar MK. International phase III trial assessing neoadjuvant cisplatin, methotrexate, and vinblastine chemotherapy for muscle-invasive bladder cancer: long-term results of the BA06 30894 trial. J Clin Oncol 2011;29(16):2171–7.
9. Neoadjuvant chemotherapy in invasive bladder cancer: update of a systematic review and meta-analysis of individual patient data advanced bladder cancer (ABC) meta-analysis collaboration. Eur Urol 2005;48(2):202–6.
10. Prado K, Gollapudi K, King C, Steinberg ML, Chin AI. Bladder preservation in the treatment of muscle-invasive bladder cancer. Bladder 2014;1(1):5.
11. Rosenblatt R, Sherif A, Rintala E, et al. Pathologic downstaging is a surrogate marker for efficacy and increased survival following neoadjuvant chemotherapy and radical cystectomy for muscle-invasive urothelial bladder cancer. Eur Urol 2012;61(6):1229–38.
12. Sternberg CN, Pansadoro V, Calabro F, et al. Neo-adjuvant chemotherapy and bladder preservation in locally advanced transitional cell carcinoma of the bladder. Ann Oncol 1999;10(11):1301–5.
13. Herr HW, Bajorin DF, Scher HI. Neoadjuvant chemotherapy and bladder-sparing surgery for invasive bladder cancer: ten-year outcome. J Clin Oncol 1998;16(4):1298–301.
14. deVere White RW, Lara PN, Goldman B, et al. A sequential treatment approach to myoinvasive urothelial cancer: a phase II Southwest Oncology Group trial (S0219). J Urol 2009;181(6):2476-80-1.
15. Thomas DJ, Roberts JT, Hall RR, Reading J. Radical transurethral resection and chemotherapy in the treatment of muscle-invasive bladder cancer: a long-term follow-up. BJU Int 1999;83(4):432–7.

16. Moran GW, Li G, Robins DJ, Matulay JT, McKiernan JM, Anderson CB. Systematic Review and Meta-Analysis on the Efficacy of Chemotherapy with Transurethral Resection of Bladder Tumors as Definitive Therapy for Muscle Invasive Bladder Cancer. Bl Cancer 2017;3(4):245–58.
17. Galsky MD, Chen GJ, Oh WK, et al. Comparative effectiveness of cisplatin-based and carboplatin-based chemotherapy for treatment of advanced urothelial carcinoma. Ann Oncol 2011;23(2):406–10.
18. Meyer A, Ghandour R, Bergman A, et al. The Natural History of Clinically Complete Responders to Neoadjuvant Chemotherapy for Urothelial Carcinoma of the Bladder. J Urol 2014;192(3):696–701.
19. Solsona E, Climent MA, Iborra I, et al. Bladder Preservation in Selected Patients with Muscle-Invasive Bladder Cancer by Complete Transurethral Resection of the Bladder Plus Systemic Chemotherapy: Long-Term Follow-up of a Phase 2 Nonrandomized Comparative Trial with Radical Cystectomy. Eur Urol 2009;55(4):911–21.
20. Iyer G, Balar AV, Milowsky MI, et al. Correlation of DNA damage response (DDR) gene alterations with response to neoadjuvant (neo) dose-dense gemcitabine and cisplatin (ddGC) in urothelial carcinoma (UC). ASCO Meet Abstr 2016;34(15_suppl):5011.
21. Van Allen EM, Mouw KW, Kim P, et al. Somatic ERCC2 Mutations Correlate with Cisplatin Sensitivity in Muscle-Invasive Urothelial Carcinoma. Cancer Discov 2014;4(10):1140–53.
22. Liu D, Plimack ER, Hoffman-Censits J, et al. Clinical Validation of Chemotherapy Response Biomarker ERCC2 in Muscle-Invasive Urothelial Bladder Carcinoma. JAMA Oncol 2016;
23. Plimack ER, Dunbrack RL, Brennan TA, et al. Defects in DNA Repair Genes Predict Response to Neoadjuvant Cisplatin-based chemotherapy in Muscle-invasive Bladder Cancer. Eur Urol 2015;
24. Rosenberg JE, Hoffman-Censits J, Powles T, et al. Atezolizumab in patients with locally advanced and metastatic urothelial carcinoma who have progressed following treatment with platinum-based chemotherapy: a single-arm, multicentre, phase 2 trial. Lancet (London, England) 2016;
25. Balar AV, Galsky MD, Loriot Y, et al. Atezolizumab (atezo) as first-line (1L) therapy in cisplatin-ineligible locally advanced/metastatic urothelial carcinoma (mUC): Primary analysis of IMvigor210 cohort 1. ASCO Meet Abstr 2016;34(18_suppl):LBA4500.
26. Snyder A, Makarov V, Merghoub T, et al. Genetic Basis for Clinical Response to CTLA-4 Blockade in Melanoma. N Engl J Med 2014;371(23):141119140020009.
27. Rizvi NA, Hellmann MD, Snyder A, et al. Mutational landscape determines sensitivity to PD-1 blockade in non-small cell lung cancer. Science (80-) 2015;
28. Yap KL, Kiyotani K, Tamura K, et al. Whole-exome sequencing of muscle-invasive bladder cancer identifies recurrent mutations of UNC5C and prognostic importance of DNA repair gene mutations on survival. Clin Cancer Res 2014;20(24):6605–17.
29. Le DT, Uram JN, Wang H, et al. PD-1 Blockade in Tumors with Mismatch-Repair Deficiency. N Engl J Med 2015;372(26):2509–20.
30. Teo MY, Seier K, Ostrovnaya I, et al. Alterations in DNA Damage Response and Repair Genes as Potential Marker of Clinical Benefit From PD-1/PD-L1 Blockade in Advanced Urothelial Cancers. J Clin Oncol 2018;JCO2017757740.
31. Sharma P, Callahan MK, Bono P, et al. Nivolumab monotherapy in recurrent metastatic

urothelial carcinoma (CheckMate 032): a multicentre, open-label, two-stage, multi-arm, phase 1/2 trial. Lancet Oncol 2016;17(11):1590–8.

32. Galsky MD, Retz MM, Siefker-Radtke AO, et al. Efficacy and safety of nivolumab monotherapy in patients with metastatic urothelial cancer who have received prior treatment: Results from the phase II CheckMate 275 study. In: ESMO. 2016.
33. Rizvi NA, Hellmann MD, Brahmer JR, et al. Nivolumab in Combination With Platinum-Based Doublet Chemotherapy for First-Line Treatment of Advanced Non-Small-Cell Lung Cancer. J Clin Oncol 2016;34(25):2969–79.
34. Kanda S, Goto K, Shiraishi H, et al. Safety and efficacy of nivolumab and standard chemotherapy drug combination in patients with advanced non-small-cell lung cancer: a four arms phase Ib study. Ann Oncol 2016;mdw416.
35. Steinberg DM, Fine J, Chappell R. Sample size for positive and negative predictive value in diagnostic research using case-control designs. Biostatistics 2008;10(1):94–105.
36. Galsky MD, Retz M, Siefker-Radtke AO, et al. Efficacy and safety of nivolumab monotherapy in patients with metastatic urothelial cancer who have received prior treatment. In: ESMO. 2016.

APPENDIX A: AE MANAGEMENT ALGORITHMS

GI Adverse Event Management Algorithm

Rule out non-inflammatory causes. If non-inflammatory cause is identified, treat accordingly and continue I-O therapy. Opiates/narcotics may mask symptoms of perforation. Infliximab should not be used in cases of perforation or sepsis.

Grade of Diarrhea/ Colitis (NCI CTCAE v4)	Management	Follow-up
**Grade 1**<br>Diarrhea: < 4 stools/day over baseline;<br>Colitis: asymptomatic	• Continue I-O therapy per protocol<br>• Symptomatic treatment	• Close monitoring for worsening symptoms.<br>• Educate patient to report worsening immediately<br>If worsens:<br>• Treat as Grade (G) 2 or 3/4
**Grade 2**<br>Diarrhea: 4-6 stools per day over baseline; IV fluids indicated <24 hours (hrs); not interfering with ADL<br>Colitis: abdominal pain; blood in stool	• Delay I-O therapy per protocol<br>• Symptomatic treatment	If improves to grade 1:<br>• Resume I-O therapy per protocol<br>If persists > 5-7 days or recur:<br>• 0.5-1.0 mg/kg/day methylprednisolone or oral equivalent<br>• When symptoms improve to grade 1, taper steroids over at least 1 month, consider prophylactic antibiotics for opportunistic infections, and resume I-O therapy per protocol.<br>If worsens or persists > 3-5 days with oral steroids:<br>• Treat as grade 3/4
**Grade 3-4**<br>Diarrhea (G3): ≥7 stools per day over baseline; incontinence; IV fluids ≥24 hrs; interfering with activities of daily living (ADL)<br>Colitis (G3): severe abdominal pain, medical intervention indicated, peritoneal signs<br>G4: life-threatening, perforation	• Discontinue I-O therapy per protocol<br>• 1.0 to 2.0 mg/kg/day methylprednisolone IV or IV equivalent<br>• Add prophylactic antibiotics for opportunistic infections<br>• Consider lower endoscopy	If improves:<br>• Continue steroids until grade 1, then taper over at least 1 month<br>If persists > 3-5 days, or recurs after improvement:<br>• Add infliximab 5 mg/kg (if no contraindication). Note: Infliximab should not be used in cases of perforation or sepsis

Patients on IV steroids may be switched to an equivalent dose of oral corticosteroids (e.g. prednisone) at start of tapering or earlier, once sustained clinical improvement is observed. Lower bioavailability of oral corticosteroids should be taken into account when switching to the equivalent dose of oral corticosteroids.

Renal Adverse Event Management Algorithm

Rule out non-inflammatory causes. If non-inflammatory cause, treat accordingly and continue I-O therapy

Grade of Creatinine Elevation (NCI CTCAE v4)	Management	Follow-up
**Grade 1**<br>Creatinine > upper limit of normal (ULN) and > than baseline but ≤ 1.5x baseline	• Continue I-O therapy per protocol<br>• Monitor creatinine weekly	<u>If returns to baseline</u>:<br>•Resume routine creatinine monitoring per protocol<br><u>If worsens:</u><br>•Treat as Grade 2 or 3/4
**Grade 2-3**<br>Creatinine > 1.5x baseline to ≤ 6x ULN	• Delay I-O therapy per protocol<br>• Monitor creatinine every 2-3 days<br>• 0.5 to 1.0 mg/kg/day methylprednisolone IV or oral equivalent<br>• Consider renal biopsy	<u>If returns to Grade 1:</u><br>•Taper steroids over at least 1 month, consider prophylactic antibiotics for opportunistic infections, and resume I-O therapy and routine creatinine monitoring per protocol<br><br><u>If elevations persist > 7 days or worsen:</u><br>•Treat as Grade 4
**Grade 4**<br>Creatinine > 6x ULN	• Discontinue I-O therapy per protocol<br>• Monitor creatinine daily<br>• 1.0-2.0 mg/kg/day methylprednisolone IV or IV equivalent<br>• Consult nephrologist<br>• Consider renal biopsy	<u>If returns to Grade 1</u>: Taper steroids over at least 1 month and add prophylactic antibiotics for opportunistic infections

Patients on IV steroids may be switched to an equivalent dose of oral corticosteroids (e.g. prednisone) at start of tapering or earlier, once sustained clinical improvement is observed. Lower bioavailability of oral corticosteroids should be taken into account when switching to the equivalent dose of oral corticosteroids.

Pulmonary Adverse Event Management Algorithm

Rule out non-inflammatory causes. If non-inflammatory cause, treat accordingly and continue I-O therapy. Evaluate with imaging and pulmonary consultation.

Grade of Pneumonitis (NCI CTCAE v4)	Management	Follow-up
**Grade 1** Radiographic changes only	• Consider delay of I-O therapy<br>• Monitor for symptoms every 2-3 days<br>• Consider Pulmonary and Infectious Disease (ID) consults	• Re-image at least every 3 weeks<br>If worsens:<br>• Treat as Grade 2 or 3-4
**Grade 2** Mild to moderate new symptoms	• Delay I-O therapy per protocol<br>• Pulmonary and ID consults<br>• Monitor symptoms daily, consider hospitalization<br>• 1.0 mg/kg/day methyl-prednisolone IV or oral equivalent<br>• Consider bronchoscopy, lung biopsy	• Re-image every 1-3 days<br>If improves:<br>• When symptoms return to near baseline, taper steroids over at least 1 month and then resume I-O therapy per protocol and consider prophylactic antibiotics<br>If not improving after 2 weeks or worsening:<br>• Treat as Grade 3-4
**Grade 3-4** Severe new symptoms; New/worsening hypoxia; Life-threatening	• Discontinue I-O therapy per protocol<br>• Hospitalize<br>• Pulmonary and ID consults<br>• 2-4 mg/kg/day methylprednisolone IV or IV equivalent<br>• Add prophylactic antibiotics for opportunistic infections<br>• Consider bronchoscopy, lung biopsy	If improves to baseline:<br>• Taper steroids over at least 6 weeks<br>If not improving after 48 hours or worsening:<br>• Add additional immunosuppression (e.g. infliximab, cyclophosphamide, intravenous immunoglobulin (IVIG), or mycophenolate mofetil)

Patients on IV steroids may be switched to an equivalent dose of oral corticosteroids (e.g. prednisone) at start of tapering or earlier, once sustained clinical improvement is observed. Lower bioavailability of oral corticosteroids should be taken into account when switching to the equivalent dose of oral corticosteroids.

Hepatic Adverse Event Management Algorithm

Rule out non-inflammatory causes. If non-inflammatory cause, treat accordingly and continue I-O therapy. Consider imaging for obstruction.

Grade of Liver Test Elevation (NCI CTCAE v4)	Management	Follow-up
**Grade 1**<br>AST or ALT > ULN to 3.0 x ULN and/or Total bilirubin (T. bili) > ULN - 1.5 x ULN	• Continue I-O therapy per protocol	• Continue liver function tests (LFT) monitoring per protocol<br>If worsens:<br>• Treat as Grade 2 or 3-4
**Grade 2**<br>AST or ALT > 3.0 to ≤ 5 x ULN and/or T. bili > 1.5 to ≤ 3 x ULN	• Delay I-O therapy per protocol<br>• Increase frequency of monitoring to every 3 days	If returns to baseline:<br>•Resume routine monitoring, resume I-O therapy per protocol<br><br>If elevations persist > 5-7 days or worsen :<br>• 0.5-1 mg/kg/day methylprednisolone or oral equivalent and when LFT returns to grade 1 or baseline, taper steroids over at least 1 month, consider prophylactic antibiotics for opportunistic infections, and resume I-O therapy per protocol
**Grade 3-4**<br>AST or ALT > 5 x ULN and /or T.bili >3 x ULN	• Discontinue I-O therapy*<br>• Increase frequency of monitoring to every 1-2 days<br>• 1.0 to 2.0 mg/kg/day methylprednisolone IV or IV equivalent**<br>• Add prophylactic antibiotics for opportunistic infections<br>• Consult gastroenterologist	If returns to grade 2:<br>•Taper steroids over at least 1 month<br><br>If does not improve in >3-5 days, worsens or rebounds:<br>• Add mycophenolate mofetil 1 gram (g) twice daily (BID)<br>• If no response within an additional 3-5 days, consider other immunosuppressants per local guidelines

Patients on IV steroids may be switched to an equivalent dose of oral corticosteroids (e.g. prednisone) at start of tapering or earlier, once sustained clinical improvement is observed. Lower bioavailability of oral corticosteroids should be taken into account when switching to the equivalent dose of oral corticosteroids.

*I-O therapy may be delayed rather than discontinued if AST/ALT ≤ 8 x ULN and T.bili ≤ 5 x ULN.

**The recommended starting dose for grade 4 hepatitis is 2 mg/kg/day methylprednisolone IV.

Endocrinopathy Management Algorithm

Rule out non-inflammatory causes. If non-inflammatory cause, treat accordingly and continue I-O therapy. Consider visual field testing, endocrinology consultation, and imaging.

Asymptomatic thyroid stimulating hormone (TSH) elevation →

- **Continue I-O therapy per protocol**
- **If TSH < 0.5 x lower limit of normal (LLN), or TSH > 2 x ULN, or consistently out of range in 2 subsequent measurements: include free thyroxine (fT4) at subsequent cycles as clinically indicated; consider endocrinology consult**

Symptomatic endocrinopathy →

- **Evaluate endocrine function**
- **Consider pituitary scan**

Symptomatic with abnormal lab/pituitary scan:

- **Delay I-O therapy per protocol**
- **1-2 mg/kg/day methylprednisolone IV or by mouth (PO) equivalent**
- **Initiate appropriate hormone therapy**

No abnormal lab/pituitary MRI scan but symptoms persist:

- **Repeat labs in 1-3 weeks / MRI in 1 month**

→ If improves (with or without hormone replacement):

- **Taper steroids over at least 1 month and consider prophylactic antibiotics for opportunistic infections**
- **Resume I-O therapy per protocol**
- **Patients with adrenal insufficiency may need to continue steroids with mineralocorticoid component**

Suspicion of adrenal crisis (e.g. severe dehydration, hypotension, shock out of proportion to current illness →

- **Delay or discontinue I-O therapy per protocol**
- **Rule out sepsis**
- **Stress dose of IV steroids with mineralocorticoid activity**
- **IV fluids**
- **Consult endocrinologist**
- **If adrenal crisis ruled out, then treat as above for symptomatic endocrinopathy**

Patients on IV steroids may be switched to an equivalent dose of oral corticosteroids (e.g. prednisone) at start of tapering or earlier, once sustained clinical improvement is observed. Lower bioavailability of oral corticosteroids should be taken into account when switching to the equivalent dose of oral corticosteroids.

Skin Adverse Event Management Algorithm

Rule out non-inflammatory causes. If non-inflammatory cause, treat accordingly and continue I-O therapy.

Grade of Rash (NCI CTCAE v4)	Management	Follow-up
**Grade 1-2** Covering ≤ 30% body surface area (BSA)*	• Symptomatic therapy (e.g. antihistamines, topical steroids)<br>• Continue I-O therapy per protocol	<u>If persists > 1-2 weeks or recurs:</u><br>• Consider skin biopsy<br>• Delay I-O therapy per protocol<br>• Consider 0.5-1.0 mg/kg/day methylprednisolone IV or oral equivalent. Once improving , taper steroids over at least 1 month, consider prophylactic antibiotics for opportunistic infections, and resume I-O therapy per protocol<br><u>If worsens:</u><br>• Treat as Grade 3-4
**Grade 3-4** Covering >30% BSA; Life threatening consequences*	• Delay or discontinue I-O therapy per protocol<br>• Consider skin biopsy<br>• Dermatology consult<br>• 1.0-2.0 mg/kg/day IV methylprednisolone IV or IV equivalent	<u>If improves to Grade 1:</u><br>• Taper steroids over at least 1 month and add prophylactic antibiotics for opportunistic infections<br>• Resume I-O therapy per protocol

Patients on IV steroids may be switched to an equivalent dose of oral corticosteroids (e.g. prednisone) at start of tapering or earlier, once sustained clinical improvement is observed. Lower bioavailability of oral corticosteroids should be taken into account when switching to the equivalent dose of oral corticosteroids.
*Refer to NCI CTCAE v4 for term-specific grading criteria.

Neurological Adverse Event Management Algorithm

Rule out non-inflammatory causes. If non-inflammatory cause, treat accordingly and continue I-O therapy.

Grade of Neurological Toxicity (NCI CTCAE v4)	Management	Follow-up
**Grade 1**<br>Asymptomatic or mild symptoms; Intervention not indicated	• Continue I-O therapy per protocol	Continue to monitor the patient.<br>If worsens:<br>• Treat as Grade 2 or 3-4
**Grade 2**<br>Moderate symptoms; Limiting instrumental ADL	• Delay I-O therapy per protocol<br>• Treat symptoms per local guidelines<br>• Consider 0.5 to 1.0 mg/kg/day methylprednisolone IV or PO equivalent	If improves to baseline:<br>•Resume I-O therapy per protocol when improved to baseline<br>If worsens:<br>•Treat as Grade 3-4
**Grade 3-4**<br>Severe symptoms; Limiting self-care ADL; Life-threatening	• Discontinue I-O therapy per protocol<br>• Obtain neurology consult<br>• Treat symptoms per local guidelines<br>• 1.0-2.0 mg/kg/day IV methylprednisolone IV or IV equivalent<br>• Add prophylactic antibiotics for opportunistic infections	If improves to Grade 2:<br>• Taper steroids over at least 1 month<br>If worsens or atypical presentation:<br>• Consider IVIG or other immunosuppressive therapies per local guidelines

Patients on IV steroids may be switched to an equivalent dose of oral corticosteroids (e.g. prednisone) at start of tapering or earlier, once sustained clinical improvement is observed. Lower bioavailability of oral corticosteroids should be taken into account when switching to the equivalent dose of oral corticosteroids.

Myocarditis Adverse Event Management Algorithm

Grade of Myocarditis (NCI CTCAE v4)	Management	Follow-up
**Grade 2**<br>Symptoms with mild to moderate activity or exertion	• Delay I-O therapy; hospitalization with cardiac monitoring<br>• Urgent cardiology consultation for evaluation and management<br>○ Troponin and BNP<br>○ ECG ± continuous cardiac monitoring<br>○ Echocardiogram<br>○ Cardiac MRI<br>• Prompt initiation of 2 mg/kg/day methylprednisolone IV or equivalent	• If worsens, intensify treatment according to grade<br>• Upon recovery, taper steroids over at least 1 month with close monitoring of troponin and BNP as well as for new symptoms<br>• Repeat cardiac MRI for post treatment assessment and cardiology follow-up<br>• Retreatment may be considered after recovery and completion of steroid taper
**Grade 3:** Severe with symptoms at rest or with minimal activity or exertion; intervention indicated<br><br>**Grade 4:** Life-threatening consequences; urgent intervention indicated (e.g., continuous IV therapy or mechanical hemodynamic support)	• Permanently discontinue I-O therapy<br>• Hospitalize to intensive cardiac monitoring<br>• Cardiac evaluation to include:<br>– Troponin and BNP monitoring<br>– ECG ± continuous cardiac monitoring<br>– Echocardiogram<br>– Cardiac MRI<br>– Myocardial biopsy if feasible<br>• Immediate initiation of 2 mg/kg/day methylprednisolone IV or 1 g IV bolus<br>• Consider adding a second immunosuppressive agent<br>Additionally, for Grade 4:<br>• Hospitalize/transfer to institution with expertise in intensive cardiac monitoring<br>• Consider ATG as second agent given its immediate effect	• If no improvement, consider additional immunosuppression<br>• Upon recovery, taper steroids over at least 1 month with close monitoring of troponin and BNP as well as for new symptoms<br>• Repeat cardiac MRI for post treatment assessments and cardiology follow-up

Patients on IV steroids may be switched to an equivalent dose of oral corticosteroids (eg, prednisone) at start of tapering or earlier, once sustained clinical improvement is observed. Lower bioavailability of oral corticosteroids should be taken into account when switching to the equivalent dose of oral corticosteroids.

Prophylactic antibiotics should be considered in the setting of ongoing immunosuppression.

ATG = anti-thymocyte globulin; BNP = B-type natriuretic peptide; ECG = electrocardiogram; IV = intravenous; MRI = magnetic resonance imaging
